# Core set of unfavorable events of proximal humerus fracture treatment defined by an international Delphi consensus process

**DOI:** 10.1186/s12891-021-04887-1

**Published:** 2021-11-30

**Authors:** Laurent Audigé, Stig Brorson, Holger Durchholz, Simon Lambert, Fabrizio Moro, Alexander Joeris

**Affiliations:** 1 Research and Development (L.A. and H.D) and Shoulder and Elbow, Surgery (L.A. and F.M.), Schulthess Clinic, Zurich, Switzerland; 2 Department of Orthopedic Surgery and Traumatology, University Hospital of Basel, Basel, Switzerland; 3 Department of Orthopedic Surgery, Centre for Evidence-Based Orthopaedics, Zealand University Hospital, Køge, Denmark; 4 Department of Clinical Medicine, University of Copenhagen, Copenhagen, Denmark; 5 Klinik Gut, St. Moritz, Switzerland; 6 University College London Hospital, London, UK; 7 AO Innovation Translation Center, AO Foundation, Dübendorf, Switzerland

**Keywords:** Shoulder fractures, Proximal humerus fractures, Unfavorable events, Complications, Standardization, Delphi process, Core event set

## Abstract

**Background:**

Proximal humerus fracture (PHF) complications, whether following surgery or nonoperative management, require standardization of definitions and documentation for consistent reporting. We aimed to define an international consensus core event set (CES) of clinically-relevant unfavorable events of PHF to be documented in clinical routine practice and research.

**Methods:**

A Delphi exercise was implemented with an international panel of experienced shoulder trauma surgeons selected by survey invitation of AO Trauma members. An organized list of PHF events after nonoperative or operative management was developed and reviewed by panel members using on-line surveys. The proposed core set was revised regarding event groups along with definitions, specifications and timing of occurrence**.** Consensus was reached with at least a two-third agreement.

**Results:**

The PHF consensus panel was composed of 231 clinicians worldwide who responded to at least one of two completed surveys. There was 93% final agreement about three intraoperative local event groups (device, osteochondral, soft tissue). Postoperative or nonoperative event terms and definitions organized into eight groups (device, osteochondral, shoulder instability, fracture-related infection, peripheral neurological, vascular, superficial soft tissue, deep soft tissue) were approved with 96 to 98% agreement. The time period for documentation ranged from 30 days to 24 months after PHF treatment depending on the event group and specification. The resulting consensus was presented on a paper-based PHF CES documentation form.

**Conclusions:**

International consensus was achieved on a core set of local unfavorable events of PHF to foster standardization of complication reporting in clinical research and register documentation.

**Trial registration:**

Not applicable.

**Supplementary Information:**

The online version contains supplementary material available at 10.1186/s12891-021-04887-1.

## Background

Proximal humeral fractures (PHF) are common fractures and account for 4–6% of all fractures [1, 2]. Associated with the occurrence of osteoporosis, their incidence increases with age, with about 80% of the fractures seen in patients above the age of 65. More than half of PHFs are displaced with reported proportions between 51 and 86% [1, 3]. Several treatment options are available depending on multiple factors. Surgical options include internal fixation with locking plates or intramedullary nails, or replacement of the humeral head with an arthroplasty [2, 3]. Most PHFs however are increasingly managed without surgery.

Evidence-based treatment recommendations, operative or nonoperative, presuppose knowledge on benefits and harms. Validated clinical outcome instruments are available and widely used for reporting treatment effects [4, 5]. However, when it comes to reporting of complications and adverse events (AEs) after management of PHF there is a paucity of standardized and validated terms and definitions [6, 7]. Adverse events were included by international consensus in a preliminary Core Outcome Set (COS) for shoulder disorders [8], further highlighting the need for more standardization [9].

Core event sets (CES) in shoulder disorders were recently developed for arthroscopic rotator cuff repair (ARCR) [10] and shoulder arthroplasty (SA) [11] as the minimum set of events that should be systematically documented and reported in routine care and clinical research. Relevant unfavorable events local (regional) to the operated shoulder were defined by international consensus and organized in a hierarchical structure to facilitate standardization of safety assessment and reporting. The CES for ARCR was successfully piloted in a large retrospective investigation [12]. Events affecting the rest of the body may be agreed on for the whole orthopedic field, however a CES requires to be adjusted for specific pathologies and/or treatments.

The aim of this project was to reach international consensus on a CES specific to various joint-preserving PHF treatments, operative or not.

## Methods

### Delphi methodology and selection of panel members

We applied a similar methodological process for the development of the PHF CES as that used for ARCR [10] and SA [11]. Unfavorable event terms and definitions were identified from a systematic literature review including original articles published between 2010 and 2017, and grouped according to nine previously defined event groups [6, 7]. An initial proposal for the PHF CES was drafted during a meeting of the authors, all members of the steering committee for this project. The CES would apply only to patients affected by unfavorable events that are symptomatic or asymptomatic, the latter however leading to unplanned secondary interventions to prevent symptom development. They were to be distinguished from radiological observations resulting from routine monitoring of PHF recovery.

We applied the modified Delphi technique [13] together with an international panel of experienced shoulder trauma surgeons. In December 2018 an invitation to participate to this project was sent electronically to AO Trauma members along with a link accessing an on-line survey (Supplement file 1) to document personal information, level of expertise in treating PHF and location of practice of interested clinicians. Selection for invitation to the panel required sufficient expertise defined as treating more than 20 PHF annually and having more than 5 years of experience in orthopedic trauma.

Interested and eligible clinicians were invited per email to complete any of two successive surveys using the REDCap online electronic data capture system [14]. For each survey, we sent up to three personal email reminders to minimize the proportion of non-responders. Members of the PHF Consensus Panel acknowledged in this work participated in at least one of these two surveys. They reviewed, commented and made suggestions regarding the proposed CES, as well as a minimum set of parameters for PHF monitoring of all patients. Participants and steering committee members were blinded of the identities of panel members.

### Development of the initial core set and first online survey

The initial CES draft proposal was submitted as part as the initial Delphi survey (Supplementary File 2). Participants were asked about their agreement on the CES development concept including a distinction between intra- and postoperative events. Participants were asked if they agreed on proposed event groups along with their term definitions and specifications, and if these event groups were relevant to various treatment options including nonoperative management, intramedullary nail, plate and other PHF fixation technique. We provided open questions for the formulation of definitions of osteochondral events. We also proposed or asked for the required period of observation for each event group (e.g. 3, 6, 12 or 24 months or lifelong until implant removal). Participants were able to suggest any additions or corrections they felt were necessary using open fields.

### Second and final online survey

Based on initial responses, a second survey was prepared to propose changes to the CES for review, comment and agreement (Supplementary File 3). Again, this second survey considered joint-preserving PHF treatment options only. Event definitions and specifications were amended for all initially proposed event groups, except for postoperative peripheral neurological events and superficial soft tissue events for which consensus was already obtained at the initial survey.

### Data analysis and final adjudication

Intercooled Stata version 14 (StataCorp LLC, College Station, TX) was used for standard descriptive analyses of collected survey data. Missing responses on any question by the participants were not replaced. Consensus was achieved upon agreement of at least two-thirds of the respondents. The required observation period for specific event groups was proposed when at least two-thirds of respondents suggested the same or a shortened period. All comments and suggestions made were listed and reviewed. Final amendments and adjudication of the CES were made by the steering committee if they were considered improvements for correctness, clarity and practical application.

## Results

### Consensus panel

Of all AO Trauma members initially contacted, 331 interested clinicians with sufficient experience in treating PHF were invited per email to participate in the panel. From the initial invitation, 219 clinicians participated in the Delphi process (66%), of which 171 (78%) completed the survey. The second survey was answered by 143 clinicians (44%), of which 128 (90%) completed the survey. The PHF consensus panel was composed of 231 clinicians who responded at least partly to one of the two surveys (Supplementary File 4). They practiced mostly in the European (48.1%) and Asia-Pacific regions (28.1%), followed by North America (11.3%), South America (8.2%) and Africa (4.3%). There were 93 (40%) panel members who reported having more than 20 years orthopedic trauma experience or treating at least 100 PHF annually or both (Table 1).

**Table 1:** Skill of the clinician consensus panel

Average annual PHF^a^	Years of experience^b^			Total
	> 5–10	> 10–20	> 20	
> 20–50	32	59	43	134
> 50–100	22	25	25	72
> 100	3	11	11	25
Total	57	95	79	231

^a^On average, how many proximal humerus fractures (including surgical and non-surgical cases) do you treat annually?

^b^How many years of surgical experience do you have in orthopedic trauma?

### Initial survey

The development framework for this CES was highly supported with 99% (217/219) agreement among the first survey participants. Ninety-seven percent (211/218) of respondents supported a clear distinction between intra- and postoperative events (Supplementary File 5).

Consensus was reached with 97% agreement (206/213) to organize intraoperative events into three distinct event groups (device | osteochondral | soft tissue). Respondents were rather (38%) or definitively (58%) in agreement to distinguish between eight event groups gained from the SA CES [11] (implant [device] | osteochondral | shoulder instability | peripheral neurological | vascular | infection | superficial soft tissue | deep soft tissue). Percentages of agreement for each event group definition and specification ranged from 93 to 99%, except for the osteochondral event group for which respondents provided numerous definitions for a list of specification terms in the context of different treatment options including nonoperative management. Final consensus for both peripheral neurological and superficial soft tissue events groups was reached after the first survey with 97% (165/170) and 98% (165/168) of respondent agreement, respectively, for all treatment options and is presented along with the final overall consensus.

### Second and final survey

An intraoperative event is defined as any event that occurs or is recognized during the time interval between skin incision and skin closure. When the fracture is reduced under anaethesia in the context of nonoperative management, an equivalent “fracture reduction” period was approved by 91% (128/140) of respondents as “the time interval between the patient entered the operating room (OR) and the time the patient exited the OR”. Despite agreement at the first survey, we made the following changes to the event group specifications: cementation problems would occur with augmentation techniques, and nerve lesions were no longer associated with a need for surgical intervention. Final agreement about definitions and specifications of intraoperative event groups reached 93% (128/138) (Table 2).

**Table 2:** Definitions and specifications of intraoperative event groups^a^

Event group	Definition and specification
Device events	Events affecting any component of the implanted device or material, or the instrumentation used for their implantation.
	• Instrument problem (breakage, failure) • Implant (breakage, malpositioning, separation, separation, screw/bolt joint surface perforation requiring immediate postoperative surgical revision) • Cementation problems (augmentation)
Osteochondral events	Events affecting the osteochondral tissue of the proximal humerus, clavicula and/or scapula
	Articular cartilage damage • iatrogenic Fracture (including hairline fracture): humerus metaphyseal (proximal to “surgical neck”); humerus diaphyseal; scapula
Soft tissue events	Events involving only the soft tissue at the treated shoulder
	• Skin, muscle, tendon, joint capsule, ligament, labrum • Blood vessels (bleeding): bleeding at the surgical site that requires additional intervention or leads to a stop of the operation • Nerves^b^: recognized damage of a neurological structure

^a^Adapted from Audige et al. (Audige et al. 2016, Audige et al. 2019). An intraoperative event is any event that occurs or is recognized during the time interval between skin incision and skin closure. When the fracture is reduced under anaethesia in the context of non-operative management, an equivalent “fracture reduction” period is considered as the time interval between the patient entered the operating room (OR) and the time the patient exited the OR

^b^A standard list of potentially affected nerves is only presented for postoperative neurological events. Contrary to the CES in shoulder arthroplasty, damages of neurological structures were not restricted to those which needed additional surgical intervention

A postoperative event is defined as any event that occurs or is recognized during the time interval between the date and time that the patient exited the operation room and the end of the observation period as defined separately for each event group. The proposed changes to the postoperative or nonoperative event terms and definitions organized into eight groups were approved with 96 to 98% agreement (Table 3) [15–18]. The time period for documentation ranged from 30 days to 24 months after PHF treatment depending on the event group and specification. Device events related to nonoperative management would occur during the time the devices (e.g. bandage, splint, plaster) are used.

**Table 3:** Definitions and specifications of postoperative and non-operative event groups^a^

Event groups	Definitions and specifications	Period^b^	Agreement
Implant (device) [postoperative]	Events affecting any device used (e.g. nail, plate, prosthesis, external fixator) which are shown on adequate postoperative imaging (e.g. radiographs, ultrasound, CT) and which are associated with symptoms • Malpositioning ^c^: implant not in its expected position • Radiolucency around the implant / Implant loosening: radiolucency relates to the occurrence/observation of radiolucent lines (RLL) at the bone-implant interface • Screw or bolt backout • Implant breakage: one part of the implant is broken • Migration: change of the position of an implant component relative to the bone it is supposedly fixed to	12 months	98% (125/128)
Device [non-operative]	Events (e.g. breakage, loosening) involving any external device (e.g. sling, orthosis) used to immobilize the arm to support the fracture, which is associated with local clinical symptoms (e.g. local reactions such as skin lesions).	Time during use of the device(s)	
Osteochondral	Events affecting the osteochondral tissue of the proximal humerus, clavicula and/or scapula Surgical treatment only: • New fracture (around the implant) • Screw / bolt cutout ^d^ All treatment interventions: • Bone formation / resorption (except head necrosis and tuberosity resorption) • Tuberosity migration / resorption • Head necrosis • Delayed union / nonunion • Loss of fracture reduction (except tuberosity migration)	24 months	97% (122/126)
Shoulder instability	symptomatic shoulder associated with loss of alignment of the articulating surface of the humeral head with the glenoid surface • Subluxation: non arm position-dependent eccentric misalignment with residual contact. • Dislocation: non arm position-dependent complete loss of contact of the articulating surfaces. • Dynamic instability: arm position-dependent loss of contact of the articulating surfaces apparent on physical examination and/or visible on functional radiographs (horizontal flexion/extension view in 90° of abduction and true anteroposterior (AP) view in 60° of abduction).	12 months	96% (121/126)
Peripheral neurological	Events resulting from peripheral neurological injury at the fracture site, which is associated with sensory and/or motor and/or autonomic disturbance • Sensory and/or motor disturbance: Affected nerve(s) -Cervical or brachial plexus -Branch neuropathy (suprascapular, musculocutaneous, median, ulnar, radial, axillary, dorsal scapular, long thoracic, spinal accessory, thoracodorsal, cutaneous nerves of arm and forearm) • Autonomic disturbance: Complex regional pain syndrome (CRPS) Neurological injury may be classified by a neurologist according to Seddon ^15^ (i.e. neurapraxia, axonotmesis, neurotmesis) and/or Birch ^16^ (degenerative, short conduction block, prolonged condition block)	3 months	97% (165/170) ^e^
Vascular	Events involving laceration, avulsion, contusion, puncture or crush injury to an artery or vein at the injured arm • Hematoma which requires evacuation by needle or surgery • Superficial and deep thrombosis at the involved extremity • Ischemia of the involved extremity which requires additional intervention	30 days	98% (124/127)
Infections	Fracture-related Infections (FRI) ^f^ Definition of terms and specifications adopted from a 2018 FRI consensus definition ^17^	24 months	98% (124/127)
Superficial soft tissue	Events affecting the superficial soft tissues (i.e. skin and subcutaneous tissue) at and around the surgical site/wound that do not affect deep soft tissues (i.e. fascia, muscle, articular capsule) and that require additional treatment • Early events 30 days: edema; emphysema; burn; delayed wound healing; hypersensitivity reaction; skin necrosis; skin bulla • Late events within the first 6 months: hypertrophic scar and keloid (except if known history of previous development)	30 days to 6 months	98% (165/168) ^e^
Deep soft tissue	Events affecting the deep soft tissues (i.e. fascia, muscle, articular capsule), except infections • External muscular envelope: deltoid-pectoralis major • Subacromio-deltoid-coracoid bursa (space) • Rotator cuff muscle-tendon and biceps tendon • Capsule-synovium	12 months	97% (122/126)

^a^adapted from Audige et al. (Audige et al. 2016, Audige et al. 2019) for proximal humerus fractures and their joint-preserving treatment modalities. Unless otherwise specified, the defined event group definition and specifications relate to both postoperative and non-operative events

^b^none of the considered events in the core set must be present or occur prior to or at the time of trauma. Hence they are to be distinguished from concomitant lesions directly resulting from the trauma

^c^may result from intraoperative malpositioning and/or postoperative implant displacement. The time of occurrence may be determined by immediate postoperative assessment of the implant position

^d^may be associated with loss of fracture reduction (e.g. head collapse) and/or head necrosis

^e^Level of agreement achieved already at the first survey

^f^Despite a high level of agreement at the first Delphi survey (93%; 160/172) to adopt the definition and specifications adapted from the 2008 Centers for Disease Control and Prevention (CDC) definition ^18^, the steering committee suggested at the second survey that a recently published consensus on “fracture-related infection” ^17^ should be adopted

Device events related to implants include malpositioning, radiolucency and loosening, screw or bolt backout, breakage and migration (Table 3). Events related to external devices used to immobilize the affected arm such as breakage and loosening are to be documented only when associated with clinical symptoms. Specific osteochondral events were listed as bone formation/resorption, tuberosity migration/resorption, head necrosis, delayed union/nonunion, and loss of fracture reduction. Additionally, the events “fracture around the implant” and “screw/bolt cutout” were considered in the context of surgical treatment. Despite a high level of agreement at the first Delphi survey (93%; 160/172) to adopt the definition and specifications adapted from the 2008 Centers for Disease Control and Prevention (CDC) definition [18], respondents agreed also to adopt the consensus on “fracture-related infection” [17]. The deep soft tissue event group was reorganized in 4 categories according to the involved anatomical structures: the external muscular envelope (deltoid-pectoralis major), the subacromio-deltoid-coracoid bursa (space), the rotator cuff muscle-tendon and biceps tendon, and finally the capsule-synovium.

## Discussion

This project focused on the development of a core set of unfavorable local events for PHF in the context of joint-preserving treatment options. We used a modified Delphi process and reached widespread consensus among 231 experienced shoulder trauma surgeons after two online surveys with 96–98% agreement for specific event groups. Should a PHF be treated by arthroplasty, the CES in SA [11] can be used. The present CES is an adaptation for all other PHF treatment options. It has high face validity among trauma shoulder specialists and therefore represents a consolidated proposal for application and evaluation in clinical practice and research.

The term “complication” is often used in clinical practice without defining it, so the CES should be better understood as a list of “unfavorable event” that are considered clinically relevant for clinicians, patients, or both. A CES represents a minimum set of events that should be monitored and reported in all PHF treatment. Additional events may be defined and added to the list (possibly also within predefined CES event groups) for specific studies; this approach should preserve standardization and transparency of reporting safety outcomes.

The periods of observation defined for each event groups, or specific events, are important because they stipulate that a minimum follow-up period of 24 months is required for PHF treatment in order to generate valid safety data. Many published reports on PHF management consider a final follow-up at 12 months [3], which may be appropriate to assess treatment effectiveness (e.g. pain level, functional outcome, return to some level of self-dependence, …), but may be insufficient regarding safety outcomes. The minimum period of follow-up of 24 months defined by the CES may even be extended at the discretion of the respective investigators to allow capturing symptomatic events that can occur later, such as avascular head necrosis. Also, none of the considered events in the core set must be present prior to the time of trauma or occur in the period between trauma and initiation of treatment. Hence, they are to be distinguished from concomitant lesions directly resulting from the trauma or developing before treatment can be initiated. This is particularly important regarding neurovascular damage that may be present at the time of injury. It should be described prior to any attempt to reduce the fracture in order to record iatrogenic damage. Similarly, the PHF pattern and severity should be adequately documented prior to treatment. For example, a 2-part anterior fracture-dislocation can occasionally be iatrogenically converted to a 3-part or 4-part fracture during reduction.

While many terms are used in the literature to describe similar or associated events or conditions [6, 7], some choices and decisions were made on what was perceived the most relevant terms that could be understood by the majority of clinicians (however not necessarily all stakeholders like the patients themselves). Some terms were conscientiously avoided. For instance, the event term “fracture malunion” was supported by a large majority of participants during the first survey, however, was subsequently no longer considered by the steering committee at the second survey. We perceived that the term reflects rather negative or inadequate performance, although all PHFs may be considered somehow “malreduced” whatever their treatments. In nonoperative management of displaced fractures, in particular, one would expect and accept some degree of “malunion”. What amount of malunion is to be tolerated for each patient is not well defined, notably in view of the poor documented correlation between grade of “anatomical” reduction and functional outcome. While some guidelines would be very useful, they remained outside the scope of the present CES development. Also the term “screw cutout” was not considered as a leading unfavorable event in PHF internal fixation, but as a result of a collapse of the humeral head (due to head necrosis and/or loss of fracture reduction), and therefore such event was proposed within the osteochondral event group.

In previous CES development there was an overwhelming consistent agreement that the infection definition of the CDC should be used [10, 11]. This was confirmed at the first Delphi survey with 93% agreement among respondents. Later, an international consensus definition of fracture-related infection (FRI) has emerged [17], which was approved by 98% of the panel. We believe that it is essential to strive for consistency and avoid the development of parallel definition systems that could lead to confusion and limit effective application in practice. The more recent definition of FRI is a helpful simplification: “for the purposes of a definition (and data collection), it is important that surgeons define the presence of infection, not its extent, localization or classification” [17].

This project has limitations as outlined in previous similar reports. The response rate was only 44% in the second and final Delphi survey, which brings a possibility of a biased final agreement when non-respondents had disagreed during the initial survey. Given the very high final level of consensus agreement reached, we consider such bias as limited or negligible in this project. We provided detailed reports of respondents’ feedback in the second survey, including all suggestions and comments, which should enhance the quality and the relevance of final responses and consensus decisions for the CES [19].

Patients were not involved in the panel, although this is increasingly advocated in the context of core outcome set development [20] to increase its generalizability, credibility, and uptake in clinical practice and research. While involving patients in this project would have limited the risk of overlooking patient-outcome relevant events, a Delphi exercise is difficult to implement when mixing clinicians (experts) and patients (nonexperts); in the context of this type of enquiry it is reasonable not to include patients’ input [21]. Our view is that patient involvement in CES development is better considered in a subsequent clinical implementation and assessment, when it can be also documented which events matter most to patients.

To support uniform clinical application of the present “PHF core event set 1.0”, we developed a paper-based PHF complication form (Supplement File 7). When several unfavorable events occur and are managed simultaneously in any patient, the leading event should be primarily recorded according to the clinician’s judgement. There is no predefined hierarchy of events for PHF.

## Conclusions

We developed a CES in the context of PHF treatment by international consensus, which reached very high panel agreement and face validity. We believe it might contribute to the standardization of reporting unfavorable events in this field if widely applied in practice and research.

## Supplementary Information

**Additional file 1.** PHF Core Event Set v1.0 - Invitation survey screenshots.

**Additional file 2.** PHF Core Event Set v1.0 - Delphi 01 survey screenshots.

**Additional file 3.** PHF Core Event Set v1.0 - Delphi 02 survey screenshots.

**Additional file 4.** PHF CES Consensus Panel (231 participants in alphabetical order).

**Additional file 5.** PHF Core Event Set v1.0 - Delphi 01 survey results – July 2019.

**Additional file 6.** PHF Core Event Set v1.0 - Delphi 02 survey results – June 2020.

**Additional file 7.** PHF Core Event Set v1.0 – Paper-based Form.

## Data Availability

The survey datasets collected and analyzed during the current study are available from the corresponding author on reasonable request.

## Abbreviations

AO: Arbeitsgemeinschaft für Osteosynthesefragen (German for “working group for bone fusion issues”)
ARCR: Arthroscopic rotator cuff repair
CDC: Centers for Disease Control and Prevention
CES: Core event set
COS: Core outcome set
FRI: Fracture-related infection
OR: Operating room
PHF: Proximal humerus fracture
REDCap: Research electronic data capture
SA: Shoulder arthroplasty

## References

[1] Court-Brown CM, Garg A, McQueen MM (2001). The epidemiology of proximal humeral fractures. Acta Orthop Scand.

[2] Brorson S, Palm H, Falaschi P, Marsh D (2021). Proximal Humeral Fractures: The Choice of Treatment. Orthogeriatrics: The Management of Older Patients with Fragility Fractures.

[3] Brorson S (2017). Proximal Humeral Fractures. Musculoskeletal Trauma in the Elderly.

[4] Zuchermann J, Park S, Zuckerman J, Koval K (2005). Principles of treatment and outcome assessment. Shoulder Fractures: The Practical Guide to Management.

[5] Slobogean GP, Noonan VK, O'Brien PJ (2010). The reliability and validity of the disabilities of arm, shoulder, and hand, EuroQol-5D, health utilities index, and short form-6D outcome instruments in patients with proximal humeral fractures. J Shoulder Elb Surg.

[6] Alispahic N, Brorson S, Bahrs C, Joeris A, Steinitz A, Audige L (2020). Complications after surgical management of proximal humeral fractures: a systematic review of event terms and definitions. BMC Musculoskelet Disord.

[7] Brorson S, Alispahic N, Bahrs C, Joeris A, Steinitz A, Audige L (2019). Complications after non-surgical management of proximal humeral fractures: a systematic review of terms and definitions. BMC Musculoskelet Disord.

[8] Buchbinder R, Page MJ, Huang H, Verhagen AP, Beaton D, Kopkow C, Lenza M, Jain NB, Richards B, Richards P (2017). A preliminary Core domain set for clinical trials of shoulder disorders: a report from the OMERACT 2016 shoulder Core outcome set special interest group. J Rheumatol.

[9] Audige L, Goldhahn S, Daigl M, Goldhahn J, Blauth M, Hanson B (2014). How to document and report orthopedic complications in clinical studies? A proposal for standardization. Arch Orthop Trauma Surg.

[10] Audige L, Flury M, Muller AM, Durchholz H, ARCR CES Consensus Panel (2016). Complications associated with arthroscopic rotator cuff tear repair: definition of a core event set by Delphi consensus process. J Shoulder Elb Surg.

[11] Audige L, Schwyzer HK, Durchholz H, Shoulder Arthroplasty Core Event Set Consensus Panel (2019). Core set of unfavorable events of shoulder arthroplasty: an international Delphi consensus process. J Shoulder Elb Surg.

[12] Felsch Q, Mai V, Durchholz H, Flury M, Lenz M, Capellen C, Audige L (2021). Complications within 6 months after arthroscopic rotator cuff repair: registry-based evaluation according to a Core event set and severity grading. Arthroscopy.

[13] Hsu CC, Standford BA (2007). The Delphi technique : making sense of consensus.

[14] Harris PA, Taylor R, Thielke R, Payne J, Gonzalez N, Conde JG (2009). Research electronic data capture (REDCap)--a metadata-driven methodology and workflow process for providing translational research informatics support. J Biomed Inform.

[15] Seddon HJ (1942). A classification of nerve injuries. Br Med J.

[16] Birch R (2011). Clinical aspects of nerve injury. Surgical disorders of the peripheral nerves.

[17] Metsemakers WJ, Morgenstern M, McNally MA, Moriarty TF, McFadyen I, Scarborough M, Athanasou NA, Ochsner PE, Kuehl R, Raschke M (2018). Fracture-related infection: a consensus on definition from an international expert group. Injury.

[18] Horan TC, Andrus M, Dudeck MA (2008). CDC/NHSN surveillance definition of health care-associated infection and criteria for specific types of infections in the acute care setting. Am J Infect Control.

[19] Fish R, MacLennan S, Alkhaffaf B, Williamson PR (2020). “Vicarious thinking” was a key driver of score change in Delphi surveys for COS development and is facilitated by feedback of results. J Clin Epidemiol.

[20] Chevance A, Tran VT, Ravaud P (2020). Controversy and Debate Series on Core Outcome Sets. Paper 1: improving the generalizability and credibility of core outcome sets (COS) by a large and international participation of diverse stakeholders. J Clin Epidemiol.

[21] Domecq JP, Prutsky G, Elraiyah T, Wang Z, Nabhan M, Shippee N, Brito JP, Boehmer K, Hasan R, Firwana B (2014). Patient engagement in research: a systematic review. BMC Health Serv Res.

