# Supplementary material for: Core set of unfavorable events of proximal humerus fracture treatment defined by an international Delphi consensus process
Source: BMC Musculoskelet Disord. 2021 Nov 30;22:1002. doi: 10.1186/s12891-021-04887-1 (PMC8630858; doi:10.1186/s12891-021-04887-1)
Supplement: Supplementary file 1 — Additional file 1. PHF Core Event Set v1.0 - Invitation survey screenshots. [file 12891_2021_4887_MOESM1_ESM.pdf]

AO TRAUMA

Supplementary file 1

**Article title**	Core set of unfavorable events of proximal humerus fracture treatment defined by an international Delphi consensus process
**Journal name**	BMC Musculoskeletal Disorders
**Author names**	Audigé L, Brorson S, Durchholz H, Lambert S, Moro F, PHF CES Consensus Panel, Joeris A
**Affiliation**	Schulthess Klinik, CH-8008 Zurich, Switzerland
**E-mail address**	laurent.audige@kws.ch

PHF Core Event Set v1.0

Core list of unfavorable events of proximal humerus fracture (PHF)

Invitation survey screenshots

Consensus development of a core list of proximal humerus fracture complications (panel selection)

Dear Colleague,

We are initiating a consensus Delphi process to define a core set of unfavorable events to be documented and evaluated during and after treatment of proximal humerus fractures (PHFx), whether operative or non-operative.

We are searching experienced shoulder trauma surgeon treating PHFx who may be interested to participate. Participation will involve responding up to 3 on-line surveys of about 15-20 minutes within a period of about one year. All participants will be acknowledged in publications with a project group name.

If you are interested to participate, we invite you to complete the following questionnaire.

We thank you for your expertise and support.

Kind regards

The project steering committee

This project is based on a similar development that was recently completed for events of arthroscopic rotator cuff repair* and shoulder arthroplasty**

**Audigé L, Flury M, Müller AM, ARCR CES Consensus Panel, Durchholz H. Complications associated with arthroscopic rotator cuff tear repair: definition of a core event set by Delphi consensus process. Journal of Shoulder and Elbow Surgery 2016; 25:1907-1917. doi: 10.1016/j.jse.2016.04.036*

***Audigé L., Salomonsson B., Moroder P., Lambert S., Sperling J., Page R., Schwyzer H.K., SA CES Consensus Panel, Durchholz H. International consensus for minimum radiological monitoring and complication reporting in shoulder arthroplasty: completion of a Delphi process, International Society of Arthroplasty Registers (ISAR) meeting, Reykjavik, Iceland, June 09-11, 2018 (manuscript in preparation)*

We aim to select an international panel of 80-100 shoulder trauma specialists having each more than 5 years of experience and treating more than 20 proximal humerus fractures annually

If you are selected, would you agree to participate in this Delphi consensus project? ◯ Yes ◯ No

reset

Submit

Save & Return Later

Level of experience in PHFx

	1-5 years	>5-10 years	>10-20 years	>20 years
**How many years of surgical experience do you have in orthopedic trauma?**	○	○	○	○

reset

	1-20	>20-50	>50-100	>100
**On average, how many proximal humerus fractures do you treat annually?** (including surgical and non-surgical cases)	○	○	○	○

reset

Affiliation and Personal Contact Data

Hospital / institution address

Name of hospital / institution

Department (eg. Orthopeadics, Trauma, Surgery)

Address (Street and No.)

Expand

Postal Code / ZIP

City

Country

Region

- ○ North America
- ○ South America
- ○ Europe
- ○ Africa
- ○ Asia/Pacific

reset

Is your hospital / institution a referral center for others? ○ Yes ○ No

reset

Personal information

Title

- ◯ Dr.
- ◯ PD Dr.
- ◯ Prof Dr.
- ◯ Mr
- ◯ Mrs
- ◯ Other

reset

Name

Surname

Academic qualifications

- ☐ MD
- ☐ PhD
- ☐ PD
- ☐ MPH
- ☐ MSc
- ☐ MBA
- ☐ Other

Please check all that apply

Email

Comment

Do you have any comment(s)?

Expand

Submit

Save & Return Later
