# Supplementary material for: Core set of unfavorable events of proximal humerus fracture treatment defined by an international Delphi consensus process
Source: BMC Musculoskelet Disord. 2021 Nov 30;22:1002. doi: 10.1186/s12891-021-04887-1 (PMC8630858; doi:10.1186/s12891-021-04887-1)
Supplement: Supplementary file 2 — Additional file 2. PHF Core Event Set v1.0 - Delphi 01 survey screenshots. [file 12891_2021_4887_MOESM2_ESM.pdf]

Supplementary file 2

Article title Core set of unfavorable events of proximal humerus fracture treatment defined by an international Delphi consensus process

Journal name BMC Musculoskeletal Disorders

Author names Audigé L, Brorson S, Durchholz H, Lambert S, Moro F, PHF CES Consensus Panel, Joeris A

Affiliation Schulthess Klinik, CH-8008 Zurich, Switzerland

E-mail address laurent.audige@kws.ch

PHF Core Event Set v1.0

Core list of unfavorable events of proximal humerus fracture (PHF)

Delphi 1 survey screenshots

Resize font:

Returning?

Consensus development of a core list of unfavorable events of proximal humerus fracture treatment (survey 1)

Dear Colleague,

Thank you for your participation in this Delphi consensus project.

Please make sure that you answer all questions and complete the survey. You may leave the survey and return to it where it was left at any time. In such case take note of the provided code to allow returning and completing the survey. If you lose this code, you may contact me.

Kind regards

Prof Dr. Laurent Audigé, PhD on behalf of the steering committee

This online survey is the first as part of a modified Delphi exercise, which will possibly include up to 3 surveys in total. It is based on a similar development that was recently completed for events of arthroscopic rotator cuff repair* as well as events of shoulder arthroplasty**

**Audigé L, Flury M, Müller AM, ARCR CES Consensus Panel, Durchholz H. Complications associated with arthroscopic rotator cuff tear repair: definition of a core event set by Delphi consensus process. Journal of Shoulder and Elbow Surgery 2016, 25(12): 1907-1917*

***Audigé L, Schwyzer HK, SA CES Consensus Panel, Durchholz H. Core set of unfavorable events of shoulder arthroplasty: an international Delphi consensus process. Poster presented at ISAR 2018. Manuscript in preparation.*

Attachment: Audige-2016-JSES-Complications associated with ARCR.pdf (0.91 MB)

Do you agree to participate in this consensus development process? ○ Yes ○ No reset

* must provide value

Next Page >>

Save & Return Later

Consensus development of a core list of unfavorable events of proximal humerus fracture treatment (survey 1)

Conceptual development of the core set

This core event set development is based on a **conceptual framework** from previous experiences with similar projects as well as new considerations resulting from a literature review of unfavorable events of proximal humerus fracture (PHFx) treatment. Note that we will avoid the term "complication" in this survey, because it remains undefined.

In the field of PHFx, we are considering operative as well as non-operative treatment options. Unfavorable events therefore may be common to all PHFx or specific following one or more treatment options. Treatment options include joint-preserving fracture fixation (mostly by plating or intramedullary nailing) and joint replacement by arthroplasty. A consensus was recently achieved regarding a core set of events in shoulder arthroplasty (SA), therefore we plan to address the detailed event specifications in SA in a subsequent survey.

We are focusing on **local (regional) unfavorable events** affecting the treated shoulder, which are considered in a hierarchical structure involving event groups and specifications.

In the context of operative treatment, we distinguish further **intraoperative** and **postoperative** events.

Nonlocal events affecting the rest of the body are addressed globally for all orthopedic interventions because they are not specific to PHFx.

The proposed structural documentation is purely descriptive without inferring on any influencing factor(s) for the event(s). In addition the events themselves are distinguished from their treatment (e.g. a re-operation or revision orperation) and outcome (e.g. disability or death). Unfavorable events in the core set may not include all adverse events as defined by regulation; also they may not be all considered treatment complications

Audige et al. How to document and report orthopedic complications in clinical studies? A proposal for standardization. Arch Orthop Trauma Surg 2014, 134(2): 269-275

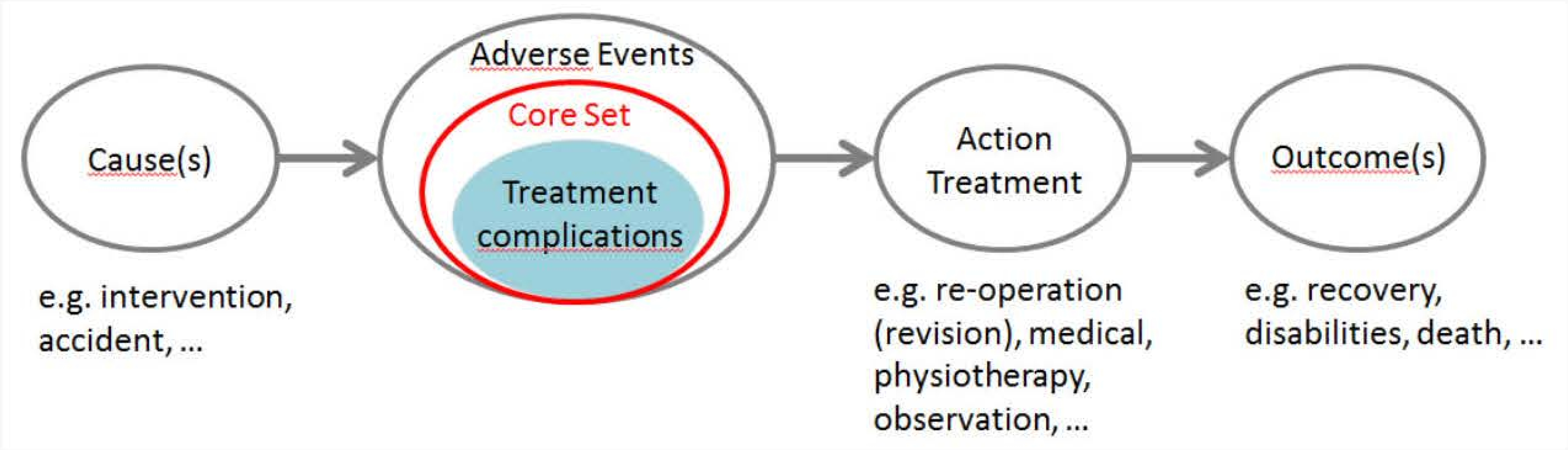

Do you agree with this concept and developmental process? ○ Yes ○ No

reset

Comment or suggestion

Expand

<< Previous Page   Next Page >>

Save & Return Later

Resize font:

Consensus development of a core list of unfavorable events of proximal humerus fracture treatment (survey 1)

Intraoperative versus postoperative events

An intraoperative event is any event that occurs or is recognized during the time interval between skin incision and skin closure*.

A postoperative event is any event that occurs or is recognized during the time interval between the date and time (to the nearest minute) that the patient exited the operation room and the end of the observation period**.

adapted by Audige et al. JSES 2016, 25(12): 1907-1917 from ...
Jacobs JP et al. The Annals of Thoracic Surgery 2007, 84(4): 1416-1421
Rosenthal et al. World Journal of Surgery 2015, 39:1663-1671

* All events occurring between the date and time that the patient entered the operating room (OR) and the date and time when the patient exited the OR should be considered intraoperative events according to Jacobs et al (2007), yet relevant intraoperative events in the core set would occur during the surgical procedure.

** The end of the observation period is defined for each event or group of events in the core set

Do you agree with this distinction between intra- and postoperative events? ○ Yes ○ No

reset

Comment or suggestion

Expand

<< Previous Page   Next Page >>

Save & Return Later

Resize font:

Consensus development of a core list of unfavorable events of proximal humerus fracture treatment (survey 1)

Intraoperative local events

Device events

Definition : Events affecting any component of the implanted device or material, or the instrumentation used for their implantation.

Specifications :
- Instrument problem (breakage, failure)
- Implant (breakage, malpositioning, separation)
- Screw / bolt joint perforation
- Cementation problem

Osteochondral events

Definition : Events affecting the osteochondral tissue of the proximal humerus, clavicula and/or scapula

Specifications :
- Fracture (including hairline fracture)
- Articular cartilage damage

Soft tissue events

Definition : Events involving only the soft tissue at the treated shoulder

Specifications :
- Skin, muscle, tendon, joint capsule, ligament, labrum
- Blood vessels (bleeding) : bleeding at the surgical site that requires additional intervention or leads to a stop of the operation
- Nerves*: recognized damage of a neurological structure which needs additional surgical intervention

* a standard list of potentially affected nerves will only be presented for postoperative neurological events

Do you agree with these definitions and specifications of intraoperative events? ○ Yes ○ No reset

Comment or suggestion

Expand

<< Previous Page Next Page >>

Save & Return Later

Consensus development of a core list of unfavorable events of proximal humerus fracture treatment (survey 1)

Radiological parameter(s) and monitoring

Some radiological parameters regarding fracture reduction and healing may be relevant to monitor, although they may not necessarily be considered unfavorable events.

	Definitely not	Rather not	Undecided	Rather yes	Yes, definitely
**Do you think this project should define a core set of radiological parameters as a way to monitor fracture reduction and healing?**	○	○	○	○	◉

reset

Do you agree that fracture "malreduction" would be one of such parameters? ◉ Yes ○ No

reset

How would you define proximal humerus fracture "malreduction"?

Expand

Should imaging other than standard radiographs be considered for monitoring fracture reduction and healing? ◉ Yes ○ No

reset

Please specify these other imaging techniques

Which other radiological parameters should we consider?

Expand

Comment or suggestion

Expand

<< Previous Page   Next Page >>

Save & Return Later

Consensus development of a core list of unfavorable events of proximal humerus fracture treatment (survey 1)

Postoperative / non-operative local events (1)

We propose to structure local events according to the following event groups:

1- Implant (device) events: Events affecting any implanted device (e.g. nail, plate, prosthesis) which are shown on adequate postoperative imaging (e.g. radiographs, ultrasound, CT) or affecting any external device (e.g. sling, orthesis) used to immobilize the fracture, which is associated with clinical symptoms.

2- Osteochondral events: Events affecting the osteochondral tissue of the proximal humerus, clavicula and/or scapula

3- Shoulder instability : Symptomatic shoulder associated with loss of alignment of the articulating surfaces

4- Peripheral neurological events: Events resulting from peripheral neurological injury at the fracture site, which are associated with sensory and/or motor and/or autonomic disturbance

5- Vascular events: Events involving laceration, avulsion, contusion, puncture or crush injury to an artery, vein or microvascularity at the fracture site.

6- Infections: Surgical Site Infections (SSI) with definition and specifications adapted from the 2008 Centers for Disease Control and Prevention (CDC) definition AND, in shoulder arthroplasty, late hematogenous infections with periprosthetic infections defined according to international consensus

7- Superficial soft tissue events: Events affecting the superficial soft tissues (i.e. skin and subcutaneous tissue) at and around the fracture site/wound that do not affect deep soft tissues (i.e. fascia, muscle, articular capsule) and that require additional treatment

8- Deep soft tissue events: Events affecting the deep soft tissues (i.e. fascia, muscle, articular capsule), except infections

Notes

None of the considered events in the core set must be present or occur prior to or at the time of trauma. Hence they are to be distinguished from concomittent lesions directly resulting from the trauma.

Some of these event groups may not apply to specific treatment modalities. This will be determined in the following sections of this survey.

	Definitely not	Rather not	Undecided	Rather yes	Yes, definitely
**Do you agree with these event groupings?**	○	○	○	○	○

reset

Comment or suggestion for change

Expand

<< Previous Page   Next Page >>

Save & Return Later

Consensus development of a core list of unfavorable events of proximal humerus fracture treatment (survey 1)

Postoperative local events (2)

Implant (device) events

Definition : Events affecting any implanted device (e.g. nail, plate) which are shown on adequate postoperative imaging (e.g. radiographs, ultrasound, CT), which is associated with clinical symptoms

Specifications :
- Malpositioning*: implant not in its expected position
- Radiolucency around the implant / Implant loosening
- Screw or bold backout
- Implant breakage
- Implant migration (subsidence, tilt, shift): change of the position of a component, relative to the bone it supposedly fixed to

*A malpositioned implant may result from intraoperative malpositioning and/or postoperative implant displacement. The time of occurrence may be determined by immediate postoperative assessment of the implant position

Do you agree with this definition, specifications and terminology?

○ Yes ○ No

reset

Which timeline is suitable for documenting postoperative implant events in internal fixation (e.g. nail, plate)?

○ 3 months
○ 6 months
○ 12 months
○ 24 months
○ lifelong until implant removal
● other period

reset

Specify the other time period

Comment or alternative suggestion

Expand

<< Previous Page

Next Page >>

Save & Return Later

Consensus development of a core list of unfavorable events of proximal humerus fracture treatment (survey 1)

Non-operative local events (3)

Device events

Definition : Events any external device (e.g. sling, orthesis) used to immobilize the fracture, which is associated with clinical symptoms

Observation period : Time during which the immobilization device(s) is(are) in use.

Do you agree with this definition and timing? ○ Yes ○ No

reset

Which specific device event(s) should be documented in the context of non-operative treatment?

Expand

Comment or alternative suggestion

Expand

<< Previous Page

Next Page >>

Save & Return Later

Consensus development of a core list of unfavorable events of proximal humerus fracture treatment (survey 1)

Postoperative / non-operative local events (4)

Osteochondral events (1)

Definition : Events affecting the osteochondral tissue of the proximal humerus, clavicula and/or scapula

For which treatment option(s) the following specific event(s) should be documented? (several options may apply)

	Non-operative management	Intramedullary nail	Plating	Other PHFx fixation	None
**Bone formation / resorption**	☑	☑	☑	☑	☐
**New fracture (around the implant)**	☐	☑	☑	☑	☐
**Screw / bolt cutout**	☑	☑	☑	☑	☐
**Tuberosity migration / resorption**	☑	☑	☑	☑	☐
**Head necrosis**	☑	☑	☑	☑	☐
**Delayed union / nonunion**	☑	☑	☑	☑	☐
**Fracture malunion**	☑	☑	☑	☑	☐
**Loss of fracture reduction**	☑	☑	☑	☑	☐
**Other event(s)**	☑	☑	☑	☑	☐

Specify the other event(s)

Expand

<< Previous Page

Next Page >>

Save & Return Later

Consensus development of a core list of unfavorable events of proximal humerus fracture treatment (survey 1)

Postoperative / non-operative local events (5)

Osteochondral events (2)

How would you define and/or specify (please cite references as appropriate) ...

... bone resorption / bone formation?

Expand

... fractures (around the implant)?

Expand

... screw / bolt cutout?

Expand

... tuberosity migration / resorption?

Expand

... head necrosis?

Expand

... delayed / non-union?

Expand

... malunion?

Expand

... loss of fracture reduction?

Expand

<< Previous Page

Next Page >>

Save & Return Later

Consensus development of a core list of unfavorable events of proximal humerus fracture treatment (survey 1)

Postoperative / non-operative local events (6)

Osteochondral events (3)

Which timeline is suitable for documenting osteochondral events?

	6 months	12 months	24 months	lifelong until implant removal	other time period
... in joint-preserving fracture fixation?	○	○	○	○	◉

reset

Specify the other time period

	6 months	12 months	24 months	other time period
... in non-operative treatment?	○	○	○	◉

reset

Specify the other time period

<< Previous Page

Next Page >>

Save & Return Later

Consensus development of a core list of unfavorable events of proximal humerus fracture treatment (survey 1)

Postoperative / non-operative local events (7)

Shoulder instability

	All	Non-operative treatment	Intramedullary nail	Plate	Other PHFx fixation	None
**For which treatment option(s) should shoulder instability be documented?**	☑	☐	☐	☐	☐	☐

Several options may apply

Definition of terms : symptomatic shoulder associated with loss of alignment of the articulating surface of the humeral component with the articulating surface of its joint partner

Specifications :
- Subluxation : non arm position-dependent eccentric misalignment with residual contact.
- Dislocation : non arm position-dependent complete loss of contact of the articulating surfaces.
- Dynamic instability : arm position-dependent loss of contact of the articulating surfaces apparent on physical examination and/or visible on functional radiographs (horizontal flexion/extension view in 90° of abduction and true AP view in 60° of abduction).

Do you agree with this definition, specifications and terminology? ○ Yes ○ No

reset

Which timeline is suitable for documenting shoulder instability?

	6 months	12 months	24 months	lifelong until implant removal	other time period
**... in joint-preserving fracture fixation?**	○	○	○	○	◉

reset

Specify the other time period

	6 months	12 months	24 months	other time period
**... in non-operative treatment?**	○	○	○	◉

reset

Specify the other time period

<< Previous Page   Next Page >>

Save & Return Later

Consensus development of a core list of unfavorable events of proximal humerus fracture treatment (survey 1)

Postoperative / non-operative local events (8)

Peripheral neurological events

	All	Non-operative treatment	Intramedullary nail	Plate	Other PHFx fixation	None
**For which treatment option(s) should neurological events be documented?** Several options may apply	☑	☐	☐	☐	☐	☐

Definition : Events resulting from peripheral neurological injury at the fracture site, which is associated with sensory and/or motor and/or autonomic disturbance

Specifications :
Sensory and/or motor disturbance: Affected nerve(s)
- Cervical or brachial plexus
- Branch neuropathy (suprascapular, musculocutaneous, median, ulnar, radial, axillary, dorsal scapular, long thoracic, spinal accessory, thoracodorsal, cutaneous nerves of arm and forearm).

Autonomic disturbance: Complex regional pain syndrome (CRPS)

An optional injury classification can be documented via neurologist according to Seddon* (i.e. neurapraxia, axonotmesis, neurotmesis) and/or Birch** (degenerative, short conduction block, prolonged condition block)

** Seddon H. (1942) A classification of nerve injuries. British Medical Journal, 2(4260): 237-239.*
*** Birch R. (2010) Clinical aspects of nerve injury, in Surgical disorders of the peripheral nerves. Springer. 145-190*

Period of observation: 3 months

Do you agree with this definition, specifications and terminology? ○ Yes ○ No reset

Comment or alternative suggestion

Expand

<< Previous Page   Next Page >>

Save & Return Later

Resize font:

Consensus development of a core list of unfavorable events of proximal humerus fracture treatment (survey 1)

Postoperative / non-operative local events (9)

Vascular events

	All	Non-operative treatment	Intramedullary nail	Plate	Other PHFx fixation	None
**For which treatment option(s) should vascular events be documented?**	☑	☐	☐	☐	☐	☐

Several options may apply

Definition : Events involving laceration, avulsion, contusion, puncture or crush injury to an artery, vein or microvascularity at the surgical site

Specifications :
- Hematoma which requires evacuation by needle or surgery
- Superficial and deep thrombosis at the involved extremity
- Ischemia of the involved extremity which requires additional intervention

Observation period (timeline) : 30 days

Do you agree with this definition, specifications, terminology and timeline? ○ Yes ○ No

reset

Comment or alternative suggestion

Expand

<< Previous Page

Next Page >>

Save & Return Later

Consensus development of a core list of unfavorable events of proximal humerus fracture treatment (survey 1)

Postoperative / non-operative local events (10)

Surgical Site Infections (SSI)

Definition of terms and specifications adapted from the 2008 Centers for Disease Control and Prevention (CDC) definition*
- Superficial Incisional Surgical Site Infections: Infections involving only the skin and subcutaneous tissue of the incision
- Deep Surgical Site Infections (Incisional AND Organ/Space): Infections involving any part of the anatomy (e.g. fascia, muscle, organs and spaces) other than the skin and subcutaneous tissue of the incision

Specifications: early (< 3 months) / low grade (3-24 months) infections*

Period of observation: 24 months (note: despite 12 months according to CDC definition when implant in place)

** Horan TC, Andrus M, Dudeck MA, (2008) CDC/NHSN surveillance definition of health care-associated infection and criteria for specific types of infections in the acute care setting. Am J Infect Control, 36: 309-332*

Do you agree with this definition, specifications, terminology and timeline for all operative treatment options? ○ Yes ○ No reset

Should local infections also be documented in non-operative treatment? ◉ Yes ○ No reset

How could we define infection in this context?

Expand

Comment or alternative suggestion

Expand

<< Previous Page   Next Page >>

Save & Return Later

Consensus development of a core list of unfavorable events of proximal humerus fracture treatment (survey 1)

Postoperative / non-operative local events (11)

Superficial soft tissue events

	All	Non-operative treatment	Intramedullary nail	Plate	Other PHFx fixation	None
**For which treatment option(s) should superficial soft tissue events be documented?** Several options may apply	☑	☐	☐	☐	☐	☐

Definition : Events affecting the superficial soft tissues (i.e. skin and subcutaneous tissue) at and around the fracture site/wound that do not affect deep soft tissues (i.e. fascia, muscle, articular capsule) and that require additional treatment

Specifications and observation period (timeline) :
- Early events over 30 days: edema; emphysema; delayed wound healing; hypersensitivity reaction; skin necrosis; skin bulla; other
- Late events over 6 months: hypertrophic scar and keloid

Do you agree with this terminology, definition, specifications and timeline? ○ Yes ○ No

reset

Comment or alternative suggestion

Expand

<< Previous Page   Next Page >>

Save & Return Later

Consensus development of a core list of unfavorable events of proximal humerus fracture treatment (survey 1)

Postoperative / non-operative local events (12)

Deep soft tissue events (1)

	All	Non-operative treatment	Intramedullary nail	Plate	Other PHFx fixation	None
**For which treatment option(s) should deep soft tissue events be documented?**	☑	☐	☐	☐	☐	☐

Several options may apply

Definition : Events affecting the deep soft tissues (i.e. fascia, muscle, articular capsule), except infections

Specifications :
- affecting the subacromial space (impingement, adhesion,...)
- affecting the biceps
- affecting the capsule (shoulder stiffness, ...)
- affecting the rotator cuff : events affecting the anatomical and functional integrity of the rotator cuff including one of the following muscles and tendons: subscapularis, supraspinatus, infraspinatus, teres minor.
- affecting the deltoid

Do you agree with the revised terminology, definition, and specifications ? ○ Yes ○ No reset

Which timeline is suitable for documenting deep soft tissue events?

	6 months	12 months	24 months	lifelong until implant removal	other time period
**... in joint-preserving fracture fixation?**	○	○	○	○	◉

reset

Specify the other time period

	6 months	12 months	24 months	other time period
**... in non-operative treatment?**	○	○	○	◉

reset

Specify the other time period

Comment or alternative suggestion

Expand

<< Previous Page   Next Page >>

Save & Return Later

Resize font:

Consensus development of a core list of unfavorable events of proximal humerus fracture treatment (survey 1)

Many thanks!

Do you have any comment?

Expand

<< Previous Page

Submit

Save & Return Later
