# Supplementary material for: Core set of unfavorable events of proximal humerus fracture treatment defined by an international Delphi consensus process
Source: BMC Musculoskelet Disord. 2021 Nov 30;22:1002. doi: 10.1186/s12891-021-04887-1 (PMC8630858; doi:10.1186/s12891-021-04887-1)
Supplement: Supplementary file 3 — Additional file 3. PHF Core Event Set v1.0 - Delphi 02 survey screenshots. [file 12891_2021_4887_MOESM3_ESM.pdf]

AO TRAUMA

Supplementary file 3

Article title Core set of unfavorable events of proximal humerus fracture treatment defined by an international Delphi consensus process

Journal name BMC Musculoskeletal Disorders

Author names Audigé L, Brorson S, Durchholz H, Lambert S, Moro F, PHF CES Consensus Panel, Joeris A

Affiliation Schulthess Klinik, CH-8008 Zurich, Switzerland

E-mail address laurent.audige@kws.ch

PHF Core Event Set v1.0

Core list of unfavorable events of proximal humerus fracture (PHF)

Delphi 2 survey screenshots

Resize font: ⊞ | ⊟

Returning?

Consensus development of a core list of unfavorable events of proximal humerus fracture treatment (survey 2)

Dear Colleague,

Thank you for your participation in this Delphi consensus project.

Please make sure that you answer all questions and complete the survey. You may leave the survey and return to it where it was left at any time. In such case take note of the provided code to allow returning and completing the survey. If you lose this code, you may contact me.

Kind regards

Prof Dr. Laurent Audigé, PhD on behalf of the steering committee

We invited 327 surgeons experienced in treating proximal humerus fractures (PHF), of which 230 agreed to participate in this Delphi process (70%); they were 175 (75%) to respond to all questions.

In this second online survey we present you with the detailed results including numerous comments and suggestions from the first survey. Based on all responses a proposal was developed for you to further review and comment.

99% (217/219) of respondents agreed about our development framework for developing the unfavorable event core set.

Attachment: DelphiSurvey01-AOTrauma-PHFx-01-Results-Concept.pdf (0.13 MB)

Do you agree to participate in this second survey? ○ Yes ○ No

* must provide value

reset

Next Page >>

Save & Return Later

AO TRAUMA

Consensus development of a core list of unfavorable events of proximal humerus fracture treatment (survey 2)

Published Core Event Set in shoulder arthroplasty (SA)

We present below two important publications recently completed regarding shoulder arthroplasty (SA) in the context of a similar international consensus process with shoulder surgeons. It covers the documentation of core sets of adverse events in one, and of radiological monitoring parameters in the other.

Our steering committee recommends that any PHF treated by SA should be documented on the basis of this consensus work. The present survey therefore focuses only on fracture treatment whether by surgery or non-operative management.

Note: we invite you to review these papers and make any comments or suggestions you may find appropriate in the context of PHF treatment.

Audigé L., Schwyzer H.-K., SA CES Consensus Panel, Durchholz H. Core set of unfavorable events of shoulder arthroplasty: an international Delphi consensus process. Journal of Shoulder and Elbow Surgery (open access) doi: 10.1016/j.jse.2019.07.021

Attachment: Audigé-2019-JSES-Core set of unfavorable events of SA.pdf (0.25 MB)

Durchholz H., Salomonsson B., Moroder P., Lambert S., Page R., Audigé L. on behalf of the SA Monitoring Steering Group, Core set of radiological parameters for shoulder arthroplasty monitoring: criteria defined by an international Delphi consensus process, Journal of Bone and Joint Surgery Open Access, 2019. 4(4): p. e0025 doi: 10.2106/JBJS.OA.19.00025

Attachment: Durchholz-2019-JBJSOpenAccess-Core Set of Radiographic Parame.pdf (0.16 MB)

Do you agree about that recommendation? ○ Yes ○ No

reset

Comment or suggestion

Expand

<< Previous Page | Next Page >>

Save & Return Later

AO TRAUMA

Consensus development of a core list of unfavorable events of proximal humerus fracture treatment (survey 2)

Intraoperative events (1)

Intraoperative events

97% (211/218) agreed about the proposed distinction between intra- and postoperative events

97% (206/213) agreed about the proposed definitions and specifications of intra-operative events

Attachment: DelphiSurvey01-AOTrauma-PHFx-02-Results-IntraPostOP.pdf (0.04 MB)

The intraoperative period is defined as the time interval between skin incision and skin closure. When the fracture is reduced under anaethesia in the context of non-operative management, an equivalent **"fracture reduction" period** is considered as the time interval between the patient entered the operating room (OR) and the time the patient exited the OR.

Do you agree with this added definition? ○ Yes ○ No

reset

Comment or suggestion

Expand

<< Previous Page | Next Page >>

Save & Return Later

AO TRAUMA

Consensus development of a core list of unfavorable events of proximal humerus fracture treatment (survey 2)

Intraoperative local events (2)

Changes are proposed regarding intraoperative device and soft tissue events as shown in red in the text below: note that "screw/bolt perforation" is no longer considered because, once removed intraoperatively after the perforation, it has no consequence to the patient (no event) and no surgeon would ever report it if that occured occasionally.

Device events

Definition : Events affecting any component of the implanted device or material, or the instrumentation used for their implantation.

Specifications :
- Instrument problem (breakage, failure)
- Implant (breakage, malpositioning, separation)
- ~~Screw / bolt joint perforation~~
- Cementation problem (augmentation)

Soft tissue events

Definition : Events involving only the soft tissue at the treated shoulder

Specifications :
- Skin, muscle, tendon, joint capsule, ligament, labrum
- Blood vessels (bleeding) : bleeding at the surgical site that requires additional intervention or leads to a stop of the operation
- Nerves*: recognized damage of a neurological structure ~~which needs additional surgical intervention~~

* a standard list of potentially affected nerves will only be presented for postoperative neurological events

Do you agree with these changes ? ○ Yes ○ No

reset

Comment or suggestion

Expand

<< Previous Page | Next Page >>

Save & Return Later

Resize font:

AO TRAUMA

Consensus development of a core list of unfavorable events of proximal humerus fracture treatment (survey 2)

Postoperative / non-operative local events (1)

Local event groups

96% (190/197) agreed about the proposed local event groups

Attachment: DelphiSurvey01-AOTrauma-PHFx-04-Results-PostOPGroups.pdf (0.03 MB)

The following minor changes shown in red were made by the steering group:

1- Implant (device) events: Events affecting any ~~implanted~~ used device (e.g. nail, plate, prosthesis, external fixator) which are shown on adequate postoperative imaging (e.g. radiographs, ultrasound, CT) or affecting any external device (e.g. sling, orthosis) used to immobilize the fracture, which is associated with clinical symptoms.

Do you agree about these minor changes? ○ Yes ○ No

reset

Comment or suggestion for change

Expand

<< Previous Page   Next Page >>

Save & Return Later

Consensus development of a core list of unfavorable events of proximal humerus fracture treatment (survey 2)

Postoperative local events (2)

Surgical implant (device) events

94% (181/192) of respondents agreed about the proposed definition, specifications and terminology

Non-operative local device events

96% (185/192) of respondents agreed on the proposed definition and observation timeline.

Participants reported that the following events should be documented in the context of non-operative management:
- breakage / loosening of the device (sling or orthosis)
- malposition / malfit
- stiffness of joints
- peripheral neurological symptoms
- skin lesions
- allergy
- pressure sores
- pain
- Compartment Syndrome
- Deep Vein Thrombosis
- edema of the upper extremity.

Note : Device type and non-compliance were not considered "events" but part of the treatment itself.

Attachment: DelphiSurvey01-AOTrauma-PHFx-05-Results-PostOP-Implants.pdf (0.06 MB)

The following changes shown in red were made by the steering group:

Surgical implant (device) events

Timeline: the minimum suitable timeline for documenting postoperative implant events should be 12 months.

Non-operative local device events

Definition : Events (e.g. breakage, loosening) involving any external device (e.g. sling, orthesis) used to immobilize the arm to support the fracture, which is associated with local clinical symptoms (e.g. local reactions such as skin lesions).

Do you agree about these changes? ○ Yes ○ No

reset

Comment or suggestion

Expand

<< Previous Page

Next Page >>

Save & Return Later

AO TRAUMA

Consensus development of a core list of unfavorable events of proximal humerus fracture treatment (survey 2)

Postoperative / non-operative local events (3)

Osteochondral events (1)

The proposed list of events was supported by participants with between 64% and 97% agreement for all treatment options (except "New fracture (around the implant)" and "Screw / bolt cutout" only following surgical interventions).

Numerous definitions were proposed and reviewed.

No consensus emerged about the minimum timeline required for documenting osteochondral events.

Attachment: DelphiSurvey01-AOTrauma-PHFx-06-Results-PostOP-Osteochondral.pdf (0.15 MB)

Definition : Events affecting the osteochondral tissue of the proximal humerus, clavicula and/or scapula

Specification :
Surgical treatment only:
- New fracture (around the implant)
- Screw / bolt cutout*

All treatment interventions:
- Bone formation / resorption
- Tuberosity migration / resorption
- Head necrosis
- Delayed union / nonunion
- Loss of fracture reduction
- Other event(s)

Timeline : 24 months

* may be associated with loss of fracture reduction (e.g. head collapse) and/or head necrosis

Note : the event "Fracture malunion" was no longer considered by the steering committee considering that the term reflects negative or inadequate performance. In any treatment one could consider that PHFx are somehow "malreduced", and in non-operative management, one would expect some degree of "malunion". What is to be tolerated for each patient is not well defined and some guidelines (outside the scope of this survey) would be very useful.

Do you agree with this definition, specifications, timeline and terminology? ○ Yes ○ No reset

Comment or alternative suggestion

Expand

<< Previous Page Next Page >>

Save & Return Later

AO TRAUMA

Consensus development of a core list of unfavorable events of proximal humerus fracture treatment (survey 2)

Postoperative / non-operative local events (4)

Shoulder instability

80%-82% (137-139/172) of respondents agreed that shoulder instability should be documented for all treatment options

97% (140/145) of respondents agreed on the proposed definition, specifications and terminology

Attachment: DelphiSurvey01-AOTrauma-PHFx-07-Results-PostOP-Instability.pdf (0.04 MB)

The following changes shown in red were made by the steering group:

Definition of terms : symptomatic shoulder associated with loss of alignment of the articulating surface of the humeral ~~component~~ head with the ~~articulating~~ glenoid surface ~~of its joint partner~~

Specifications :
- Subluxation : non arm position-dependent eccentric misalignment with residual contact.
- Dislocation : non arm position-dependent complete loss of contact of the articulating surfaces.
- Dynamic instability : arm position-dependent loss of contact of the articulating surfaces apparent on physical examination and/or visible on functional radiographs (horizontal flexion/extension view in 90° of abduction and true AP view in 60° of abduction).

Timeline : 12 months

Do you agree with these changes and the proposed timeline ? ○ Yes ○ No reset

Comment or suggestion

Expand

<< Previous Page Next Page >>

Save & Return Later

Resize font:

Consensus development of a core list of unfavorable events of proximal humerus fracture treatment (survey 2)

Postoperative / non-operative local events (5)

Vascular events

88%-98% (153-169/173) of respondents agreed that this event group should be documented for all treatment options

96% (163/170) of respondents agreed on the proposed definition, specifications and terminology

Attachment: DelphiSurvey01-AOTrauma-PHFx-09-Results-PostOP-Vascular.pdf (0.03 MB)

The following changes shown in red were made by the steering group:

Definition : Events involving laceration, avulsion, contusion, puncture or crush injury to an artery or vein ~~or microvascularity~~ at the ~~surgical site~~ injured arm

Specifications :
- Hematoma which requires evacuation by needle or surgery
- Superficial and deep thrombosis at the involved extremity
- Ischemia of the involved extremity which requires additional intervention

Observation period (timeline) : 30 days

Do you agree with these changes? ○ Yes ○ No

reset

Comment or suggestion

Expand

<< Previous Page | Next Page >>

Save & Return Later

AO TRAUMA

Consensus development of a core list of unfavorable events of proximal humerus fracture treatment (survey 2)

Postoperative / non-operative local events (6)

Surgical Site Infections (SSI)

93% (160/172) of respondents agreed on the proposed definition, specifications, terminology and timeline for all (surgical) treatment options

Only 45% (78/172) of respondents agreed that local infections should also be documented in non-operative treatment

Attachment: DelphiSurvey01-AOTrauma-PHFx-10-Results-PostOP-Infection.pdf (0.04 MB)

Despite this high level of consensus agreement, the steering group suggested however that the recently published consensus on "fracture-related infection" should be adopted*.

"For the purposes of a definition (and data collection), it is important that surgeons define the presence of infection, not its extent, localization or classification."

*Metsemakers WJ, Morgenstern M, McNally MA, et al. Fracture-related infection: A consensus on definition from an international expert group. Injury. 2018;49(3):505-510. PMID: 28867644. doi: 10.1016/j.injury.2017.08.040

Attachment: Metsemakers-2018-Injury-Fracture-related infection_A consensus definition.pdf (0.55 MB)

Fracture-related Infections (FRI)

Definition of terms and specifications adopted from the 2018 FRI consensus definition (Metsemakers et al. 2018)

Period of observation: 24 months

Do you agree with this recommendation? ○ Yes ○ No

reset

Comment or suggestion

Expand

<< Previous Page Next Page >>

Save & Return Later

AO TRAUMA

Consensus development of a core list of unfavorable events of proximal humerus fracture treatment (survey 2)

Postoperative / non-operative local events (7)

Deep soft tissue events

78%-99% (134-171/172) of respondents agreed that this event group should be documented for all treatment options

99% (163/164) of respondents agreed on the proposed definition, specifications and terminology

Attachment: DelphiSurvey01-AOTrauma-PHFx-12-Results-PostOP-DeepSoftTissue.pdf (0.04 MB)

The following changes shown in red were made by the steering group for a more logical sequence of events, described from an anatomic organisation from the articular surface and synovial outwards:

Definition : Events affecting the deep soft tissues (i.e. fascia, muscle, articular capsule), except infections

Specifications :
- External muscular envelope: deltoid-pectoralis major
- Subacromio-deltoid-coracoid bursa (space)
- Rotator cuff muscle-tendon and biceps tendon
- Capsule-synovium

Observation period (timeline): 12 months

Do you agree with these changes? ○ Yes ○ No

reset

Comment or suggestion

Expand

<< Previous Page | Next Page >>

Save & Return Later

Consensus development of a core list of unfavorable events of proximal humerus fracture treatment (survey 2)

Postoperative / non-operative local events (8)

Peripheral neurological events

91%-98% (157-170/172) of respondents agreed that this event group should be documented for all treatment options

97% (165/170) of respondents agreed on the proposed definition, specifications and terminology

Attachment: DelphiSurvey01-AOTrauma-PHFx-08-Results-PostOP-Neurological.pdf (0.03 MB)

Superficial soft tissue events

79%-98% (137-170/173) of respondents agreed that this event group should be documented for all treatment options

98% (165/168) of respondents agreed on the proposed terminology, definition, specifications and timeline

Attachment: DelphiSurvey01-AOTrauma-PHFx-11-Results-PostOP-SupSoftTissue.pdf (0.02 MB)

No change was made by the steering group for these two event groups.

Comment or suggestion

Expand

<< Previous Page

Next Page >>

Save & Return Later

Resize font:

Consensus development of a core list of unfavorable events of proximal humerus fracture treatment (survey 2)

Many thanks!

Do you have any comment?

Expand

<< Previous Page

Submit

Save & Return Later
