# Supplementary material for: Core set of unfavorable events of proximal humerus fracture treatment defined by an international Delphi consensus process
Source: BMC Musculoskelet Disord. 2021 Nov 30;22:1002. doi: 10.1186/s12891-021-04887-1 (PMC8630858; doi:10.1186/s12891-021-04887-1)
Supplement: Supplementary file 4 — Additional file 4. PHF CES Consensus Panel (231 participants in alphabetical order). [file 12891_2021_4887_MOESM4_ESM.pdf]

AO TRAUMA

Supplementary file 4

**Article title**	Core set of unfavorable events of proximal humerus fracture treatment defined by an international Delphi consensus process
**Journal name**	BMC Musculoskeletal Disorders
**Author names**	Audigé L, Brorson S, Durchholz H, Lambert S, Moro F, PHF CES Consensus Panel, Joeris A
**Affiliation**	Schulthess Klinik, CH-8008 Zurich, Switzerland
**E-mail address**	laurent.audige@kws.ch

PHF Core Event Set v1.0

Core list of unfavorable events of proximal humerus fracture treatment (PHF)

PHF CES Consensus Panel (231 participants in alphabetical order):

Dr. Wael Abdelhadi, Nasr City Insurance Hospital, Cairo, Egypt

Prof Dr. Lars Adolfsson, Linköping University Hospital, Linköping, Sweden

Dr. Archit Agarwal, ESIC PGIMSR & Model hospital, Delhi, India

Dr. Héctor J Aguado, Hospital Clínico Universitario de Valladolid, Valladolid, Spain

Prof Dr. Syed Kamran Ahmed, The Indus Hospital, Karachi, Pakistan

Dr. Hashem Alqdhah, Jordan hospital, Amman, Jordan

Dr. Mohammed Alsaifi, TMGH -Sana'a, Yemen, Sana'a, Yemen

Mr Muhammed Alsherbeeny, King Abdullah Medical city/ Madinah Specialty Hospital, Madinah Munawara, Kingdom of Saudi Arabia

Dr. Saeed Althani, orthocure medical center, Dubai, United Arab Emirates

Dr. Miha Ambrožič, University Clinical center Ljubljana, Ljubljana, Slovenia

Dr. Ashish Anand, VaMC, Mississippi, United States

Dr. Kunal Aneja, Deen Dayal Upadhyay Hospital, Delhi, India

Prof Dr. Guido Antonini, San carlo borromeo, Milan, Italy

Dr. Scholz Armin, Hermann-Josef-Krankenhaus, Erkelenz, Germany

Dr. Carlos Augusto Arroyo Sanchez, Clinica Foscal Internacional, Floridablanca, Colombia

Dr. Hideaki Asai, Japanese Red Cross Musashino Hospital, Tokyo, Japan

Dr. Khalid saleem Aslam, Quaid e Azam international hospital, Islamabad, Pakistan

Prof Dr. Terry Axelrod, Sunnybrook Health Science Centre, University of Toronto, Toronto, Canada

Prof Dr. Reto Babst, Luzerner Kantonsspital, Luzern, Switzerland

Prof Dr. Christian Bahrs, BG Unfallklinik, Tübingen, Germany

Prof Dr. Asen Baltov, Emergency Hospital Pirogov /MATHEM/, Sofia, Bulgaria

Dr. Jonathan Barlow, Mayo Clinic, Rochester, United States

Dr. Alfonso Barnechea Rey, Hospital Rebagliati, Lima, Peru

Dr. Jaime Barrio, Hospital de Jove, Gijon, Spain

Dr. David Beigler, Illinois Bone and Joint Intitute, Glenview, United States

PD Dr. Emanuel Benninger, Kantonsspital Winterthur, Winterthur, Switzerland

Dr. Antonio Berizzi, Azienda Ospedaliera Università di Padova, Padova, Italy

Dr. Serhii Bezruchenko, SI 'ITO NAMN of Ukraine', Kyiv, Ukraine

Dr. Ashutosh Bhosale, Morya Hospital & Research Centre, Satara, India

Dr. Craig Blake, Univeritas Hospital, Bloemfontein, South Africa

Dr. Inese Breide, Hospital of traumatology and orthopaedics, Riga, Latvia

Dr. Crist Brett, University Hospital--University of Missouri, Columbia, United States

Dr. Stig Brorson, Zealand University Hospital, Denmark

Prof Dr. Richard Buckley, Foothills Medical Center, Calgary, Canada

Dr. Preecha Bunchongcharoenlert, Pranungklao hospital, Nonthaburi, Thailand

Dr. Rastislav Burda, Faculty Hospital of L.Pasteur, Košice, Slovakia

Dr. Adler Burkhart, Krankenhaus der Augustinerinnen, Cologne, Germany

Dr. Carlo Busatto, Ospedale Santa Maria delle croci, Ravenna, Italia, Ravenna, Italy

Dr. Pedro Caba, 12 de octubre, Madrid, Spain

Dr. Marcio Calabria, HUGOL, Goiania, Brasil

Dr. Pedro Camaro, Hospital Universitario de Santander, Bucaramanga, Colombia

Dr. Eben Carroll, Wake Forest University, Winston Salem, United States

Mr Pablo Cañete, hospital de Manises, manises, Spain

Dr. Jian- Chih Chen, Kaohsiung Medical University Hospital, Kaohsiung, Taiwan

Dr. Ram Chidambaram, MIOT International Hospital, Chennai, India

Dr. federico Chiodini, legnano hospital, legnano, Italy

Dr. Ivan Chua, Tan Tock Seng Hospital, Singapore

Mr Dave Cloke, Northumbria Healthcare NHS Trust, Newcastle upon Tyne, United Kingdom

Dr. Francisco Collado Torres, Hospital Regional de Málaga, Malaga, Spain

Dr. Julio Contreras, Instituto Traumatologico de Santiago, Santiago, Chile

Dr. Vitor Correa, Hospital Padre Albino, Catanduva, Brasil

Dr. Endre Csonka, University of Szeged, Szeged, Hungary

Prof Dr. Bhavik Dalal, AMC MET Medical College, Ahmedabad, India

Dr. Stephen Davis, Hartford Hospital, Hartford, United States

Dr. Pietro De Biase, Azienda Ospedaliero Universitaria Careggi, Firenze, Italy

Dr. Mohammad Hossein Dehghani Tafti, Jesus Hospital of Isfahan, Isfahan, Iran

Dr. Holger Durchholz, Klinik Gut AG, Switzerland

Dr. Deepak Kumar Dutta, National Academy of medical Sciences,Bir Hospital Trauma Centre, Kathmandu, Nepal

Dr. Manish Dwivedi, All India Institute Of Medical Sciences (AIIMS) Bhopal, Bhopal, India

Dr. Rami El Abyad, Hotel Dieu De France Hospital, Beirut, Lebanon

Prof Dr. Khaled Emara, ain shams univ hospitals, Cairo, Egypt

Dr. Nathan Endres, University of Vermont, Burlington, United States

Dr. Christian Fang, Queen Mary Hospital, Hong Kong, Hong Kong

Dr. Cesar Fernandez, Hospital regional docente de Trujillo, Trujillo, Peru

Dr. Adriano Fernando Medes Junior, University Hospital of Universidade Federal de Juiz de Fora, Juiz de Fora / Minas Gerais, Brazil

Dr. Michael Gardner, Stanford University School of Medicine, Redwood City, United States

Dr. Mario Garuz, Hospital Santo Tomas, Panama, Panama

Prof Dr. Stefano Ghera, San pietro hospital, Roma, Italy

Dr. Tomasz Gieroba, SPSK 4, Lublin, Poland

Mr Simon Jeremy Gregg-Smith, Royal United Hospital,Bath NHS Trust, United Kingdom

Mr Philip Grieve, Blackrock Clinic, Dublin, Ireland

Prof Dr. Thomas Gross, Cantonal Hospital Aarau, Aarau, Switzerland

Prof Dr. Lars Grossterlinden, Asklepios Clinic Altona, Hamburg, Germany

Dr. Luke Harmer, Quincy Nedical Group, Quincy, United States

Dr. Langdon Hartsock, MUSC, Charleston, SC, United States

PD Dr. Clemens Hengg, Univ.-Klinik Innsbruck, Innsbruck, Austria

Mr Andrew Henry, Salford Royal Hospital, Salford, United Kingdom

Prof Dr. Pierre Hepp, University of Leipzig, Leipzig, Germany

Dr. Frima Herman, Kantonsspital Graubünden, Chur, Switzerland

Dr. Mumtaz Hussain, The children's Lahore, Pakistan, Lahore, Pakistan

Dr. Khaled Hussein, Khaled Hussein, Tripoli, Lebanon

Dr. John Itamura Itamura, Kerlan jobe institute, Los Angeles, United States

Prof Dr. Z. Ugur Işıklar, İstanbul Memorial Hospital, İstanbul, Turkey

Dr. Peter Jacko, L.Dérer Hospital, University Hospital Bratislava, Bratislava, Slovakia

PD Dr. Hendrik Jansen, University of Wuerzburg, Wuerzburg, Germany

Dr. Viswanath Jayasankar, Sundaram Medical Foundation, Chennai, India

Dr. Marcis Jegers, Hospital of traumatology and orthopedics, Riga, Latvia

Dr. Petr Jemelik, University Hospital Waterford, Waterford, Ireland

PD Dr. Alberto Jorge-Mora, Hospital Clínico de Santiago, Santiago de Compostela, Spain

Dr. Csotye János, Békés Counthy Hospital, Gyula, Hungary

Dr. Alexander Kaban, Kaplan Medical Center, Rehovot, Israel

PD Dr. Koroush Kabir, University hospital Bonn, Bonn, Germany

Dr. Amol Kadu, Upcharya hospital, Nagpur, India

Mr Niel Kang, Cambridge University Hospitals NHS Trust, Cambridge, United Kingdom

Dr. Takashi Kashiwa, Japanese Red Cross Urakawa Hospital, Urakawa-Town, Japan

PD Dr. Yukio Kawakami, Okayama Saiseikai General Hospital, Okayama, Japan

Prof Dr. Richard Kdolsky, Medizinische Universität Wien, Vienna, Austria

Dr. Nitin Kimmatkar, Government Medical College, Nagpur, Nagpur, India

Prof Dr. Bernd Kinner, Robert-Bosch-Krankenhaus, Stuttgart, Germany

Dr. Shimpei Kitada, Hyogo Prefectural Nishinomiya Hospital, Nishinomiya, Japan

Dr. Martin Kloub, Hospital Ceske Budejovice, Ceske Budejovice, Czech Republic

Dr. Dominic Konadu-Yeboah, Komfo Anokye Teaching Hospital, Kumasi, Ghana

Prof Dr. Gerhard Konrad, Klinikum Erding, Erding, Germany

Dr. Ladislav Kovacic, University Medical Centre Ljubljana, Ljubljana, Slovenia

Dr. Michael Krasheninnikoff, Nykøbing Falster Sygehus, Nykøbing Falster, Denmark

Dr. James Krieg, Rothman Institute, Philadelphia, PA, United States

Dr. Martin Krivohlavek, Regional Hospital Liberec, Liberec, Czech Republic

Dr. Akhil Kulshreshtha, Yashoda Superspeciality Hospital, Ghaziabad, India

Prof Dr. Vikas Kulshrestha, Armed forces India, Chandigarh, India

Dr. Gaurav Kumar, Integral Institute of Medical Sciences, Lucknow, India

Dr. Ritabh Kumar, Indian Spinal Injuries Cntre, New Delhi, India

Dr. Nirmal Kumar, Gandhi Nagar Hospital, Ranchi, India

Dr. Ashok Gavaskar Kumaresan, Dr. Rela Institute and Medical Centre, Chennai, India

Dr. Simon Lambert, University College London Hospital, London, United Kingdom

Dr. Shahid Latheef, NS Memorial Institute of medical sciences, Kollam, India

Dr. Michiel Leikmem, Alrijne Hospital, Leiderdorp, The Netherlands

Dr. Rodrigo Liendo, Hospital Clinico UC, Santiago, Chile

Dr. Michael Linn, Southside hospital, Bay shore, United States

Dr. François Loubignac, Sainte Musse Hospital, Toulon, France

Dr. Christian Lozano, Clinica Anglo Americana, Lima, Peru

Dr. Tal Luria, Beilinson, Petach tikva, Israel

Dr. Manuel Jose Malaret Baldo, Hospital Universitario de Caracas, Caracas, Venezuela

Dr. Ivan Marintschev, University Hospital Jena, Jena, Germany

Mr Damian McClelland, University Hospital of North Staffordshire, Stoke on Trent, United Kingdom

Dr. Hany Metwally, Police hospital cairo, Cairo, Egypt

Dr. Anna Miller, Washington University in St. Louis, Barnes Jewish Hospital, St. Louis, United States

Dr. Istvan Mitro, Hospital of LPasteur, Kosice, Slovakia

Dr. Samarth Mittal, JPNATC, AIIMS, New Delhi, India

Dr. Mattia Mocchi, Humanitas University Hospital Milano, Milano, Italy

Dr. Lakshminaathan Mohan, Kauvery Hospital, Chennai, India

Prof Dr. Nirmal Chandra Mohapatra, Fakir Mohan Medical College & Hospital, Cuttack, India

Prof Dr. Ashraf Moharram, Cairo University Hospitals, Giza, Egypt

Dr. Charles Moon, Cedars Sinai, Los Angeles, United States

Dr. Srinivas Moparthi, Moparthi Srinivas, Krishna, India

Dr. Ricardo Morelli, hospital vera cruz, campjnas, Brazil

Dr. Fabrizio Moro, Schulthess Klinik, Zürich, Switzerland

Dr. Larsen Morten Schultz, Odense University Hospital, Odense C, Denmark

Dr. Satish Mutha, Hinduja healthcare, Mumbai, India

Dr. Sohail Muzammil, Combined Military Hospital, Multan Cantt, Pakistan

Dr. Josep Maria Muñoz Vives, - Changing Institution & will clarify in a month-, L'Aldosa de la Massana, Andorra

PD Dr. Hawi Nael, Medizinische Hochschule Hannover, Hannover, Germany

Dr. Luis Naquira, instituto colombiano del dolor, medellin, Colombia

Dr. Andrea Nardi, Ospedale Santa Maria Misericordia, Perugia, Italy

PD Dr. Valentin Neuhaus, University Hospital Zurich, Zurich, Switzerland

Prof Dr. Javier Nistal Rodriguez Nistal Rodriguez, Rio Hortega Hospital, Valladolid, Spain

Dr. Diana Noriego, Hospital Dr Josep Trueta, Girona, Spain

Dr. Brent Norris, St John Medical Ctr, Tulsa, United States

Dr. Manoj Pahukar, Wockhardt Superspecialty Hospital, Nagpur, India

Dr. Shailesh Pai, Tejasvini Hospital & SSIOT, Mangalore, India

Dr. Chakra Raj Pandey, Grande International Hospital, Kathmandu, Nepal

Dr. Mikhail Panin, People's friendship university of Russia, Moscow, Russia

Dr. Giacomo Papotto, Policlinico-Vittorio Emanuele, Catania, Italy

Dr. Yogesh R. Parikh, Parikh Orthopaedic Hospital, Ahmedabad, India

Mr Vipul Patel, Epsom and St Helier University Hospitals NHS Trust, Sutton, United Kingdom

AO TRAUMA

Dr. Marco Pato, Hospital Prof. Doutor Fernando Fonseca, Amadora, Portugal

Dr. Rodrigo Pesantez, Fundacion Santa Fe de Bogota, Bogota, Colombia

PD Dr. Christian Pfeifer, University Hospial Regensburg, Regensburg, Germany

Prof Dr. Marinis Pirpiris, Epworth Hospital, Richmond, Australia

Prof Dr. Chetan Pradhan, Sancheti Institute for Orthopaedics & Rehabilitation, Pune, India

Dr. Glen Purnomo, St. Vincentius a Paulo Catholic Hospital, Surabaya, Indonesia

Dr. Jose Eduardo Quintero, Clinica Fracturas y Fracturas, Pereira, Colombia

Dr. Marek Radziejowski, CMJAH and Life Springs Parkland Private Hospital, Springs, South Africa

Dr. Mark Reilly, University Hospital Rutgers-New Jersey Medical School, Newark, United States

Dr. Nikolaus Renner, Kantonsspital Aarau, Aarau, Switzerland

Dr. Falk Reuther, DRK Kliniken Berlin Koepenick, Berlin, Germany

Prof Dr. David Ring, Dell Medical School, Austin, United States

Dr. Ewan Ritchie, Alrijne Hospital, Leiderdorp, The Netherlands

Mr Nigel Rossiter, Basingstoke & North Hampshire Hospital, Basingstoke, United Kingdom

Prof Dr. Sami Roukoz, Dotel Dieu Hospital/Saint Joseph University, Beitut, Lebanon

Dr. Amarendra nath Roy, Murshidabad medical college, Berhampore, India

Prof Dr. Sigitas Ryliškis, Republican Vilnius University Hospital, Vilnius, Lithuania

Dr. Alaeddin Sabbah, Makassed Hospital, Jerusalem, Palestine

Prof Dr. Hatem Said, Assiut University Hospital, Assiut, Egypt

Dr. Amhaz Sámer, Chu santiago de compostela, Santiago de compostela, Spain

Dr. Tannous Jorge Sassine, Hospital Unimed Vitoria, Vitória, Brazil

Dr. Luca Saturno, Ospedale civile SS Annunziata, Sassari, Italy

PD Dr. Niels Schep, Maasstad Hospital Rotterdam, Rotterdam, Netherlands

Dr. Michael Schmelz, Kantonsspital Olten, Olten, Switzerland

Dr. Michael Schneider, St. Josephs Hospital, Wiesbaden, Germany

Prof Dr. Michael Schädel-Höpfner, Lukaskrankenhaus Neuss, Neuss, Germany

Mr Franz Josef Seibert, Medical University Graz - Austria, Graz, Austria

Dr. Daas Selim, CHU Nabeul, Nabeul, Tunisia

Mr Nasir Shah, Warrington Hospital, Warrington, United Kingdom

Prof Dr. Ram Kewal Shah, Janakpur Trauma Hospital, Janakpur, Nepal

Dr. Tushar Shah, Bhailal Amin General Hospital, Vadodara, India

Dr. Rosan Shah Kalawar, B. P. Koirala Institute of Health Sciences, Dhara, Nepal

Dr. Amit Kumar Sharma, Sundaram Medical Foundation, Chennai, India

Mr Sunil Sharma, NHS Fife, Kirkcaldy, United Kingdom

Dr. Vivek Shetty, P D Hinduja hospital Mahim, Mumbai, India

Dr. Shiv Manik Ajoy, Ramaiah Medical College and Hospitals, Bangalore, India

Dr. Anil Singhal, Traumacare &Arthroscopy centre, Bulandshahr, India

PD Dr. Alexandre Sitnik, Belarus Republic Scientific and Practical Center for Traumatology and Orthopedics, Minsk, Belarus

Dr. Malcolm Smith, Mgh, Boston, United States

Dr. Christoph Sommer, Kantonsspital Graubünden, Chur, Switzerland

PD Dr. Christian Spross, Kantonsspital St. Gallen, St. Gallen, Switzerland

Dr. Dimitrios Stafylakis, Hôpitaux Universitaires de Genève, Genève, Switzerland

Dr. Philipp Streubel, University of Nebraska Medical Center, Omaha, United States

Dr. Markus Stumpf, St. John of God Hospital Munich, München, Germany

Dr. Yu-Ping Su, Taipei Veterans General Hospital, Taipei, Taiwan

Prof Dr. Fabio Alfonso Suarez Romero, Hospital Militsr Central, Bogots, Colombia

Dr. Panchu Subramanian, Princess Marina Hospital, Gaborone, Botswana

Dr. Michael Swords, Sparrow Hospital/Michigan Orthopedic Center, Lansing, United States

Dr. Plácido Sánchez Gómez, Hospital vega Baja de Orihuela, San Bartolome, Orihuela, Spain

Dr. Michael Tanner, University Heidelberg, Heidelberg, Germany

Dr. Sachin Tapasvi, The Orthopaedic Speciality Clinic, PUNE, India

Dr. La Min Than Cho, Yangon orthopedics hospital, Yangon, Myanmar

Dr. Andreas Thannheimer, Klinikum Garmisch-Partenkirchen, Garmisch-Partenkirchen, Germany

Dr. Jan Theopold, Universitätsklinikum Leipzig, Leipzig, Germany

Dr. Taschner Tibor, County hospital Rottweil, Rottweil, Germany

PD Dr. Klatte Till Orla, University Hospital Hamburg-Eppendorf, Hamburg, Germany

Dr. Frederick Tonnos, Detroit medical center, Detroit, United States

Dr. Fjalestad Tore, Oslo University Hospital, Oslo, Norway

Dr. J Andrew I Trenholm, Halifax Infirmary, Halifax, Canada

Dr. Andrey Tulchinskiy, Tosnenskaja KIH, Tosno, Russia

Dr. Mirko Velickovic, AMEOS Klinikum Aschersleben, Aschersleben, Germany

Dr. Jan Verbruggen, Maastricht University Medical Center, Maastricht, Netherlands

Dr. Giovanni Vicenti, Policlinico Hospital, Bari, Italy

Dr. Marin Vidošević, General hospital Šibenik, Šibenik, Croatia

Dr. Gurunath Wachche, Government Hospital Solapur, India, Solapur, India

Dr. Naman Wahal, All India Institute of Medical Sciences (AIIMS), New Delhi, India

Dr. Shah Waliullah, King George's Medical University Lucknow, Lucknow, India

Dr. Markus Wambacher, Medical University Innsbruck, Innsbruck, Austria

Dr. Yoram Weil, Hadassah Medical Center, Jerusalem, Israel

Mr John Williams, Newcastle upon Tyne Hospitals, Newcastle, United Kingdom

Miss Lora Young, West Suffolk Hospital, Bury St Edmunds, United Kingdom

Dr. Christiam Zegarra Rodriguez, Peruvian Air Force Hospital, Lima, Peru

Dr. Carlos Miguel Zublin Guerra, Hospital Policia Federal Argentina:, Buenos Aires, Argentina

Dr. Zhenbang lv, Beijing Huaxin hospital, Beijing, China

Dr. Guy Putzeys, AZ Groeninge, Kortrijk, Belgium

Dr. Mark van Dijk, AZ Delta, Roeselare, Belgium

PD Dr. Dragan Đurđević, University Trauma Hospital UCC Sisters of Mercy Zagreb Croatia, Zagreb, Croatia
