# Supplementary material for: Core set of unfavorable events of proximal humerus fracture treatment defined by an international Delphi consensus process
Source: BMC Musculoskelet Disord. 2021 Nov 30;22:1002. doi: 10.1186/s12891-021-04887-1 (PMC8630858; doi:10.1186/s12891-021-04887-1)
Supplement: Supplementary file 5 — Additional file 5. PHF Core Event Set v1.0 - Delphi 01 survey results – July 2019. [file 12891_2021_4887_MOESM5_ESM.pdf]

AO TRAUMA

Supplementary file 4

**Article title**	Core set of unfavorable events of proximal humerus fracture (PHF) treatment defined by an international Delphi consensus process
**Journal name**	BMC Musculoskeletal Disorders
**Author names**	Audigé L., Brorson S., Durchholz H., Lambert S., Moro F.,<br>PHF CES Consensus Panel, Joeris A
**Affiliation**	Schulthess Klinik, CH-8008 Zurich, Switzerland
**E-mail address**	laurent.audige@kws.ch

PHF Core Event Set v1.0

Core list of unfavorable events of proximal humerus fracture treatment (PHF)

Delphi 01 survey results

July 2019

AO TRAUMA

Conceptual development of the core set

This core event set development is based on a **conceptual framework** from previous experiences with similar projects as well as new considerations resulting from a literature review of unfavorable events of proximal humerus fracture (PHF) treatment. Note that we will avoid the term "complication" in this survey, because it remains undefined.

In the field of PHF, we are considering operative as well as non-operative treatment options. Unfavorable events therefore may be common to all PHF or specific following one or more treatment options. Treatment options include joint-preserving fracture fixation (mostly by plating or intramedullary nailing) and joint replacement by arthroplasty. A consensus was recently achieved regarding a core set of events in shoulder arthroplasty (SA), therefore we plan to address the detailed event specifications in SA in a subsequent survey.

We are focusing on **local (regional) unfavorable events** affecting the treated shoulder, which are considered in a hierarchical structure involving event groups and specifications.

In the context of operative treatment, we distinguish further **intraoperative** and **postoperative** events.

Nonlocal events affecting the rest of the body are addressed globally for all orthopedic interventions because they are not specific to PHF.

The proposed structural documentation is purely descriptive without interring on any influencing factor(s) for the event(s). In addition the events themselves are distinguished from their treatment (e.g. a re-operation or revision operation) and outcome (e.g. disability or death). Unfavorable events in the core set may not include all adverse events as defined by regulation; also they may not be all considered treatment complications.

Audige et al. How to document and report orthopedic complications in clinical studies? A proposal for standardization. Arch Orthop Trauma Surg 2014, 134(2): 269-275

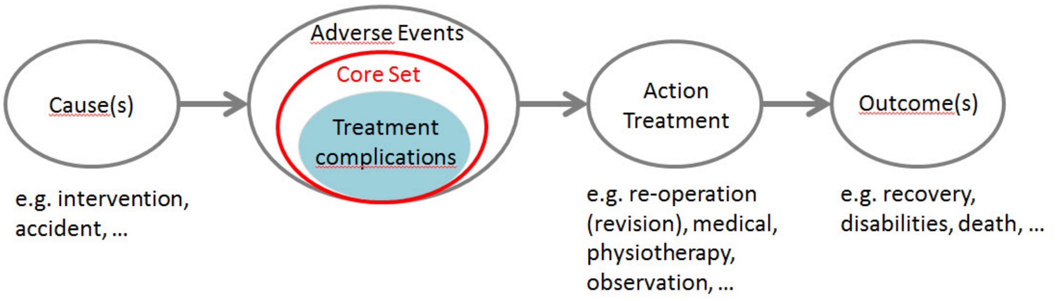

99% (217/219) agreed about this development framework for developing the unfavorable event core set

Comments and suggestions from participants who disagreed

"Not sure things are so simple"

"my suggestion is to put a separate circle for treatment option and treatment condition as step 2 where we can discuss the fracture pattern and bone status and treatment options and to discuss some prospective and prognostic values of treatment after that we can put the adverse events and core set"

Comments and suggestions from participants who agreed

"The flow pattern seems fine and at this juncture we would like follow cases whether treated conservatively or surgically, their treatment outcome and evaluate with the best possible evidence based approach."

"A consensus will be interesting, but - there is good evidence that what we as surgeons believe is not often backed up by evidence from good RCTs. We should not abandon evidence from well conducted RCTs even if they go against our preconceptions."

"I agree with the process, however I suggest to implement a timeline e.g. early vs. late vs. chronic Adverse Events."

"The differentiation between TREATMENT COMPLICATION and ADVERSE EVENTS is very useful. I had last week a central PAE due to an adverse Event in a 145 kg Patient - a sad Thing !!!"

"Factors Aggravating or Predisposing unfavorable events should also be given space"

"Is this approach similar to the approach utilized by Paley for external fixation related issues - where he viewed adverse events as either problems, obstacles or complications ? He had published in this."

"It is great that non-operative treatment option is being considered as well"

Intraoperative versus postoperative events

An **intraoperative event** is any event that occurs or is recognized during the time interval between skin incision and skin closure*.

A **postoperative event** is any event that occurs or is recognized during the time interval between the date and time (to the nearest minute) that the patient exited the operation room and the end of the observation period**.

adapted by
Audige L, Flury M, Muller AM, ARCR CES Consensus Panel, Durchholz H. Complications associated with arthroscopic rotator cuff tear repair: definition of a core event set by Delphi consensus process. J Shoulder Elbow Surg 2016;25:1907–1917.

from
Jacobs JP et al. (2007). What is operative morbidity? Defining complications in a surgical registry database. The Annals of Thoracic Surgery 84(4): 1416-1421
Rosenthal et al (2015) Definition and Classification of Intraoperative Complications (CLASSIC): Delphi Study and Pilot Evaluation. World Journal of Surgery 39: 1663-1671

*All events occurring between the date and time that the patient entered the operating room (OR) and the date and time when the patient exited the OR should be considered intraoperative events according to Jacobs JP et al, yet relevant intraoperative events in the core set would occur during the surgical procedure.

** The end of the observation period is defined for each event or group of events in the core set

97% (211/218) agreed about this distinction between intra- and postoperative events

Comments and suggestions from participants who disagreed

"What about time between skin closure and time of leaving the OP-room?"

"What about an event determined during intervention but recognized at postop xrays (we usually perform postop xrays the day after surgery)"

"E.g. Bleeding may be missed and is only recognized in the recovery room or even afterwards, but is a mishap in the surgery"

"Intra-operative event occurs as suggested above, anytime between skin incision and closure. Postoperative event is any event occurring after skin closure (which is the defined operative end point, noted above)."

"Where do events in recovery room Or in hospital fall"

Comments and suggestions from participants who agreed

"Postoperative event can be in the acute or subacute period, This time frame is arbitrary but often seen as the first 2-3 weeks post (acute). "

"Hmm, some intraoperative events are not recognized during the procedure itself, however are detected after the patient left the theatre (e.g. burns due to electrode issues)"

"I presume this means that an error or problem that occurred during an operation, but was not recognized until afterwards (i.e. on an x-ray post operatively) counts as an intraoperative event"

"How long is the postoperative event defined ? E.g. postoperative vs. chronic?"

"'An event that is recognized during the time interval between skin incision and skin closure' is a very relative term. Maybe skin incision should be modified, because a manipulation previous to skin incision may generate a preoperative complication and is not included in the term."

"so my PAE was a postoperative adverse Event ???"

"Concerning the definition of 'Postoperative event': If patients in one trial will have a follow-up of 3 months while in another 2 years, the report of 'Postoperative events' could not be compared. Thus to me the definition of 'postoperative event' is a bit odd, and would better be defined as a given period of time e.g. 6 months "

"There should be some labelling of the period immediately after the end of closure of skin and the patient exiting the operation room. There are few complications especially iatrogenic which may occur at that time. Transferring the patient form or table to wheel out stretcher is one potential time and place for unfavorable events. Maybe something like 'juxta operative' period....just a suggestion."

"The time interval between skin closure and leaving the operating room is a little controversial however at a system level it is probably best kept with the intraoperative period."

"'Intraoperative & postoperative 'DISCOVERY' is a more accurate than 'event'"

"may I add technical event which is the time since you reach the site of fracture till finishing operative fixation."

"If we take the view of Jacob et al then anesthesia complications during induction & reversal may add to the unfavorable events. so complications from skin incision & skin closure should be included in intraoperative event."

"Why not adapt the definition of intra operative event as -------between the time patient enters the OR till the patient exits the OR."

AO TRAUMA

Intraoperative local events

Device events

Definition : Events affecting any component of the implanted device or material, or the instrumentation used for their implantation.

Specifications :
- Instrument problem (breakage, failure)
- Implant (breakage, malpositioning, separation)
- Screw / bolt joint perforation
- Cementation problems

Osteochondral events

Definition : Events affecting the osteochondral tissue of the proximal humerus, clavicula and/or scapula

Specifications : Fracture (including hairline fracture), articular cartilage damage

Soft tissue events

Definition : Events involving only the soft tissue at the treated shoulder

Specifications :
- Skin, muscle, tendon, joint capsule, ligament, labrum
- Blood vessels (bleeding) : bleeding at the surgical site that requires additional intervention or leads to a stop of the operation
- Nerves*: recognized damage of a neurological structure which needs additional surgical intervention

*a standard list of potentially affected nerves will only be presented for postoperative neurological events

97% (206/213) agreed about these definitions and specifications of intra-operative events

Comments and suggestions from participants who disagreed

"Blood vessels should be termed arterial injury."
"Nerve injury may not be immediately recognized and may not need additional surgery"
"Neurological damage is unclear. A temporary palsy because of manipulation without full recovery but not suitable for surgical intervention is not a local event? Why surgical intervention is the criteria for including neurological damage when we should not define the complication according to it treatment? Maybe any neural damage should be included. In a similar way bleeding should be modified."
"- Instrument or implant problems should be specified whether due to material or handling problem or surgical error - joint perforation in my opinion is not an implant problem but a surgical error"
"Environmental events pertaining to theater environment may include but not be limited to: 1. Anaesthesia related complications. 2. Ancillary equipment failure like table, diathermy, C arm, laminar flow, power breakdown etc. "
"I'm not sure, if the term screw/bolt joint perforation in device events is in any time an event. Some surgeons drill through the articular surface to get the best stability of the screw and make it as long as possible. Anyway, this Drilling causes an articular cartilage damage (osteochondral event) but could be absolutely normal. Even if you use some k-wires to stabilize the fracture you can make an cartilage damage. Soft tissue is ok."
"recognized damage of a neurological structure should be the event… whether or not it needs additional surgical intervention "

AO TRAUMA

Comments and suggestions from participants who agreed

"Drug/anaesthetic events: - nerve injury following regional blockade - antibiotic reaction/anaphylaxis"
"Screw or bolt articular perforation should be included in the osteochondral events in my opinion. "
"Soft tissue events - nerves - If the definition includes 'which needs additional surgical intervention then its relying on a treatment decision by the surgeon. Shouldn't the definition of the event be independent of the treatment?"
"Intraoperative events arising as a result of patient positioning as shoulder tables are not routinely available in our setups e.g. Brachial plexus traction injury as a result of excessive tilt of the cervical spine"
"Breakage of an implant very unlikely intraoperative. Instrumentation? Seldom possible. 'Hairline fracture' ?"
"specification device problem: choosing the wrong implant (non-original, bad qualit ect.)"
"does this include infection?"
"Maybe to add missing of adequate material !"
"can we add malreduction of fractures & re-displacement during fixation as intraoperative unfavorable events."

Postoperative / non-operative local events (1)

We propose to structure local events according to the following <u>event groups</u>:

1-Implant (device) events: Events affecting any implanted device (e.g. nail, plate, prosthesis) which are shown on adequate postoperative imaging (e.g. radiographs, ultrasound, CT) or affecting any external device (e.g. sling, orthesis) used to immobilize the fracture, which is associated with clinical symptoms

2- Osteochondral events: Events affecting the osteochondral tissue of the proximal humerus, clavicula and/or scapula

3- Shoulder instability: Symptomatic shoulder associated with loss of alignment of the articulating surfaces

4- Peripheral neurological events: Events resulting from peripheral neurological injury at the fracture site, which are associated with sensory and/or motor and/or autonomic disturbance

5- Vascular events: Events involving laceration, avulsion, contusion, puncture or crush injury to an artery, vein or micro vascularity at the fracture site

6- Infections: Surgical Site Infections (SSI) with definition and specifications adapted from the 2008 Centers for Disease Control and Prevention (CDC) definition AND, in shoulder arthroplasty, late hematogenous infections with periprosthetic infections defined according to international consensus

7- Superficial soft tissue events: Events affecting the superficial soft tissues (i.e. skin and subcutaneous tissue) at and around the fracture site/wound that do not affect deep soft tissues (i.e. fascia, muscle, articular capsule) and that require additional treatment

8- Deep soft tissue events: Events affecting the deep soft tissues (i.e. fascia, muscle, articular capsule), except infections

Notes

None of the considered events in the core set must be present or occur prior to or at the time of trauma. Hence they are to be distinguished from concomitant lesions directly resulting from the trauma.

Some of these event groups may not apply to specific treatment modalities. This will be determined in the following sections of this survey.

AO TRAUMA

Participants' agreement with these event groupings (n,%)

Definitely not	-
Rather not	3 (2%)
Undecided	4 (2%)
Rather yes	75 (38%)
Yes, definitely	115 (58%)

Comments and suggestions

"Rates of venous thromboembolic events particularly pulmonary emboli, to guide the discussion on need and type of any thromboprophylaxis"

"Nerve injuries should be included in one of the groups"

"-Periarticular calcification where does it belong? -Head necrosis"

"Not sure about microvasculature damage defined as 'vascular' event, rather should be part of 'soft tissue' events"

"'Patient related events' e.g. multimorbidity, alcohol abuse, osteoporosis, obesity, neurological problems"

"Because the Saphenous vein and its branches were exposed in the operative field and easily got injured, is this a vascular event? How to define the microvascularity at the fracture site?"

"But the rotator cuff needs to be emphasized as an entity"

"Would be very difficult to decide about the 'depth' of the infection."

"Where are Malreductions and Non unions?   What about shoulder Stiffness? is that under 8?"

"I think that the grouping is very good"

"Minor vein or micro vascular events irrelevant "

"I do not know whether you should include external devices under implants"

"I will probably include FRI criteria (Injury 2018) in infection."

"Still in doubt about the definition. intraop complication is something that occurred during intervention even is recognised on the postop xrays (an intrarticular screw not seen intraop)"

"we Need a clear diagnosistic or a least a description of the Trauma of the shoulder during surgery"

"I like this!!!!"

"Implant Events that depend on clinical symptoms are difficult. A Perforation of the head without complaints. is this a complication or not?"

"If we were to change shoulder instability to joint instability, then we may be able to apply this to other joints as well."

"May need to add ultrasound to assess rotator cuff and biceps"

"Waaaaay too complex. While theoretically sound, of no use in daily practice"

"ROM should be include the event groups."

"2 exclude clavicula"

"In deep soft tissue events consider stiffness as an example."

"In surgical treatment, some may be intraoperative events, it is difficult to identify when the event occurred: - Osteochondral events - Shoulder instability   - Peripheral neurological events - Vascular events"

"I am not sure about osteochondral events , probably most of them can be classified as implant related "

"I would add events related to the rotator cuff and biceps tendon"

"None "

"I think there are maybe to many"

"Preexisting rotator cuff tears discovered during the surgical intervention should be included as a separate group since it is not unusual to encounter this pathology during fixation"

"Some are very rare (instability, vascular, neurological)   consider the use of periop. regional anaesthesia (may have biasing nerve affection)"

Postoperative local events: Implant (device) events

Definition : Events affecting any implanted device (e.g. nail, plate) which are shown on adequate postoperative imaging (e.g. radiographs, ultrasound, CT), which is associated with clinical symptoms

Specifications :

- Malpositioning: implant not in its expected position
- Radiolucency around the implant / Implant loosening
- Screw or ~~bold~~ bolt backout
- Implant breakage
- Implant migration (subsidence, tilt, shift): change of the position of a component, relative to the bone it supposedly fixed to

Note: A malpositioned implant may result from intraoperative malpositioning and/or postoperative implant displacement. The time of occurrence may be determined by immediate postoperative assessment of the implant position

94% (181/192) of respondents agreed about with this definition, specifications and terminology

Suitable timeline for documenting postoperative implant events	n (%)
3 months	47 (24%)
6 months	50 (26%)
12 months	57 (29%)
24 months	15 (8%)
Lifelong until implant removal	18 (9%)
Other period*	8 (4%)

* Suggested periods were specified as: “Until union has been achieved”; “Until healing or failure”; “Postop, 6 weeks, 3 months, 6 months, 12 months”; “Until healing”; “6 weeks”; “18 months” and “Dependent on type of implant and progress of consolidation” (one response was missing).

Comments and suggestions

"Immediate post op. xrays and after 3 weeks"

"In case of no union more"

"In case of fractures, if fracture heals, one year is enough"

"If any of such implants failure happens, it will happen within 3 months in my experience. But sometimes it may happen after 6 months if these patients perform overloading before consolidation of fracture site."

"Definition of pseudarthrosis"

"May need another time to confirm HW is ok at say 6 months also."

"Regarding 'malpositioning' the term of 'expected position' seems rather subjective. I suggest to change into 'suggested' or 'required'."

"Events should be divided as early and late. Early would be for example, proud positioning of the implant and late implant breakage."

"Screw cut out or penetration of articular surface"

"I believe that the control should be at 3, 6 months until the year, since in many cases the events in relation to the implant develop late."

"What is screw or 'bold' backout?"

"Implant removal which may or may not happen. Albeit most likely the effective timeline of implant complications is probably at whatever point nonunion is declared"

"I suggest 5 postoperative follow ups in order to also determine between intra-/perioperative implant events and long term related implant events"

"From Post Op, till fracture healing."

"Implant related events are not limited to the time but to the presence of this material in my opinion"

"Assuming you mean screw or 'bolt' backout?"

"Malpositioning should be limited to intra-op,   postop displacement is a separate event implant bending What about fracture fragment migration relative to the implant At what level ? humeral head or humeral diaphysis "

"We can expected complications until the fracture is healed. Afterwards, the majority of the complications are related to implant removal, that should be addressed separately."

"Three months is suitable since after that there is gradual increase in the lost to follow up rate and hence it may not depict the actual incidence of complications and their relevance. Otherwise 12 months follow up seems more comprehensive."

"12 months without radiological changes"

"Until bony consolidation or implant removal"

"I think 12 months is enough to see any implant event!!!"

"Again, the discussion will be about 'with clinical symptoms'. Personally I agree, if no clinical symptoms than no complication. However, this is a Major discussion point."

"Intra- and immediate postoperative imaging should be separated from follow-up controls as to better differentiate surgical work from secondary effects (such as non- healing, implant related etc) timeline: at least!"

"6 weeks 3 , 6 months, 12 months optional "

"Reasoning should be given space for proper evaluation and also for measures taken for the events"

"3 month and 12 month"

"Actually, the work of implant is only till the bone unites, but it can attract few unfavorable events till it is in-situ."

"Over a twelve month period with multiple follow up"

"As far as the fracture is evolving to healing , in fractures at the extremities of long bones you have a stable mechanical condition at 3 months. otherwise if have a delayed union or epiphyseal necrosis you will need a longer image follow up"

"If fracture subsidence occurs and articular perforation then occurs is it the device or healing process??"

"The issue is that the events occur at different timelines malposition - immediate radiolucency - 6-12 months screw blackout - 3-6 months implant breakage - 12-18 months implant migration - variable"

"Consider secondary screw penetration (mixed osteochondral event and implant event) in ORIF"

"Screw perforations sometimes take place following the avascular necrosis of the humeral head.   How should we describe them as complications? 1. avascular necrosis only 2. screw perforations and avascular necrosis,; separately described. 3. screw perforations after avascular necrosis; consecutively described."

"Prostheses need shorter follow-up than osteosynthetic implants as no need to monitor osteoregeneration as diligently. Likewise, osteoporotic fractures will need longer f/u than young patients. Elective implant removal has very poor scientific basis, so should be omitted as a timeframe"

"We can add also screw non locking in the plate of LCP , It can happen in the uni directional locking head screws if missed the correct direction specially in the osteoporotic bone"

"6 months or till healing of the fracture"

"'Implant Migration' is misleading. Implant rarely migrates, the bone fails around the unchanged implant. I would change the wording."

"3 and 12 months"

"Perhaps you should add the pull-out failure mechanism of the plate/screws as specification. "

"While six months is likely more than enough time for most patients, it should capture the vast majority of complications."

"Please add screw penetration into the joint"

"In vast majority of cases, implant related issues are evident within the first 6-8 weeks."

"Also include implant usage with improper principles"

"Late subsidence and screw penetration can occur especially in valgus impacted and malreduced fractures or when soft tissues are compromised during the surgical intervention"

"In some instances implant position may not change but bone segment may escape (e.g. greater tuberosity). This should be specified. "

"Intraoperative malpositioning and postoperative implant displacement are 2 separate things and both should be included independently"

"Malposition: good Screw cut out: must be considered (is one of the most common complications of locking plates which is the most commonly used implant for These fractures) Screw backout: rare (maybe for nails) Loosening: rare maybe only used for arthroplasty but then >12mt fup Implant breakage: good Implant Migration: rare (rather consider cut out of screws)"

"At 2 weeks post op as well"

Non-operative local events (3): Device events

**Definition** : Events any external device (e.g. sling, orthesis) used to immobilize the fracture, which is associated with clinical symptoms<br>**Observation period** : Time during which the immobilization device(s) is(are) in use

96% (185/192) of respondents agreed about with this definition and observation timeline

Which specific device event(s) should be documented in the context of non-operative treatment?

Breakage, loosening	"breakage loosening compression"<br>“breakage”<br>“Breakage of the splint”<br>"Displacement of immobilization device”<br>“Damaged bandage”<br>“device breakage"
Malposition / malfit	“malposition unstable malfit impinging thumb or elbow strangulation"<br>"Bad position of orthesis which can cause dislocation.”<br>"Incorrect positioning, soft-tissue damage"<br>"Malpositioning of the sling / splint"<br>“Strangulation of the arm”<br>“material chaffing discomfort when wearing awkward adjustment“<br>"most important would be position of the arm and if there were soft tissue issues or neurapraxias or discomfort"<br>"Ease of wearing device Level of comfort Functional independence in performance of activities of daily living with brace on"
Stiffness	"Stiffness of elbow, external rotation stiffness"<br>"Elbow stiffness Hand stiffness "<br>"stiffness elbow"<br>"Decrease in the ROM "<br>“neighbouring joints (elbow) rigidity”<br>"Stiffness”<br>"neck stiffness and elbow stiffness needing physiotherapy"<br>“Joint stiffness”<br>“Stiffness of shoulder and other joints.”<br>"Joint stiffness"

Peripheral neurological symptoms	"radial nerve palsy due pressure at lower end of humerus"<br>"peripheral neurological symptoms due to nerve compression (e.g. ulnar nerve)"<br>“excessive pressure to the tissue with neurological consequences”<br>“Sympathetic Nervous changes like CRPS."<br>“peripheral neurological events”<br>“Nerves and edema compressions by orthesis”<br>“nerve injuries”<br>“Neurovascular events"<br>“neurapraxias”<br>"Ulnar nerve injury from sling use"<br>“ulnar nerve symptoms from sling”
Skin lesions	“Patient comfort with the device, skin irritation from the Orthosis etc. “<br>"Skin reaction"<br>"Sweating Maceration Skin lesions"<br>"Skin irritation"<br>"skin irritation"<br>“Skin irritation Decubitus"<br>“Skin ulcerations ?"<br>"Superficial soft tissue events"<br>"Skin lesions"<br>“Skin problems caused by the device”<br>“Bruises and other soft tissue complications caused by sling or orthesis"<br>"Skin Problems cervical spin problems"<br>"Skin irritation"<br>"skin irritations and lesions caused by device or member positioning"<br>“lacerations”<br>"Skin breakdown/rashes requiring ongoing care"<br>"Skin ulcer due to the sling compression"<br>“skin breakage”<br>“Skin breakdown”<br>"Skin problem"<br>"skin breakdown. skin fungal infection, skin bacterial infection"<br>"skin complications"<br>"skin problems"<br>"skin breakdown"<br>“skin blisters"<br>“skin complication: contact dermatitis”<br>"Skin lesions due to mechanical compression by the orthosis”<br>“Skin breakdown”
Allergy	"Allergy”<br>”Allergic reaction to device”
Pressure sores	"Pressure sores "<br>"Pressure ulcers. Uncomfortableness"<br>“Pressing and strangling of soft tissue structures by orthesis and sling."<br>"Pressure sore”<br>“Skin sore ulcers”<br>"Sores"<br>"Pressure sores"<br>“Pressure ulcers”<br>“Pressure sore”

	"Pressure events"<br>"Cast sores"<br>"skin complication: pressure sore"
Pain	"Pain"<br>"Neck pain"<br>"Neck pain"<br>"Devices site pain (distinguished from fracture pain)"<br>"neck complaints"<br>"pain and level of daily activities"<br>"Slings May affect cervical symptoms"<br>"neck pain from sling"
Compartment Syndrome	"Deep Vein Thrombosis, Compartment Syndrome,"
Deep Vein Thrombosis	"Deep Vein Thrombosis, Compartment Syndrome,"<br>"Vascular events"<br>"Vascular events like low venous comeback"
Edema upper extremity	"edema upper extremity"<br>"upper limb oedema"<br>"Hand is swollen because the sling is low"<br>"edema"<br>"edema compressions by orthesis"
Device type	"Sling, standard or abduction"<br>"Shoulder immobilizer."<br>"Sling" (3)<br>"Shoulder abduction brace or simple immobiliser or cuff collar sling"<br>"1. cast or slab 2. arm sling 3. duration of immobilisation 4. whether pendulum exercise allowed within the immobiliser."<br>"devices limiting ROM or function only"<br>"Uses of Orthosis or long arm cast along with sling"<br>"sling and cast and brace "<br>"any orthosis or sling"<br>"plaster cast, orthosis"<br>"Method of Device and duration and time or removal and exercise."<br>"sling, desault bandage, in rare cases cast"<br>"Sling Cast"<br>"arm sling"<br>"Shoulder support sling and /or arm sling"<br>"just the type of sling used and if this was use in Add, Abd or IR"<br>"anything which is used for non-operative treatment."<br>"Sling"<br>"Type an time of immobilization, start of pendulum etc"<br>"also include external fixator"<br>"What device is used? Time when device used. Can we undress device when we do X-ray, sleep, take bath"<br>"Sling and Collar and cuff"<br>"There are many devices Some are much different than others"<br>"Shoulder immobiliser"<br>"Cast & Brace"<br>"simple sling versus fixed sling"<br>"kind of sling abduction pillow? internal rotation of the forearm in Gilchrist? "<br>"Cuff Collar Sling Ultra sling Aeroplane splint Pouch arm sling"<br>"1. shoulder immobiliser 2. aeroplane splint 3. shoulder

	starpping"<br>"Sling immobilization"<br>"Shoulder sling with or without backstrap"<br>"sling collar and cuff arm-to-chest"<br>"sling gilchrist"<br>"type of the cast or sling"<br>"Shoulder arm immobilizer"<br>"Shoulder immobilizer and X-ray at 15 days post treatment "
Other comments / issues	"duration, type"<br>"Length of time of immobilization Was PT used to help recover motion and function Passive ROM started when Active assist ROM started when Active ROM started when"<br>"casts, slings, splints, bandages"<br>"how long the patient used the sling"<br>"Time to mobilization, time to full active movement allowed"<br>"Time instructed to wear a sling."<br>"Duration for U slab immobilisation ."<br>"allowed ROM active/passive time of immobilization"<br>"Self-directed discontinuation of use."<br>"removal by patient"<br>"discontinuing usage change from external device to operative treatment "<br>"If it is considered important (I am not sure how often it would be) compliance"<br>"Compliance of use, duration, position (abduction, degrees), rehabilitation protocol, active vs passive motion, time until abduction> 90°, load bearing, return to work, return to sport. "<br>“Compliance"<br>“impossibility to use, non-compliance”<br>“Non compliance by patient”<br>“Compliance"<br>"Non-compliance for the device "<br>"patients compliance to wear the device"<br>“Non-Compliance with instructions”<br>"Compliance of wearing; comfort"<br>"Patient compliance to the treatment"<br>“Difficult to assess the compliance of slings”<br>"Discontinuity in using the device"<br>"Patient compliance"<br>"unused immobilization device"<br>"Orthosis usage time. Time to do without orthosis"<br>“Problems to adjust the orthosis, thus resulting in non-usage of it"<br>"Compliance, appropriate use, adequateness of the device chosen / bought by the patient"<br>"Patient compliance Treatment failure - device not used Change of device"<br>"Difficulty with use"<br>"Undisplaced fracture"<br>“Increasing displacement of the Fx”<br>"secondary fracture dislocation”<br>"Change in reduction”<br>"Radiological change in position of fracture fragments in non operative management"<br>“alteration of fracture position”<br>“Lost of reduction”<br>"Delayed/non union"<br>"initial fracture displacement at time of injury, secondary

	displacement of the fracture, delayed union, non-union, mal-union, posttraumatic humeral head necrosis, posttraumatic omarthrosis"<br>"And event that determinerà non Union or deteriorino of Clinical situation"<br>"But probably irrelevant"<br>"Immobilization by Mayo clinic of 3 weeks"<br>“Complications local related to device"<br>"any"<br>"If a fracture is well reduced and the implant is stable, do not use external restraints If I decide non operative management 4 weeks would be the time and then it is sent to therapy"<br>"Device events may have long-term effects, for example on ROM or local integument, so observation period should be longer"<br>"any of them"<br>“Prolonged immobility”

Postoperative local events: Osteochondral events

Definition : Events affecting the osteochondral tissue of the proximal humerus, clavicula and/or scapula

Specifications : For which treatment option(s) the following specific event(s) should be documented? (several options may apply)

Events	Number of responses	non-OP n	IM n	Plate n	Other n	None n
Bone formation / resorption	182	87%	86%	90%	76%	3%
New fracture (around the implant)	189	12%	90%	94%	81%	2%
Screw / bolt cutout	188	4%	84%	98%	64%	1%
Tuberosity migration / resorption	190	81%	89%	94%	84%	1%
Head necrosis	190	84%	88%	95%	82%	2%
Delayed union / nonunion	190	91%	96%	97%	85%	1%
Fracture malunion	187	92%	93%	93%	83%	1%
Loss of fracture reduction	190	80%	91%	94%	85%	1%
Other event(s)*	16					

* This means that 16 participants reported at least one other event to be documented for any of the 4 treatment options. The list of these events is presented below.

Non-OP = non-operative; IM = Intramedullary; Plate = Plate osteosynthesis; Other = Other surgical treatment

Other event(s)	non-OP n	IM n	Plate n	Other n
"Arthritis"	x	x	x	x
"Heterotopic ossification"	x	x	x	x
"Wound infection Neurological problems Cuff problems"	x	x	x	x
"Glenoid erosion; post-traumatic OA"	x	x	x	x
"Glenohumeral arthrosis"	x	x	x	x
"Implant fracture, implant shape change(bending...)"	-	x	x	x
"Complications (with effects also for the bone) such as CRPS, pathologic fracture"	x	x	x	x
"Joint arthrosis RSD Retrauma"	x	x	x	x
"Death neurovascular disorders harware removal"	x	x	x	x
"Secondary screw penetration? (ORIF) Posttraumatic arthritis (all) Posttraumatic rotator cuff tear arthropathy (all)"	x	x	x	x
"Arthritis Tears of cuff Biceps rupture Deltoid palsy"	x	x	x	x
"1. glenohumeral joint degeneration(osteoarthritis, assault by screws, etc)"	x	x	x	x
"Self-reducing fractures in conservative treatment"	x	-	-	-
"Backout of locking head screw Migration of pins/Kwires"	x	x	x	x
"Rotator cuff dysfunction"	-	x	x	x
"Cement penetration or extravasation in to the joint"	-	x	x	-

Proposed definitions

Bone formation / resorption

"Painful movements, inability to abduct, myositis in case of head injury, instability due to inadequate fixation and mobilization "
"The removal of bone by osteoclasts. Bone formation, ossification, process by which new bone is produced"
"Any loss of bone , or new bone formation"
"Bone resorption: signal of radiological radiolucency near the fracture site   Bone formation: Radiological sign of calcium deposition and loosening the area of initial fracture"
"hypo or hyperdense new bone tissue after fr. fixation or healing"
"Radiographic appearance of calcified image or radiographic disappearance of density in bone area compared with previous radiographs"
"Change in bone quantity from prior radiographs"
"Bone resorption as AVN. Bone formation as exuberant callus or heterotrophic ossification although very rare around shoulder joint."
"xray finding"
"Change in size of fragment"
"Lucency around screw"
"comparison between immediate post-op x-ray and follow up exams"
"Progressive fragmentation of the head due to osteonecrosis Bone resorption around the screws may indicate loosening and impending failure Resorption of tuberosity bone due to osteolysis"
"As compared to baseline standard radiological views and serial progressive x-rays at definite time intervals, see for amount of new bone formed or the residual bone absorbed should be specified"
"callus score"
"Bone resorption will be appearance of radio lucency area while bone formation will be callus formation "
"Resorption is a well-defined lucency around or at the fracture site due to activation of osteoclast   bone formation is the ossification or osteogensis process at or around the fracture site due to new bone formation due to activation of osteoblast"

"Bone resorption: radiolucent lines within 3 months p.o. bone formation: evidence of callus"
"Next to the fracture you can see some additional sclerosis or lytic changes, which are progressive between two x-rays in the follow-up"
"Lysis bone formation around the implant of >1mm"
"Bone resorption is resorption of bone tissue, that is, the process by which osteoclasts break down the tissue in bones[1] and release the minerals, resulting in a transfer of calcium from bone tissue to the blood"
"Resorption of formation the new bone tissue on the serial X-rays taken in 6-8 week intervals "
"AVN of head or tuberosity"
"Formation of callus in the healing site"
"Bone resorption = lysis of bone originally present on initial xray at fracture site. Bone formation = new bone observed at fracture site"
"Callus formation around previous fracture line"
"Calcification on Xray and degree of radiolucency "
"Change of bone density or position in a proximal humeral fracture"
"Change over multiple XR. No reference"
"Regressione radiographically evident of callus, bone apposizioni periostally or interfragmentary "
"x-ray bone mass loss in the fracture zone 4-8 weeks after injury/surgery x-ray bone mass in the fracture zone 6-8 weeks after injury/surgery"
"As a result of instability "
"Resorption of the humeral head osteopenia of the humeral head resorption of the tuberosities"
"Heterotopic ossification, signs of humeral head necrosis (resorption vs. hypercalcfication); classification of AVN according to Cruess et al (1978) Experience with steroid-induced avascular necrosis of the shoulder and etiologic considerations regarding osteonecrosis of the hip. ClinOrthopRelatRes130:86-93 Lambert+, EFORT Open Rev 2018;3 DOI: 10.1302/2058-5241.3.180005."
"Radiolucency or new bone (callus seen around the fracture or implant, that was not seen in previous images "
"Change in bone shape and radiolucency seen on x-ray or CT scan."
"resorption: lucency around implants or fracture site   formation: callus evident--non- and bridging"
"Visible changes in relative radio-density on serial X-rays, narrowing or widening of the fracture gap."
"Sequential XRay location "
"Excessive amount of bone resorption. it is difficult to define excessive"
"Bone resorption -lost of any bone on x ray in fracture site or around the implant bone formation- excepting callus formation any abnormal bone around the fracture or joint (heterotopic ossification)"
"Any bone formation except from the normal callus and any resorption seen around the implant or fracture."
"Appearance of radio-lucencies in different zones (1 to 7) within six weeks to three months after surgery. Although this may be extended to six months or a year after surgery   J Shoulder Elbow Surg. 2017 Nov;26(11):1984-1989. doi: 10.1016/j.jse.2017.04.012. Epub 2017 Jul 5. Humeral bone resorption after anatomic shoulder arthroplasty using an uncemented stem. Inoue K, Suenaga N, Oizumi N, Yamaguchi H, Miyoshi N, Taniguchi N, Munemoto M, Egawa T, Tanaka Y. "
"Decreased bone density and / or exostosis"
"Bone resorption; disappearance of bone at the fracture site or around the implant   bone formation: appearance of bone between the fragments or around the implant"
"Loss or increase of bone density in image X-Ray or CT "
"Specially for the tuberosities if they resorb Avascular necrosis Head collapse   Calcifications on tendons"
"Jede Mr-tomographisch oder radiologisch erkennbare Signalstörung bzw. Konturveränderung"
"Callus description on defined X-ray projections   loss of bone structure (abnormal vs. timely due to union)"
"Widening of the fracture line; loss of cancellous bone stock; chondrolysis "
"Any change to post-operative X-ray no matter the size or location. "
"1) Postop resorption: Radiolucent lines surrounding screws or device or if non-op. radiolucent areas of the bone demonstrated in a plain radiograph. 2) Bone formation: Bone deposit or ostophytes at a non-anatomical location demonstrated in a plain radiograph "
"Changes to previous Xrays"
"Jump to search   Bone Reabsorption   Osteoclast.jpg   Light micrograph of an osteoclast displaying typical distinguishing characteristics: a large cell with multiple nuclei and a 'foamy' cytosol.   Specialty Rheumatology Edit this on Wikidata   Bone resorption is resorption of bone tissue, that is, the process by

AO TRAUMA

which osteoclasts break down the tissue in bones and release the minerals, resulting in a transfer of calcium from bone tissue to the blood. doi:10.1126/science.289.5484.1504"
"Loss of bone density around the fracture or implant"
"Loss/gain of bone compared with immediate post-op images"
"Stress shielding, remodeling of bone in unloaded zones, Spur formation"
"Resorption of bone definition of healing callus formation of at least three cortices"
"Comparison of repeated orthogonal X-ray images"
"Bone formation as any bone like image occurring out of the fracture site. bone resorption as lack of bone or increased bone gap at the fracture site. Diminishing the amount of bone on the epiphysis or tuberosities"
"Probably not"
"Loose of visible bone on x ray / new bone detectable on x ray"
"Subsidence   callous formation"
"When part of the humerus that was present disappears in follow up, same when abnormal bone formation occurs (similar to HO)"
"Bone resorption would include osteolysis around implants or in the setting of a nonunion"
"Osteopenia or obvious bone formation"
"Changes in fx line in xray controls"
"bone resorption   sorry, unknown   bone formation   heterotopic ossification in the soft tissues and the joint."
"..."
"X-p"
"Looseness of bone around the metal   periosteal bone reaction and formation "
"Presence of new bone on at least three side of bone on two views"
"Radiolucency/radio-opacity"
"Hypertrophic callus or subchondral bone resorption"
"The amount of bone loss at the fracture site / The amount of callus formed around the fracture site"
"New bone formation : callus"
"This is a little confusing, but maybe 'abundant callus formation' or 'heterotopic bone formation'"
"Visible new bone in the area of fracture"
"Callus Formation in the fracture area Gap Formation at the fracture area"
"CCD AHD gothic arc "
"Radiolucency around screws or plates and fracture site. Presence of callus on sequential follow up Xrays in AP and Axillary or scapular views. "
"Change in amount of bone around implant"
"Bone formation - bridging callus around the metaphysic that was not present before fracture healing. Bone resorption - appearance of lucency on radiograph at the fracture site"
"Bone resorption is loss of bone around screws/bolts as a sign of instability of the fracture Fixation bone formation is paraarticular ossification (prosthetic replacement) "
"Apparition of the callus or sign of AVN"
"Bone resorption - loss of bone tissue within the confines of bone perimeter. Bone formation - Appearance of new bone tissue on the outside of host bone perimeter."
"Callous formation in AP/Lat views"
"Absence or appearance of bone material, visible on x-rays "
"Radiographic evidence of change on 2 successive radiographs"
"Resorption: increasing radiolucency on follow-up X-rays Bone formation: new appearance of calcified shadows with trabecular structure on follow-up X-rays which are not secondary displaced fragments "
"Formation of bone in areas not associated with the fracture.   bone resorption "
"Change in radiological appearance   Heterotrophic ossification "
"Bridging callus"
"Implant loosening, callous bridging"
"Loss in x ray"
"Formation of extra bone of resorption of bone ends / tuberosities"
"Lucency with or without sclerotic margins / callus or "
"reduced bone density visible on x-ray"
"Callous formation Screw Halos"
"On x ray resorption of humeral head"
"Lucency of >2mm at the fracture site or bone implant interface. "
"Visibility of fx line on X-ray healing after 12 mt (yes/no)"

"Resorption of the greater tuberosity"
"Any resorption at the fracture site or of the tuberosity"
"Resorption- radiolucency in and around implant - screw/ plate   Formation: callus formation/ increased bone density, loss of visualization of fracture lines, continuity of bone on either side of fracture site"

New fracture (around the implant)

"A break in bone or cartilage, confirmed on x ray. New radiolucent line around implant"
"Any fracture complete or incomplete around any type of implant"
"Signal of osteolysis around the implant"
"New fr. line after ORIF or CRIF on x ray"
"Fractures of the humerus below the implant or through the implant"
"New fracture on XR"
"Stress shielding at the plate end."
"xray finding"
"New radiolucency line that connects to the implant"
"Has to be classified as displaced or undisplaced"
"Comparison between immediate post-op x-ray and follow up exams"
"Fracture within 5 cm distal to the tip of the implant. Beyond this, the existing implant may not have a big bearing on how the peri-implant fracture is treated. "
"As compared to baseline standard radiological views and serial progressive x rays at definite time intervals see for any fracture around the implant "
"Any new fractures that were not evident in the pre-operative radiograph."
"New fracture occur at the tip or around the implant intra-operative or post-operative"
"Self-explaining. specification with classifications for perimplant and periprosthetic fractures of the proximal humerus (e.g. Vancouver classification)"
"New fractures / fracture lines around the implant, which were not present before the operation"
"Any loss of cortical continuity around the implant or within 2 bone widths/diameter of the tip of the implant"
"New fracture line around the border of the implant"
"Peri-implant fracture from a new injury or iatrogenic"
"Anything visible on an xray. "
"Fractures at screw or plate interface, or nail entry or tip"
"New fracture line in same radiological view that was previously not present "
"Fracture line on x ray"
"Any new disruption of cortical integrity"
"New fracture line not observed on "
"Break in cortical bone. NO reference"
"And New Line of radio-transparency"
"X-ray confirmed fracture line in the area of implant location with clinical manifestations (pain, loss or reduction of segment function)."
"Imposibilita to get. Bone contact , and. No restitution of the calcar "
"Would seem somewhat self-evident, but fracture line about implant"
"Fracture at the junction plate and diaphysis"
"Definition should require new trauma, peri-implant fracture line, new fracture line in comparison to initial fracture"
"New fracture lines not seen in the pre-op films"
"Fracture line seen around the implant on x-ray or CT scan on the humerus, glenoid and acromion."
"New fracture line around implant"
"New fracture communicating with the implant."
"Appearance of new fracture lines around the implant on X-ray"
"Location topography of the fracture   relation with screws/implant "
"Any implanted side humeral fracture "
"Duncan CP, Haddad FS (2014) The Unified Classification System (UCS): improving our understanding of periprosthetic fractures. Bone Jt J 96-B(6):713-716 Chan, L.W.M., Gardner, A.W., Wong, M.K. et al. Non-prosthetic peri-implant fractures: classification, management and outcomes. Arch Orthop Trauma Surg (2018) 138: 791. "
"Any fracture that occurs after the main fracture near the implant."

"Fractures occurring around the implant graded according to the Kirchoff classification   Kirchhoff C, Kirchhoff S, Biberthaler P. Classification of periprosthetic shoulder fractures. Unfallchirurg 2016;119:264-72.   Kirchhoff C, Beirer M, Brunner U, et al. Validation of a new classification for periprosthetic shoulder fractures. Int Orthop 2018;42:1371-7."
"Occurrence of a new fracture at the limit of osteosynthesis"
"Fracture line in direct relation to the implant fracture in the same segment whose treatment should take into account the implant in situ"
"New fracture lines after implant fixation"
"Hidden fractures non diagnosed preoperatively and they displace while fixing the fracture Periprosthetic fractures: while reaming or implanting the definitive stem. Multifragmentary fracture of greater tuberosity which is missing to fix and displaces postoperatively. Fractures while drilling, specially wide screws in nails. Fractures while reaming for a nail Fractures while inserting a nail"
"Opening of fissure lines, periprosthetic fractures, separating the shaft during nailing"
"A post-operative new fracture around the implant at any time."
"Jede Fraktur im Bereich des Implantates und unterhalb davon (Vancouver)"
"A new fracture of the proximal humerus next to the plate or screws without new trauma."
"Any fracture line near an hardware."
"Any change to post-operative X-ray no matter the size or location."
"Radiolucent lines with or without displacement of the bone in relation to the device, or starting from any the level of the device."
"Development of new radiolucent line"
"Classification according to Worland https://doi.org/10.1016/S1058-2746(99)90095-2"
"Loss of continuity in most of cases due to a traumatic injury that causes that lesion near the implant"
"Previously un-observed fracture"
"Always possible"
"Radiographic visible fracture, conventional or CT"
"New fracture mostly distal from implant"
"Repeated orthogonal X-rays "
"Any fracture line , not present at the at the initial presentation, that is seen on subsequent X-rays at proximal or distal the implant site. Any fracture detected during operative fracture which was not the original one and attributed to handling for reduction or implant positioning"
"Only if articular injury involved"
"Breakage of bone continuity"
"Radiographs"
"Any fracture (broken bone) around an implant"
"Fracture occurring at a site distinct from the original injury"
"Obvious"
"New fracture line in x-ray controls"
"The fracture lines extending from the implant and its tips."
"x"
"Fracture of humerus next to implant"
"X-p"
"Fracture within the range of 1-5 cm proximal and distal to the fixation device "
"New fracture at the side of screw holes or at the end of implant without efficient trauma"
"Appearance of a fracture line on X-ray "
"Name says it all"
"It is the discontinuity of bone at or below the level of the implant"
"We get cases fractures just below the plate due to fall on that operated limb"
"Stress fracture"
"'Peri-implant fracture'"
"Fracture line in different area than original fracture, not more than 2 cm from the implant"
"Postoperative new fracture line on conventional X-ray"
"Descriptive"
"New fracture line in the bed of/ proximal or distal to the implant on Xrays which was not present earlier ."
"Any periprosthetic fracture distal to plate"
"New fracture lines that did not exist prior to surgery"
"Mostly fracture distal or adjacent to the end of an intramedullary device"
"Implant near fractures-a fracture below the end of a plate"
"Apparition of a new line of fracture "

"Appearance of new discontinuity in bone around the area of fracture/surgery with use of implantable device."
"Radiographically apparent fracture (CT or plain films)"
"Fracture involving the bone around the implant, to be a postop event, a new trauma after the surgery is necessary"
"Evidence of fracture within the same bone not present at time of insertion"
"Any new fracture line"
"New fracture about implant that was not there immediately post operatively"
"Those that cannot be fixed with a separate implant "
"Stress riser fractures"
"Any detected (in doubt CT)"
"x ray"
"Periimplant fractures through or just below the implant"
"Sharp, acute discontinuity of bone, in a position not fixed by the implant"
"Any new fracture around the implant"
"Another fracture line or an intraoperative fracture with implant implantation"
"Tuberosity fracture or periprosthetic fracture as a stress fracture at the end of the plate especially in a rigid locking construct"
"Any loss of continuity of the implanted devices or its parts"
"Any new fracture"
"Any new fractures occurring after surgery around the implants"
"Occurrence of fracture line which was not seen in preoperative X-rays / which was not there intra operatively"

Screw / bolt cutout

"Loss of reduction - varus reduction, comminution at proximal end"
"Increased prominence of screw or bolt on x ray"
"Screw or bolt out 4 mm of the plate or nail or bone , as minimum"
"x ray visible prominence of screw across hared circumference"
"Radiographic image of penetration of the screw through the humeral head inside the joint"
"Hardware extrusion compared with immediate postop imaging "
"Bone resorption in screw tip in humoral head or implant failure causing bolt cut out."
"Xray finding. John Gorzaga JOT paper"
"Protrusion of screw/bolt tip through distal bone cortex/surface"
"Based on X-ray appearance"
"Comparison between immediate post-op x-ray and follow up exams"
"Secondary penetration into the joint or outside the bone on orthogonal views. There should be no primary screw/ bolt violation of the joint/ humeral head on initial post-surgery X-rays "
"As compared to baseline standard radiological views and serial progressive x rays at definite time intervals, see for loss of initial position of screw or bolt. see for cut out of screw or bolt"
"Any migration of more than 5mm"
"Migration of the screw within or outside the bone most common complication after plating fixation "
"Secondary screw perforations should be excluded by CT scan. Alternatively a defined series of imaging may exclude primary screw perforation"
"Screws / bolts protruding the cortex on the tip side"
"Movement of an implant / screw / bolt >1 width/diameter of the screw/bolt or >3mm of the implant from its original position on exactly comparable images"
"Change of the position of the screw / bolt on the control X-rays compare to the immediate intraoperative X-rays control"
"Screws placed too deep and through the articular surface, or late penetration from fragment settling or from AVN. "
"Anything visible on an xray that alters the treatment or outcome. likely to be all events"
"Migration of screw or bolt from original intended position "
"Changed position of screw or bolt in identical view"
"Displacement of the implant and lucent line around it "
"Movement of implant from where it was placed"
"Perforation of screw\bolt into articular surface with progression from post OP xrays"

AO TRAUMA

"Change in position of screws with reference to articular surface. No reference"
"Protrusion from cortical- articular surface "
"X-ray confirmed migration of the screw with bone dilution in the area of its initial installation"
"Breakage or protrusion into the joint "
"Migration that is visible on plain films. from relative position on lateral cortex"
"Displacement between hardware and bone "
"Screw cut out is a penetration of the screw tip through the subcortical bone. However, reliable detection requires CT or specialized x-ray projection. Injury. 2014 Oct;45(10):1557-63. doi: 10.1016/j.injury.2014.05.025. How many radiographs are needed to detect angular stable head screw cut outs of the proximal humerus - a cadaver study. Spross et al"
"Screw changing position post op, and outside the bone border"
"Screw/bolt position out of the bone as seen on any of the x-ray projection or CT scan image due to implant migration or bone resorption"
"Catastrophic change in position of the device leading to failure of head fixation   Would also add backout"
"Tip of the screw/bolt not sitting entirely within the bone"
"Penetration of the screw/bolt beyond the anatomical confines of intended bony region that it was placed in. Eg, articular penetration of PHILOS screws"
"Bone displaces relative to the angle-stable screws which remain fixed to the plate   +/- penetration of the joint "
"Prominence of implant "
"Any intra-articular screw penetration."
"Any migration of screw with penetrating articular surface"
"Any penetration in the articular surface,"
"Any change in the position of screw / bolt in three successive xrays AP and Y views projected identically six weeks apart"
"Mobilization of an osteosynthetic screw from its initial location during the assembly"
"Migration of the screw within the bone cutting the articular surface"
"Displacement of bone/implant causing presence of screws out of the bone"
"The head rotates while the screws remain locked, thus cutting the head joint surface. It is basically a loss of reduction the construction cannot support."
"Penetration through the head"
"Penetrating the cortex of the articular side of the humeral head at any time"
"Radiologisch darstellbarer Überstand einer Schraubenspitze in Relation zur Humerusgelenkfläche (corticaler Rand) welcher nicht schon intraoperativ bestand"
"Screw backing out change in position of screw penetration of the articular surface of bone with the screw tip"
"Perforation of the humeral head (cortex) by one or more screws with complaints of crepitation with subsequent revision or perforation without complaint but with revision because of a surgeons decision."
"Movement in mm on comparable X-ray projections Cave: differentiate vs. cut-through ! (possible) joint perforation yes/ no"
"Change of position respect the healthy bone in secondary Xray"
"Any change to post-operative X-ray no matter the size or location. "
"In relation to the glenohumeral joint: 1) Primary cutout caused by screws / bolt to long penetrating the surface of the cartilage of the head 2) Secondary cutout either by 'sinking' of the head fragment (in the direction of the screws) OR the event that displacement of the head take place and there is a real 'cutout' (ie. penetrating the surface of the head cartilage) due to head displacement."
"Changes of place of screw as compared to old xray"
"Perforation of screw tip through head Fragment doi: 10.1007/s00264-017-3652-6"
"Intra-articular penetration of the screws"
"Loss of position with the original fragment"
"Distinguish articular of sub-acromial from benign."
"esp in locking plates"
"Joint Perforation in one xray"
"Perforation of the head"
"As above"
"Epiphysial perforation at its periphery"
"yes"
"Migration of implant through into the joint"

AO TRAUMA

"Radiographs"
"When the screw / bolt protrudes into the humeral head"
"Change in position of a screw/bolt from intraoperative/first postoperative xray to most recent xray"
"Obvious"
"Screw or bolt displacement in xray controls"
"The penetration of the tips of the screws out of the joint surface."
"x"
"Doh"
"X-p"
"A screw pulling or cutting with it a circumferential bone "
"Change in position of the screw relative to the bone with loss of reduction"
"Mostly screw penetration during head displacement in locking plate fixation"
"The migration of the screw or bolt through the bone"
"In old age, osteoporotic bones, in diabetic patients we get screw or bolt cut out cases"
"Osteoporotic Fractures"
"Displacement of the bone and/or screw/bolt in which inherent stability provided by initial implant placement is lost."
"Visible perforation of the screw/bolt through the head cartilage"
"Difference in position between intra/postop x-ray and further follow up x-ray"
"Descriptive"
"Screw penetration on sequential X-rays"
"Joint penetration by any of screw or bolt"
"Backward migration of locking bolts of an imn"
"Clinical and radiological diagnosed penetration of screw/bolt/wire through the humeral head in the joint space"
"Loosening of screws"
"Violation of humerus head perimeter by implant device in any one radiological view."
"Penetration of screw into glenohumeral joint of outside of humeral head"
"Screw mobilization"
"Radiolucency/breakage or change in position in consecutive xrays"
"Penetration of the tip of screw / bolt through subchondral bone layer"
"Migration of screw or bolt from immediate post op XRs"
"Aftermath of implant loosening"
"Any visible"
"x ray"
"Cutout of screw into humeral head radiographic images"
"Protrusion beyond the chondral surface in any x-ray plane"
"Any screw / bolt penetrating the articular surface"
"Xray evidence of same"
"Screw penetration into the joint due to varus collapse"
"Penetration of the hardware through intact bone adjacent to the implant with loss of alignment"
"Head screw cut out in any radiographic projection"
"Any change in screw position"
"Any screw cut out that occurs after surgery"
"Migration of screw from its initial post op position - mostly breaching cortex or articular surface"

Tuberosity migration / resorption

"Unreduced tuberosity, poor fixation of tuberosity"
"Radiological signs, increased radiolucency or migration of tuberosity"
"The humeral head height (HHH) was measured on the anteroposterior radiographic image by calculating the vertical distance between the tangent line of the highest point of the humeral head and the greater tuberosity L. Bai, et al Radiological evaluation of reduction loss in unstable proximal humeral fractures treated with locking plates, Orthopaedics & Traumatology: Surgery & Research, Volume 100, Issue 3, May 2014, Pages 217"
"Different contour or position of tuberosities in follow up period"

AO TRAUMA

"Radiographic image of disappearance of the greater tuberosity or change of position of the greater tuberosity with respect to previous radiographs"
"Change in position compared with immediate postop imaging"
"Malreduction of tuberosities or bony resorption will lead to failed construct and rotator cuff insufficiency."
"X-ray finding but may need CT scan"
"Displacement of tuberosity of more than 2 mm - Reduction in size of tuberosity including previous measurement as separation from other fragments"
"Displacement more than 5 mm and compared to immediate post op. for conservative it is change in position from initial X-rays"
"Comparison between immediate post-op x-ray and follow up exams"
"Any change in tuberosity / tuberosity fragments position compared to initial post surgery X-rays. Fragmentation/ osteolysis of tuberosity fragments define tuberosity resorption"
"Migration more than 5 mm in any direction"
"Baseline comparison with standard radiological views and serial progressive x rays at definite time interval, any change in size and position of the bony structures like tuberosities "
"1. Migration - any displacement of greater tuberosity altering the head to tuberosity distance as evident by change in dimensions from the immediate post-operative radiograph. 2. lucency of greater tuberosity, thereby reducing the profile of it to be considered for resorption."
"Displacement more than 5 mm "
"Analysis of tuberosity Position and quality in comparison to contralateral side "
"Between the initial postoperative and the follow-up x-ray, there is clear migration of the tuberosity, at least 0.5cm Resorption: if the fragment get's smaller"
"Movement / resorption >2mm from original position on exactly comparable images"
"Change of the position of the tuberosities or the change of the size of tuberosity on the control X-rays compare to the immediate intraoperative X-ray control"
"Loss of tuberosity reduction changes from initial postop imaging"
"Anything altering the relationship of the cuff to the head. Resorption that does not result in loss of cuff function is physiological rather than pathological"
"Rise or detachment of the greater tuberosity"
"Tuberosity migration = redisplacement or tuberosity from original intended position after internal fixation or initial xray for nonoperative treatment. Tuberosity resorptive = lysis of bone in tuberosity compared to initial xray"
"Distracted tuberosity position in identical view that was not present previously "
"Relation between the head of the humerus and the acromion "
"Movement from where it was or loss of bone density"
"x-ray showing any displacement in tuberosity. repeated x-ray until the time of revision or end of follow-up with progressive resorption"
"Change in position of tuberosity. NO reference"
"Comparable variation of position of tuberosity"
"Radiographically confirmed migration of tuberosity more than 3mm /decrease in bone density of tuberosity by 1.5-2 times from the bone"
"Elevation of the greater tuberosity, loss of range of motion. "
"Migration is movement of more than 2mm from post op position. "
"Displacement on consecutive X rays osteopenia of the tuberosities"
"Is defined as displacement of the tuberosity of more than 5mm in comparison to postop radiographs (again CT scan or defined x-ray). Tuberosity resorption can be defined as radiological disappearance of the tuberosity or a combination of clinical signs of rotator cuff insufficiency in combination with radiological resorption. I could not find reliable literature so far on a definition in this case."
"Further displacement of the tuberosities on CT, more than 5mm"
"Change in tuberosity position and bone quality detected on x-ray or CT scan."
"Tuberosities change position from intraoperative radiographs postoperatively resorption--tuberosities disappear; unable to see the bony anatomy"
"Where tuberosity was confirmed in a satisfactory position on intraoperative or early postoperative imaging but has subsequently migrated or resorbed."
"Progressive Posterior and/or Superior shift of tuberosity identified in serial follow up X-rays."
"Sequential X-ray mm in relationship to the humeral head "
"Dislocation of tuberosity more than 3-5mm or extinction of tuberosity on x-ray,"
"More than 5 mm displacement / complete resorption of the tuberosity"

"Any change in position of the tuberosity more than 5 mm from the post operative position after either ORIF or Arthroplasty   Factors associated with failure of tuberosity osteosynthesis need to be mentioned 1. Poor initial position of the prosthesis (specifically, excessive height and/or retroversion) 2. Poor position of the greater tuberosity 3. Women over age 75 years (likely with osteopenic bone).   Tuberosity malposition and migration: Reasons for poor outcomes after hemiarthroplasty for displaced fractures of the proximal humerus P. Boileau, S.G. Krishnan, L. Tinsi, G. Walch, J.S. Coste, D. Molé   J Shoulder Elbow Surg 2002;11:401-12   DOI: https://doi.org/10.1067/mse.2002.124527"
"Displacement and / or resorption of tuberosities of the proximal end of the humerus"
"Displacement or dissapearance of tuberosities"
"Resorption: There is a greater tuberosity that decreases its density and size in consecutive postoperative X-rays. Migration: the tuberosity or one fragment of the tuberosity with the supraspinatus insertion attached is not fixed, either with sutures or screws, to the implant. Or the fixation fails postoperatively."
"Loosening of Fixation or Resorption "
"Tuberosity displacement of >10 mm inferior or superior to the tangent line of the head   Bahrs C, Badke A, Rolauffs B, Weise K, Zipplies S, Dietz K, et al. Long-term results after non-plate head-preserving fixation of proximalhumeral fractures. Int Orthop 2010;34:883-9.http://dx.doi.org/10.1007/s00264-009-0848-4"
"Jede Stellungsänderung der Tuberkula im Vergleich zum intraoperativen Befund / Zunehmende Transparenz der Tuberkula bis zum völligen Verschwinden im konventionellen Rö"
"Displacement of more than 1 cm compared to intra-operative images"
"Secondary displacement of more than 2 mm of the greater or lesser tuberosity compared to operative Images."
"Migration: 3-D-movement description resorption: loss of normal bone structure"
"Loss of bone stock; changes of the position respect post-op Xray check"
"Any change to post-operative X-ray no matter the size or location. "
"1) Migration: Position of tubercles has changed from their initial position on a plain radiograph during FU. 2) Resorption: Radiolucency of the tubercles during FU at 3 months or later"
"Changes in subsequent Xray"
"Redislocation or absence of tuberosities after ORIF or prosthetic replacement in proximal humerus fractures"
"Loss of bone density and loss of reduction and secondary malalignment of tuberostiy"
"Change in tuberosity position from immediate post-op films   loss of bone mass"
"After reduction and fixation in fracture hemiprosthesis or after plating (not so rare)"
"Regular position, under head line, above head line in AP view"
"Vanishing of tuberosity or loss of reduction"
"As above "
"Displacement of tuberosity from its anatomic position. It is migration if it were appropriately reduced primarily and mal reduced if it is displaced just after the osteosynthesis. resorption if it was present in the immediate post op X ray and no more at follow up"
"if affects joint surface"
"dislocation / dissolution ?"
"radiographs"
"When tuberosity displaces more than 5 mm or disappear in the follow up"
"Migration: Displacement of the tuberosity from original reduction/anatomic position resorption: Gradual bone loss of tuberosity after initial stable healing"
"Movement or osteopenia"
"Displacement / 'vanishing' tuberosity in xray controls"
"greater tubercle disappearance during the follow-up"
">5mm"
"X-p"
"g .tuberosity partial or total detachment and displacement with loss of bone mass"
"A proximal shift of the a tuberosity of more than 2cm"
"Displacement of tuberosities in the follow-up"
"The distance migrated by the tuberosities following a fracture"
"In comminuted tuberosity cases we get migration of tuberosity."
"Displacement of tuberosity"
"Displacement of the greater tuberosity from its injury/postop position; or resorption of the bone."
"Tuberosity migration - sequential change of position of tuberosity with respect to the head resorption - no presence of the bone in the area of greater or lesser tuberosity visible on x-ray, CT or MRI"

"Difference in Position between intra/postop x-ray and further follow up x-ray"
"Descriptive or measurement by CT"
"Proximal, anterior or posterior migration on sequential Xrays"
"Tuberosity migration compared to previous xray"
"Malposition of the tuberosity - GT - superior to the humeral head."
"Secondary displacement/resorption of the greater or lesser tuberosity on x-rays during follow-up period (comparison between time at surgery and 6 weeks/3 months)"
"Secondary displacement of the tuberosity or apparition of AVN "
"Change in position of tuberosity with immediate post-operative image as benchmark."
"Displacement of greater than 2mm"
"Mobilization of a tuberosity that usually follows the tension of the muscles resorption of a tuberosity probably following necrosis"
"Change in position/loss of greater tuberosity bone of the greater tuberosity over two radiographs"
"Migration: secondary displacement of tuberosity fragment   Resorption: increasing radiolucency or complete disappearance "
"Movement of more than 5mm of tuberosity from post operative imaging for migration. any direction. resorption would be disappearance of tuberosity over time on radiographs"
"Whether primary or secondary displacement of fragments"
"> 3mm"
"x ray"
"Loss of reduction of the tuberosity from its position or its resorption"
"Proximal migration into subacromial space; radiographical disappearance of the tuberosity"
"Migration > 2mm on xray"
"see above"
"Greater tuberosity resorption or avulsion"
"Displacement of the tuberosity of > 5mm when adding displacement seen on True AP and axillary views.
"
"Boileau JSES 11 (5), 401-12, 2002"
"Any change in xrays appearance"
"Migration or resorption that occurs post op"
"Change in position of tuberosity from immediate post op X-ray/ reduction in size/ lysis of tuberosity"

Head necrosis

"Loss of specificity, cystic appearance, increase in density, MRI - CT observations"
"Bone death resulting from poor blood supply to an area of bone"
"Cruess Classification (stages) Stage I Normal x-ray. Changes on MRI. Stage II Sclerosis (wedged, mottled), osteopenia. Stage III Crescent sign indicating a subchondral fracture. . Stage IV Flattening and collapse. Stage V Degenerative changes extend to glenoid (2006) Classifications of necrosis of the humeral head. In: Classifications and Scores of the Shoulder. Springer, Berlin, Heidelberg"""
"Deformity of humeral had or change of density"
"Destructive image of the shape of the humeral head or condensation of the humeral head. In these cases I would request MRI"
"Sclerosis or collapse on XR"
"Secondary to AVN mainly four part proximal numeral fractures."
"X-ray finding. MRI with metal suppression may help. "
"Sclerotic and/or deformation with reduction in size of humeral head"
"Sclerosis or fragmentation"
"Comparison between immediate post-op x-ray and follow up exams and MRI "
"Axial head collapse with secondary screw penetration Fragmentation of the humeral head fragment Signal changes in the humeral head MR for confirmation"
"As compared to baseline standard radiological views and serial progressive x rays at definite time intervals, compare size of head"
"Sclerosis with collapse and altered globularity of the head occurring up to about 18 months from surgery."
"A pathological process that results from interruption of blood supply to the humeral head without relation to the type of fixation"
"Structural changes of humeral head within 6-24 months"

AO TRAUMA

"New sclerosis, or lytic changes, or irregularities of the head"
"Loss of area of >10% as determined from comparable AP images measured using maximum length & width of the head across anatomical neck and centre head to lateral cortex"
"Radiological changes in the form and the appearance of the humeral head "
"Head Collapse on subsequent X-ray"
"Symptomatic or radiologically visible collapse. temporary loss of blood supply, such as on an mri scan, is not important or in the control of the treating surgeon"
"Condensation deformation and / or collapse of the humeral head by radiographic control"
"Avascular necrosis X-ray signs in head, lysis and sclerosis"
"Change in shape of head "
"Sclerosis the deformation "
"Change in the integrity of the humeral epiphysis over time"
"Sclerosis in X-ray of the head, or head resorption"
"Increase in sclerotic subchondral bone across multiple XR. No reference"
"Deformation of head or densification of head trabeculae"
"Radiographically confirmed reduction of bone density of the head by 1.5-2 times from the adjacent bone with a decrease in its diameter, deformation of the shoulder joint and the axis of the humerus "
"Collapse and screw protrusion, head resorption "
"Reduction of the width of the humeral head on AP Xray or CT Scan Lacunar spaces on CT Scan Absence of signal on MRI"
"For head necrosis I again suggest to adapt the definition by Cruess et al (1978) Experience with steroid-induced avascular necrosis of the shoulder and etiologic considerations regarding osteonecrosis of the hip. Clin Orthop Relat Res130:86-93"
"Signs of AVN on Xrays and MRi with starting head collapse. "
"Change in the shape, volume of the humeral head or the radiolucency of the bone in the region of the humeral head"
"Cystic changes and/or sclerosis of the head, with or without head collapse"
"Change in density / contour of the humeral head or development of subchondral fracture"
"Loss of smooth contour of the head, with collapse and decrease in size on serial post op follow up X-rays"
"On CT location/extent / % of the HH surface"
"Any head necrosis detected by X-ray or CT or MRI"
"Increased pain and a changes on the radiographs, which showed AVN - classified according to the system of Cruess. Cruess RL. Experience with steroid-induced avascular necrosis of the shoulder and etiologic considerations regarding osteonecrosis of the hip. Clin Orthop Relat Res. 1978;130:86-93"
"Head necrosis confirmed by complementary test (x-ray, MRI or CT)"
"Self explanatory. If found after fixation and not initially seen on plain radiographs To correlate Hertel's criteria 1. Posteromedial metaphyseal fragment size 2. Intact or disrupted medial hinge 3. Sub capital fracture or not"
"Progressive destruction of the humeral head by trauma-related devascularization"
"Loss of shape an alterations in bone density"
"The head diminishes its size and losses its sphericity in the follow up. Areas of bone necrosis are found in the head or the joint surface becomes rough. It can affect to the whole head or just a segment. "
"if the head structure loses his form - mostly seen in CT"
"Head necrosis according to Fica classification via MRI and CT-scan and or x-ray"
"Entmineralisierung und Entrundung des Humeruskopfes ggf. mit MR-tomographisch nachweisbarer Minderdurchblutung"
"Collapse of humeral head"
"Shoulder complaints (clinical signs) together with signs of necrosis on x-ray."
"Loss of contours, shrinking and deformation of head, irregularity of head articulation"
"Collapse of the humeral head with sclerosis of the subchondral bone"
"Any change to post-operative X-ray no matter the size or location. "
"This is not clearly and uniform defined by different authors, thus reports of HN is very uneven. To me it means 1) a clear and visible change in the trabecula in the head at"
"Loss of shape and change in density of head"
"Classification acc. Cruess RL (1986) Osteonecrosis of bone. Current concepts as to etiology and pathogenesis. Clin Orthop 208: 30-39”
"Deformity of the humeral head secondary a loss of vascularization due to surgery or fracture"
"Loss of contour of the head and/or collapse"

"Always possible in displaced fractures, head split. fracture dislocation and any kinds of treatment"
"Irregular surface of humeral head, 1, 2, 3 or 4 quarter of the head in AP and axial view"
"see above"
"As above"
"Hard to define early. After 6 months onward , by X ray images. If suspicious but not clear use MRI criterias"
"The crescent sign of avascular necrosis is seen on conventional radiographs and refers to a linear area of subchondral lucency seen most frequently in the anterolateral aspect of the proximal humeral head. It indicates imminent articular collapse."
"yes"
"Incongruence of head and narrowing of head "
"Radiographs - could use the criteria of Boileau et al., 2001J Shoulder and Elbow Surg"
"Collapse of the humeral head"
"Areas of articular surface collapse of the humeral head following fracture or fracture surgery"
"Loss of blood supply Sclerosis Crescent sign"
"Sclerotic or 'Vanishing' head in X-ray controls"
"The sclerosis of the cartilage and the loss of the native curvature of the humeral head during the follow-up"
"x"
"Analogue to DARCO"
"MRI"
"Partial or total AVN"
"Localised lucency on x-ray involving least one third of the head"
"Radiologically defined head necrosis"
"The degree of cell death and resorption of bone due to loss of blood supply"
"Fracture dislocation"
"Irregularity and collapse of the humeral head"
"Loss of anatomical bone architecture of the humeral head joined with cartilage destruction visible on x-ray or CT. "
"Osteolysis in the head Segment, subsidence of the Joint surface"
"Descriptive"
"X-rays at one year."
"Any collapse or sclerosis of head fragment "
"Obvious - collapse of the humeral head with loss of the round contour and possible screw penetration to the joint"
"(a) Subchondral sclerosis (b) Subchondral collapse and irregularity of the head (c) Total collapse of the head and destruction of the Joint   according to cruess classification"
"Radiological sign of AVN"
"Alteration in shape of humerus head with sclerosis."
"Changes to head shape or morphology on plain x-rays"
"Head reabsorption following interruption of nutritive vessels"
"Collapse of the humeral head as seen on radiographs"
"Partial or complete resorption "
"Tough one too because not sure if you want to factor in advanced imaging or not"
"Radiological detection"
"Should undergo CT"
"x ray+mri"
"Flattening/ loss of shape of articular humeral head or signs of AVN on MRI"
"Flattening / deformity of the humeral head, with accompanying subchondral sclerosis"
"Collapse of articular surface"
"see above"
"Avascular necrosis of the head of humerus"
"Loss of trabeculation at the subchondral bone with or without head collapse. "
"Collapse of head circle (measured with head circle on X-ray)"
"Any evidence of necrosis with or without collapse"
"Sclerosis of head in follow up period"

Delayed union / nonunion

"Failure to appear callus after 3 months"
"A nonunion is an arrest in the fracture repair process. A delayed union is defined as a failure to reach bony union by 6 months post injury"
"No show union as minimal in 3 months post surgical.   "
"Delayed visibility of bone healing after expected period of time appropriate for shoulder fracture"
"Absence of bone callus formation after 6-8 months from the fracture, and unchanged with respect to previous radiographs. In these cases I would check diagnosis with CT scan"
"Shift in fracture or hardware "
"Absence of healing beyond six weeks for delayed union and beyond six months for nonunion."
"Time : 3, 6, 9 months?"
"X-ray and CT scan"
"Persistence of fracture line after three (3) months"
"Based on serial X-rays and no signs of progressive healing"
"Comparison between immediate post-op x-ray and follow up exams"
"Persistence of fracture line at the level of surgical/ anatomical neck Implant failure Tuberosity migration"
"16 weeks"
"As compared to baseline standard radiological views and serial progressive x rays at definite time intervals, see for progress of union   standard definitions of Delayed and non-union"
"No / very slow progress of callus score. Especially the medial continuity which might require intervention."
"Delayed union is a failure to reach bony union by adequate time post-injury   non-union is permeant failure of healing mostly to the surgical neck and tuberosity fracture"
"Signs of lack of healing after 3-6 months"
"Not united between 2 and 6 or later than 6 months Question is how to define bony union: at least three cortices do have bony contact now"
"Non union - failure to show progression of healing for >\= 3 months after 4 months or more from injury Delayed union - not clinically or radiologically healed after 4 months from injury"
"Absence of bony union, persistence of fracture lines on post-operative X-rays taken at 12 weeks or later post-operative"
"After 6 months"
"Failure of healing by 6 months or loss of position of implant/bone at any stage"
"Nonunion of the fracture after 6 months"
"The lack of bone passage in the fracture site with increased gap in the fracture line"
"Usual definition"
"Delay- no callus formation in 8-12 wks   Nonunion- no callus in 6 months at fracture site"
"Delayed fracture healing more than 6 months "
"Failure to unite over a usual time for healing"
"Noted delayed progression of fracture healing by treating surgeon"
"No bridging callus at 3 months delayed. At 9 months nonunion."
"No evolvono bone formation"
"The lack of radiological and clinical signs of Union of bone after 8 to 12 weeks from the date of injury or surgery"
"Gap and lines with sclerosis between the fragments. "
"Lack of progression of healing on consecutive XR checks 6 weeks apart after the 6 month mark"
"As delayed non-union our used definition is radiological non-union after three months, while a non-union is radiological non-union after six months of treatment. Non union may radiologically be confirmed by the RUST criteria as used for tibial noon-unions (Kooistra et al; J Orthop Trauma 2010;24:S81-S86)"
"Delayed 4 months, nonunion of fracture (still visible on CT without bridging callus more than 6 months"
"Absence of the appropriate signs of the fracture healing on x-ray and CT scan in all fracture lines."
"No progression of callus after 3 months"
"Delayed - failure to show any signs of healing for at least 6 months. Non-union - lack of progress of healing on sequential x-rays beyond 6 months or no evidence of healing at 12 months and confirmed on CT scan. "
"More than 6 months after fracture, an open fracture gap visible on X-ray, resorbed fracture ends or hypertrophic callus with an open fracture line. Clinically, abnormal mobility at fracture site with or without pain."
" CT "
"Any non-union detected by X-ray or CT after 6 months from injury or operation"

"Delayed healing - if there was failed consolidation radiographically 4 months after trauma. Non-union after 6-8 months after trauma."
"Delay: A fracture without signs of union after 4 months. Non-union: A fracture without signs of callus progression within 6 months."
"Union not occurring in three months (Delayed union) Union not occurring in six months (Non union)"
"Late or no consolidation after six months"
"non-union: a fracture that do not shows signs of healing on 2 consecutive x rays after the given period of healing and related to clinical symptoms "
"Loss of progression of bone union"
"There is lack of trabecular continuity through the fracture fragments. The borders of the bone fragments appear to be ossificated. Sometimes a proper joint can be found."
"not healed after 12 weeks - delayed not healed after 6 months - non-union"
"No signs of healing 3 months postop and non-union no signs of non-healing 6 mths opostop verified by CT-scan"
"Ausbleibende knöcherne Heilung über 6 Monate nach Fraktur hinaus"
"non-union - no bone healing 6 months following fracture treatment (no progression in healing over 3 month period) delayed union - incomplete bone healing at 6 months"
"delayed Union: no cortical bone healing of at least 3 cortices in 2 directions on x-ray at 6 months. non-Union: no cortical bone healing of at least 3 cortices in 2 directions on x-ray at 9 months"
"Non-union scoring system; Weber & Cech"
"Failure to union after at least 4 months"
"According to well established definition"
"Non-op: Delayed union: >2 months Non-op: Non-union > 4 months (?) ORIF with locking plate: Consolidation expected longer: Difficult to state delayed union, but >12 months non-union"
"No changes in fracture line in subsequent Xray"
"DOI: 10.1097/TA.0b013e3181469840 doi: 10.1097/01.blo.0000195679.87258.6e , "
"Non-healing of the fracture after six months in case of non-union or non healing in the first months after the fracture. "
"Beyond six months"
"esp in subcapital fractures, mostly regarding tuberosities"
"Fracture line in AP view visible > 3 months post trauma"
"Non union after 9 months delayed is union between 3 months and 6 months"
"As above"
"If clinical signs after 4 months or fracture gap without bridges after 4 months X ray"
"Only if affects joint surface"
"Healing not achieved in expected time/ healing not achieved in time 2 times longer as is needed for fracture healing "
"Radiographs - difficult as per Morshed, Adv Med 2014 "
"Any fracture that does not heal in 9 months (non union) or does not progress in 3 months follow up"
"Lack of evidence of the fracture moving as a unit by 6 weeks postfracture (nonoperative) Lack of evidence of bony healing by 6 months after fixation/arthroplasty"
"Time period is controversial May add atrophic vs hypertrophic Infected vs non infected "
"Persistent or increased fracture gap through time"
"No callus formation at the three months after the injury."
"Fracture that will not heal without further intervention"
"X-p"
"NON-UNION after passing the expected time for union around 6--8 weeks "
"Delayed union: no bridging callus on x-ray 6 weeks after the fracture occurred. Non-union: no evidence of bridging callus 8 weeks after the fracture occurred. "
"Delayed union = absence of fracture healing after 6 months but with healing progression Non-union = no fracture healing after a year"
"The extra time taken by the bone to heal over the normal healing period"
"In fractures with medial comminution we get delayed union"
"Shaft fractures"
"Lack of bridging of at least one cortex at 3 months"
"Delayed union - healing of fracture later than 3 months after injury. non-union - not healed fracture with signs of instability either clinical or radiological later than 6 months after injury, with no healing signs on repetitive x-rays"

"Delayed Union: Union time longer than expected for a metadiaphyseal fracture (6-12 weeks)
Nonunion: no healing after a delay of 6-9 months with pseudo-arthrosis formation"
"Time interval non union zone measurement"
"Xrays at 6 months"
"No sign of clinical or radiological healing by 3 months"
"Failure to obtain clinical and radiographical union at 3-6 months postoperatively or after non operative treatment"
"No signs of bone healing of the fracture in both views within 3 months (delayed) or 6 months (non-union)"
"After six months with no consolidation "
"Delayed union - Absence of bridging bone tissue across the fracture within 6 months of treatment duration. Non-union - Failure of serial radiographs to show progression of healing."
"No progression of healing for 3 months or not healed by 6 months"
"non union: non-healing of the fracture 6 months after treatment "
"delayed- absence of union at 4 months nonunion- absence of healing at 6 months"
"Delayed union: absence of osseous union 12 weeks after fracture/ treatment, however with increasing callus formation on later follow-up X-rays. Non-union: absence of osseous union 20 weeks after fracture/ treatment with increasing bone resorption in fracture gap on follow-up X-rays."
"Delayed union is past 4 months. nonunion is failure of fracture consolidation after 6 months with no progression on radiographs in consecutive months."
"Documented clinically and radiologically"
"Delayed > 6 weeks, non-union 3 months"
"x ray"
"When fracture fails to unite in 3 months will be delayed union and if it fails to unite in 6 months as non union"
"Delayed - absent adequate bridging callus after or persisting symptoms after 6 months; Non-union same after 9 months"
"Persisting open fracture line after 6 months or surgery for non-union"
"see above"
"Nonunion of the humeral head with varus collapse"
"Delayed union: lack of distinctive healing at 3 months. No union: lack of progressive healing at 2 consecutive radiographs at one month intervals after the third month, or lack of conclusive healing at 6 months. "
"No callus or hypertrophic callus and visible fx line after > 3mt"
"delayed: lack of healing at 6 months but with continued progression on xrays nonunion: surgeon opinion that fracture is unlikely to heal without further intervention"
"Sclerosis or collapse of the articular surface of the humeral head post op"
"Delayed- no evidence of union - characterised by persisting fracture gap after 8-10 weeks of treatment- either operative/ conservative"

Fracture malunion

"Loss of reduction and rotational malalignment"
"Fracture has healed, but that it has healed in less than an optimal position"
"The HSA was measured at the intersection of the tangent line of the articular surface with a line parallel to the long axis of the humeral shaft, as previously described. The humeral head height (HHH) was measured on the anteroposterior radiographic image by calculating the vertical distance between the tangent line of the highest point of the humeral head and the greater tuberosity L. Bai, et al Radiological evaluation of reduction loss in unstable proximal humeral fractures treated with locking plates, Orthopaedics & Traumatology: Surgery & Research, Volume 100, Issue 3, May 2014, Pages 217"
"No visible bone healing or x ray and other radiographic studies , after expected period of time appropriate for shoulder fracture"
"Healing of the fracture in bad position, either in varus or with greater tuberosity posterior o high"
"Angulation as above"
"Any healing losing normal humoral head and neck anatomy in any plane."
"Definition?"
"xray and CT scan"
"Union of fragment(s) in nonanatomic position"
"Angulation/displacement"

AO TRAUMA

"Comparison between immediate post-op x-ray and follow up exams and CT scan"
"Non-anatomical tuberosity position with evidence of impingement Varus > 10* (compared to opposite side) "
"As compared to baseline standard radiological views and serial progressive x rays at definite time intervals, see for the malunion"
"1. altered head to tuberosity distance 2. > 20° varus of head neck shaft angle"
"Union of the fracture in less optimal position most common malunion of the GT "
"Classification according Boileau"
"see definitions ahead"
"Healing of any part greater than the deformity to constitute a Neer fragment"
"Fracture union with malposition of the main fragments: - humeral head varus - humeral heal valgus - greater tuberosity above the pole of the humeral head"
"Difficult. see my previous answer"
"Varus or valgus, Anteversion or retroversion of head from normal more than 30 degrees. Migration of tuberosity healed in displaced position."
"Angle of 40-60 degree at fracture site"
"Angulation"
"Improper position of fracture parts after healing"
"Noted by surgeon by inadequate fracture healing position"
"Articular surface in greater than 30 degrees angulation on Y-axilary or grashey AP. NO reference"
"Union in a non-physiological or normal anatomic manner "
"Radiological signs of bone tissue restoration at the fracture site"
"Loss of normal anatomy of the proximal humerus"
"Malunion should be defined according to malreduction using the following criteria according to Schnetzke et al: good quality of reduction/alignment: head-shaft angles of 110-150°, <5mm head-shaft dislocation, and <5mm displacement of the major tubercle . Poor reductiono/malunion: varus <110° or valgus >50°, >5mm head-shaft dislocation, or >5mm displacement of the major tubercle (Schnetzke +, Rate of avascular necrosis after fracture dislocations of the proximal humerus; obere Extremität 2018 · 13:273-278 https://doi.org/10.1007/s11678-018-0452-6)"
"Angulation of the head / neck >10 degrees or displacement of the fracture fragments and healing in this wrong position.> 5mm"
"Fracture healed with displacement or malalignment which exceeds the angulation of 10 deg, tuberosity displacement over 3 mm and surgical neck displacement more than 1 cm."
"Greater than 5 degrees of varus and sagittal plane alignment   greater than 10 degrees of valgus or axial rotation   greater than 5mm shortening"
"Evidence of healing in a malreduced position e.g. 1. If the GT sits higher than the humeral head on the AP view. 2. >20 degrees varus angulation 3. Sagittal plane angulation > 20 degrees 4. Overlap between tuberosities and articular surface"
"Varus or valgus malposition of the head with the shaft (after union), high riding tuberosity, and rotational mal-alignment with improper restoration of version."
"CT angulation/displacement in coronal and sagittal plane "
"Fracture healing in position which can lead in malfunction of shoulder. Valgus, varus malposition of head, malposition of tubercule more than 5mm, rotational malposition."
"More than 5 mm of displacement in tuberosities. Non anatomical reduction of calcar. More than 2mm of gap in fracture"
"Any change in fracture position after surgery, as previously described in Lill's criteria"
"Consolidation of the fracture in a non anatomical position"
"Bone union in a position causing functional impairment"
"Either fracture fragment heals in a position that may affect function afterwards: greater tuberosity over the head or posteriorly, lesser tuberosity medially blocking internal rotation, or the head tilted to anterior or posterior, or in unacceptable valgus."
"Rotation Valgus or varus more than 20 ° with clinical manifestation"
"Healing of the proximal humerus with head-shaft alignment of <110° or >150°, or healing of the displace tuberosity >5mm and <10mm   Schnetzke M, Bockmeyer J, Porschke F, Studier-Fischer S, Gruetzner P-A, Guehring T Quality of Reduction Influences Outcome After Locked-Plate Fixation of Proximal Humeral Type-C Fractures, J Bone Joint Surg Am. 2016;98:1777-85"
"Heilung in Fehlstellung mit daraus resultierender Funktionseinschränkung"
"Displacement of fracture more than 1 cm angulation more than 45 degrees"
"Union in an non-anatomical position with complaints"

"Axis position out of defined ranges   differentiate from non union"
"Bone healing with humeral head in varus, anteversion, retroversion, displaced anteriorly or posteriorly respect the metaphysics and diaphysis"
"According to well established definition"
"Inclination > 10-15 degrees valgus or varus (AO 2007) Scapula Y view: 10-15 degrees apex ant. or post. Tuberosities: Greater T: >10mm Lesser T: >10 mm Contact between diaphysis/ metaphysis and head: <2/3"
"Abnormally aligned fracture fragment either since inception or subsequent development"
"doi: 10.1097/01.blo.0000195679.87258.6e"
"Malaligned fracture healed"
"Often in conservative treatment"
"varus/valgus malposition of tub majus"
"As above"
"Healed with more than 20 degrees difference compared with anatomical angles or tuberosity displacement more than 0,5 cm. Impaction more than 1 cm or translation more than 20%"
"noted before"
"Fracture healed in non-appropriate position "
"Any varus, antecurvatum or recurvatum"
"Healing in varus (neck shaft angle < 120),   Healing with tuberosity displacement more than 5 mm Healing with greater than 50% translation of the humeral shaft "
"Obvious"
"Fracture healing with non-tolerated displacements"
"Neck shaft angle discrepancy compared with the uninjured side more than 15 degrees"
"Fracture healed in unfavorable position"
"X-p"
"Union in a way affecting the function of the whole limb and/ or affecting badly the appearance and shape of the arm"
"More than 3cm shortening or more than 45 degrees of angulation "
"Healing of the fracture in a position corresponding to a >1 part-fracture according to Neer"
"The degree of angulation or translation of the bone fragments with respect to each other"
"Fracture fixed in malposition "
"Head & neck fractures in Varys / valgus"
"Healed fracture in 10 deg varus or 15 deg valgus, or tuberosity 5 mm displaced"
"The fracture healed in the non-anatomical situation, which is visible on x-ray. there can be different grades of malunion"
"Healing in a position deviating 25 degrees from the normal anatomy"
"Radiographic measurements (CCD, AHD, ...)"
"Xrays at 6 months"
"Angulation over 45 deg, tuberosity healing in migrated position "
"Varus or Valgus more than 10 degrees than the normal shoulder, GT impingement "
"Non-union of the fracture with severe displacement   a) Greater tuberosity below the level of the cortex or a side-to-side difference <5 mm (b) No increased varus or valgus (+/-15°) of the head fragment in the anteroposterior view (c) No increased retro- or ante torsion (+/-15°) of   with a severe displacement of the n both views "
"Apparition of atrophic callus"
"Any alteration in shape of proximal humerus geometry with opposite side as template."
"Greater than 20 degrees angulation in any plane, greater than %50 translation in any plane"
"Healing of the fracture in a non-anatomic way"
"Malunion- tuberosity proximal to superior margin of articular surface   varus or valgus position in of humeral head"
"Fracture union in any position which is not anatomical."
"Will be honest, not sure I know how to best define this."
"Unacceptable angulation and displacement"
"Rotation > 25°, axis > 20°"
"X-ray + CT"
"When the humeral head or tuberosities unite in wrong place radiographically"
"Union in an inadequate position with interference in function"
"A score combining intraarticular displacement and varus/valgus displacement"
"see above"

"Malunion in varus"
"Fracture lacking criteria as determined on radiographic parameters described earlier. "
"Healed with > Neer 1-part displacement Jost JSES 22 (4), 2013"
"Varus or valgus >15 deg any greater tuberosity displacement shaft translation >50% shaft width"
"Excessive varus or valgus, loss of offset, shortening, mal rotation or translation of the shaft relative to the humeral head"
"Varus/ valgus malunion, non-reduced intra articular fragments, greater tuberosity malreduction"

Loss of fracture reduction

"Varus or valgus noticed in X rays - altered range of movements"
"Reduction is a surgical procedure to repair a fracture alignment. Loss of achieved alignment is loss of fracture reduction"
"The HSA was measured at the intersection of the tangent line of the articular surface with a line parallel to the long axis of the humeral shaft, as previously described. The humeral head height (HHH) was measured on the anteroposterior radiographic image by calculating the vertical distance between the tangent line of the highest point of the humeral head and the greater tuberosity L. Bai, et al Radiological evaluation of reduction loss in unstable proximal humeral fractures treated with locking plates, Orthopaedics & Traumatology: Surgery & Research, Volume 100, Issue 3, May 2014, Pages 217"
"Change in implant and fracture position after OP "
"Displacement of the fracture with respect to previous radiographic controls"
"Angulation or malpositioning compared with immediate postop XR "
"Losing achieved reduction more than five degrees or displacement more than 5 mm in any plane."
"xray"
"Displacement of fragments which include all previous criteria"
"as above"
"Comparison between immediate post-op x-ray and follow up exams"
"Any change in tuberosity and or head position Change in relationship between head - tuberosity and head- shaft fragments"
"As compared to baseline standard radiological view, and serial progressive x rays at definite time intervals will show the course of loss of fracture reduction"
"Any unintended change in reduction parameters. especially greater tuberosity / lesser tuberosity migration, varus malangulation of head."
"Loss of accepted alignment"
"e.g. non physiological shaft-neck angle or malposition of the tuberosities in comparison to postoperative imaging"
"Tricky, I guess if any loss of reduction is present comparing the two 2 x-rays"
"As above"
"Change of the position of the bone fragments on the control X-rays compare to the immediate intraoperative X-ray control"
"Any change that potentially alters outcome"
"Change in xray parameters from intended position after internal fixation "
"Displacement in fracture fragments that were hold previously by any implant"
"Change in the fracture position "
"Change in accurate position of the bony pieces after final xrays have been taken"
"A change in fracture position in post op xrays"
"Change in reduction from intra op XR to current. NO reference"
"Loss of initial acceptable position of fracture"
"Displacement of bone fragments in the postoperative period or after closed fracture reposition"
"Displacement between the fracture lines , virus valgus and rotation"
"According to criteria defining malreduction and malunion, I suggest that these criteria are also used for definition of loss of fracture reduction in comparison between postop and follow up x-rays."
"Change in the post position in the follow up xrays "
"Change of the fragment position and alignment compared to the immediate postoperative situation detected with x-ray or CT scan"
"Change in position of the fracture after intra-op or immediate postop imaging"
"Where reduction was satisfactory on intraoperative or early postoperative imaging but has subsequently deteriorated beyond the standards described above."

"Secondary loss of reduction in serial post op follow up X-rays in either length, rotation or alignment."
"CT angulation/displacement in coronal and sagittal plane"
"It is difficult to define the exact angle of loss of fracture reduction"
"Dislocation of fragments immediately to 1 month after surgery or conservative treatment"
"Any displacement from a well reduced fracture that fits criteria of malreduction."
"Any change in fracture position after surgery, as previously described in Lill's criteria"
"Loss of initial rehabilitation of the fracture leading to non-union or malunion"
"Change in position of bone fragments after manipulation and internal fixation"
"Fracture fragments are found in a different position in two consecutive x-rays."
"If the construct do not stay as it was intended and documented intraoperatively or shortly after surgery"
"Secondary displacement of the head-shaft alignment or secondary displacement of the tuberosity >5mm"
"Jede Stellungsänderung der Fraktur im Vergleich zum intraoperativen Befund."
"Change in alignment of fracture when comparison made to intra-operative radiographs"
"A change in position of the humeral head or fragments during follow up compared to final operative Images/positions"
"Loss of intraoperative fracture / bone position indirect signs: implant loosening etc. "
"Changes of the position of the bone fragments respect post-op Xray check"
"Any change to post-operative X-ray no matter the size or location. "
"The limits for malunion, however calculated from the postop. radiograph. For LCP: Clear evidence of pull-out of plate or cut-out for head-screw fixation except of penetration due to 'sinking' or avascular necrosis of the humeral head."
"Loss of alignment of fracture fragment on subsequent xray"
"doi: 10.1097/BOT.0000000000000252."
"Secondary displacement of the fracture after treatment"
"After plating and nailing"
"Difference to postop X-ray > 10° in axis, shortening"
"As compared with the first x ray in conservatively treated patients. or loss of reduction as compared with the intra operative fluoroscopy"
"As above"
"Loss of 15 or more angulation in relation to the initial immediate X ray. Must be with the same exposition and subject placement and positioning to the X ray beam"
"as it relates to OC penetration"
"Loss of achieved fragment position "
"When the original fracture reduction is lost compared to the original post operative x rays"
"Change in position of fracture on subsequent postoperative xrays."
"Obvious"
"Displacement of fracture fragments in xray controls"
"Neck shaft angle change compared with the immediate postoperative x-ray more than 15 degrees "
"x"
"Primary: >5mm dislocation of joint-forming fragments Secondary: same, but not on primary postop x-rays"
"X-p"
"Significant change from the initial fracture reduction after fixation with or without external event"
"Change in position of the fracture fragments significant enough to impair shoulder function after initial satisfactory treatment. "
"Displacement of fracture reduction in the follow-up"
"The percentage of displacement of a fracture after its initial reduction"
"In mainly osteoporotic cases we get loss of reduction"
"Displacement "
"Any change in fracture position between immediately postop and final follow-up"
"Migration of the fracture fragments fixed during surgical procedure postoperatively visible on x-ray"
"Difference in position between intra/postop x-ray and further follow up x-ray"
"Descriptive"
"Change in fracture alignment on sequential Xrays."
"Movement from the position achieved during surgery"
"Significant change in fracture alignment between follow up radiographs"
"Secondary fracture displacement after initial good quality of fracture reduction / Secondary displacement of the fracture after satisfactory or unsatisfactory Quality of fracture reduction >5° difference of the CCD angle/> 5 mm tuberosity displacement"

"Screws pull out or broken material"
"Change in position of fracture fragments - head, tuberosities or shaft when compared to immediate post-operative image."
"Greater than 20 degrees angulation in any plane, greater than %50 translation in any plane"
"Fragments mobilization "
"Failure of current treatment to maintain alignment"
"Secondary displacement of fragments on postoperative follow-up X-rays as compared to the final documentation after fracture reduction (and fixation, if applicable)"
"Shift of fracture alignment of more than 5mm in any direction after conclusion of surgery"
"Primary or secondary"
"x ray"
"Increase/ decrease in varus, valgus, retroversion flexion, extension in subsequent radiographic picture as compared to before"
"Change in the fragment relative position, from a previous radiographic control, in an x-ray performed in the same position and conditions"
"A score combining intraarticular displacement and varus/valgus displacement"
"see above"
"Cut out of fracture fragments"
"Fracture that no longer meets criteria for fracture reduction established intraoperatively. "
"Measured in part-criteria according to Neer"
"Change in alignment after surgery"
"Progressive loss of reduction on serial x rays"
"Loss of anatomical reduction in post-operative follow up period, which was not evident in immediate post-operative X-rays loss of initial reduction achieved after closed reduction in conservatively managed fractures"

AO TRAUMA

Suitable timeline for documenting osteochondral events	n (%)
**… in joint-preserving fracture fixation?**	
6 months	44 (25%)
12 months	61 (35%)
24 months	40 (23%)
Lifelong until implant removal	19 (11%)
Other time period†	9 (5%)
**… in non-operative treatment?**	
6 months	55 (32%)
12 months	67 (39%)
24 months	42 (24%)
Other time period‡	9 (5%)

† Other time period in joint-preserving fracture fixation:
"3 months"
"3 months"
"Necrosis can occur at any time"
"I suggest 12 months, but if posttraumatic OA should be also taken into account a lifelong follow up will be required for definition"
"Until fracture healing, 12 months for head necrosis (some cases may occur later but we should select a control point)"
"18 months"
"3mt 12mt 24mt"
"up to 8 years because of the omarthrosis/humeral head necrosis which can be diagnosed late"
"lifelong"

‡ Other time period in non-operative treatment:
"3-4 months "
"5 years"
"I suggest 12 months, but if posttraumatic OA should be also taken into account a lifelong follow up will be required for definition"
"Until fracture healing, 12 months for head necrosis (some cases may occur later but we should select a control point)"
"18 months"
"3mt 12mt 24mt"
"up to 8 years because of the omarthrosis/humeral head necrosis which can be diagnosed late"
"lifelong"
"lifelong"

AO TRAUMA

Postoperative local events: Shoulder instability

For which treatment option(s) should shoulder instability be documented?	N	non-OP n	IM n	Plate n	Other n	None n
	172	139	140	141	137	23
		81%	81%	82%	80%	13%

Non-OP = non-operative; IM= Intramedullary nailing; Other = other treatment options

Definition: symptomatic shoulder associated with loss of alignment of the articulating surface of the humeral component with the articulating surface of its joint partner.

Specifications :

- Subluxation : non arm position-dependent eccentric misalignment with residual contact.

- Dislocation : non arm position-dependent complete loss of contact of the articulating surfaces.

- Dynamic instability : arm position-dependent loss of contact of the articulating surfaces apparent on physical examination and/or visible on functional radiographs (horizontal flexion/extension view in 90° of abduction and true AP view in 60° of abduction).

97% (140/145) of respondents agreed about with this definition, specifications and terminology

Suitable timeline for documenting shoulder instability	n (%)
**… in joint-preserving fracture fixation?**	
6 months	79 (53%)
12 months	41 (28%)
24 months	10 (7%)
Lifelong until implant removal	11 (7%)
Other time period†	7 (5%)
**… in non-operative treatment?**	
6 months	83 (56%)
12 months	44 (30%)
24 months	13 (9%)
Other time period‡	7 (5%)

† Other time period in joint-preserving fracture fixation:
"1 month"
"3 months"
"3-4 months" (2)
"postoperative to six months"
"Until fracture healing, when the patient obtained the maximum expected ROM"
"18 months"

‡ Other time period in non-operative treatment:
"1 month" (2)
"Start of treatment till three months"
"3-4 months"
"Until fracture healing, when the patient obtained the maximum expected ROM"
"18 months"
"lifelong"

Comments and suggestions

"it is an issue in semi-total or RSA treatment more than after joint preserving surgery"

"at 3 months"

"Usually in non-operative cases , we get subluxation , proximal migration of GT , delayed union , nonunion, loss of function"

Postoperative local events: Peripheral neurological events

For which treatment option(s) should peripheral neurological events be documented?	N	non-OP n	IM n	Plate n	Other n	None n
	173	157	170	170	163	1
		91%	98%	98%	94%	1%

Non-OP = non-operative; IM= Intramedullary nailing; Other = other treatment options

Definition : Events resulting from peripheral neurological injury at the fracture site, which is associated with sensory and/or motor and/or autonomic disturbance

Specifications :

Sensory and/or motor disturbance: Affected nerve(s)

- Cervical or brachial plexus

- Branch neuropathy (suprascapular, musculocutaneous, median, ulnar, radial, axillary, dorsal scapular, long thoracic, spinal accessory, thoracodorsal, cutaneous nerves of arm and forearm).

Autonomic disturbance: Complex regional pain syndrome (CRPS)

An optional injury classification can be documented via neurologist according to Seddon* (i.e. neurapraxia, axonotmesis, neurotmesis) and/or Birch** (degenerative, short conduction block, prolonged condition block)

** Seddon H. (1942) A classification of nerve injuries. British Medical Journal, 2(4260): 237-239.*

*** Birch R. (201 0) Clinical aspects of nerve injury, in Surgica/ disorders of the peripheral nerves. Springer. 145-190.*

Period of Observation: 3 months

97% (165/170) of respondents agreed about with this definition, specifications and terminology

Comments and suggestions

"Regarding regional anesthesia"

"Period of observation can be more than 3 months"

"I would recommend to have a longer period of observation, also 2 years"

"Extending time to 6 or 12 months as these evolve"

"Very difficult to diagnose CRPS type II before fracture healing. CRPS should be classified apart. Neurological unfavorable events beside CRPS are exceptionally caused by non-operative treatment."

"How can we differentiate between axonotmesis and neurotmesis if we do not explore surgically the nerves"

"Maybe the period for observation of CRPS should be extended to 6 months."

"Electromyogram after 3 weeks postoperative followed by a minimum of 6 months."

"It should be documented whether the injury is post-fracture, or preoperatively, or postoperatively."

AO TRAUMA

"Also for 12 months (at least), i.e. during observation period (CRPS!)"

"Observation period should be 12 months for Brachal plexus injuries, and 6 -12 months for branch neuropathy"

"CRPS may be a social construction. It's a theory that posits an as yet elusive pathophysiology in people with disproportionate pain and limitations. Mounting evidence suggests that symptoms and limitations are accounted for by stress, distress, and effectiveness of coping strategies without the need to posit an elusive pathophysiological explanation, nervous system or otherwise. "

"Newer methods of documenting changes in blood flow to a peripheral nerve, MRI neurography can also be considered, which supposedly claim to be more accurate."

"Until palsy improves May take longer in plexus injury"

"Maybe we could evaluate clavicle nerves as well"

"Period of observation should be 6 to 12 months"

"Consider type of anaesthesia (regional!) "

Postoperative local events: Vascular events

For which treatment option(s) should vascular events be documented?	N	non-OP n	IM n	Plate n	Other n	None n
	173	153	169	169	163	2
		88%	98%	98%	94%	1%

Non-OP = non-operative; IM= Intramedullary nailing; Other = other treatment options

Definition : Events involving laceration, avulsion, contusion, puncture or crush injury to an artery, vein or microvascularity at the surgical site

Specifications :

- Hematoma which requires evacuation by needle or surgery
- Superficial and deep thrombosis at the involved extremity
- Ischemia of the involved extremity which requires additional intervention

Observation period (timeline) : 30 days

96% (163/170) of respondents agreed about with this definition, specifications and terminology

Comments and suggestions

"add vascular section and/or hemorrhage"

"If the definition includes 'surgical site' Venous Thrombosis should be categorized apart."

"One should exclude harm to the cephalic vein intraoperatively as this occurs very commonly."

"Non Op also will need if the fracture displaces and a new vascular injury is caused"

"See previous comments "

"In definition we can add ---- at the surgical site / injuries occurring as a result of primary injury / sequelae of conservative treatment"

"Not all events have the same importance: an injury to the axillary artery might be life-threating while an hematoma due to anticoagulant medication, can be a mild adverse event. The repercussions, the timing for diagnose and treatment are completely different. I would somehow consider the level of importance."

"also for total observation period, at least 3 months"

"Aneurysm "

"ignore the haematoma bit"

"Surgery for hematoma not 'required'. Very discretionary and preference-sensitive. Vein thrombosis rare. Strict diagnostic criteria needed. "

"Could likely exclude cephalic vein injury from surgical complications."

"hematoma is not always a real vascular event ischemia is one possible vascular disturbance problem, but more interesting would be vascular injuries of arteries around the shoulder"

"Please add the rare event of vascular injury requiring surgical repair such as axillary artery injury - can occur from a medial spike of the humeral shaft"

"I would not include hematomas in this condition"

Postoperative local events: Surgical Site Infections (SSI)

Definition and specifications adapted from the 2008 Centers for Disease Control and Prevention (CDC) definition*

Superficial Incisional Surgical Site Infections: Infections involving only the skin and subcutaneous tissue of the incision

Deep Surgical Site Infections (Incisional AND Organ/Space): Infections involving any part of the anatomy (e.g. fascia, muscle, organs and spaces) other than the skin and subcutaneous tissue of the incision

Specifications: early (< 3 months) / low grade (3-24 months) infections*

Period of observation: 24 months
(note: despite 12 months according to CDC definition when implant in place)

** Horan TC, Andrus M, Dudeck MA, (2008) CDC!NHSN surveillance definition of health care-associated infection and criteria for specific types of infections in the acute care setting. Am J Infect Contra/, 36: 309-332*

93% (160/172) of respondents agreed about with this definition, specifications, terminology and timeline for all operative treatment options

Infections in non-operative treatment

45% (78/172) of respondents agreed that local infections should also be documented in non-operative treatment

How could we define infection in this context?

"Superficial Infections: Infections involving only the skin and subcutaneous tissue of the shoulder - Deep Infections: Infections involving any part of the anatomy (e.g. fascia, muscle, organs and spaces) other than the skin and subcutaneous tissue of the incision"
"Pain, inability to perform exercise, standard, signs of inflammation, support of serological tests,, xrays , ultrasound and MRI"
"Superficial for those infections, with only involve skin, no collections only antibiotic treatment Deep infection with collections , and need surgical and antibiotic treatment"
"Appearance of typical signs of infection: redness ,inflammation ,pain not compatible with fracture, fever and alterations in blood test"
"Skin reaction requiring medication"
"May be there might be small contaminated wound in the nonoperatively managed fracture site."
"We have patients who have fracture without surgical intervention and have infection for example after pneumonia "
"Brace wear could break skin and cause some local infection."
"Cellulitis or infection of local hematoma"
"Any soft tissue injury when systemically or locally get infected in patients with low immunity "
"Could be due to infected Haematoma. Redness, raised temperature of the part with altered blood parameters."

AO TRAUMA

"Proven infections that only respond to systemic &/or surgical treatment"
"Localised hematoma that got infected later on i.e. a week or later, should also be documented. Soft tissue damage or skin damage may lead to infection or open fracture, in patients where we do non operative treatment for a fracture so it's documentation is important."
"Any redness and swelling to be documented at site"
"Redness, warmth and tenderness treated with antibiotics"
"Any infection of soft tissue perifracturative "
"Purulent-inflammatory process in the area of damage to the shoulder, which arose and is associated with the above-mentioned damage. "
"Fracture Hematoma infection. Diabetic patient "
"I suggest to document trauma related soft tissue defects in such cases and also to include open fractures as primarily infected fractures."
"Infected hematoma or septic arthritis of joint"
"Cellulitis, or skin violation with subsequent cellulitis and/or drainage"
"Early : less than three months"
"Clinical signs and symptoms on infection like warmth, redness, pus discharge as well as leucocytosis and raised inflammatory markers like ESR, CRP, S Procalcitonin etc"
"Infection attributable to any external form of immobilization (due to skin breakdown, etc.)"
"Infection after open fractures treated non-operative and local infection in superficial or deep traumatic wounds which can lead to prolonged fixation or delayed physiotherapy."
"Signs and symptoms of infection, as previously stated."
"There can be infection due to haematoma and swelling in the sense of erysipel"
"Problem: stratification of local infection acquired by chance and NOT attributable to the fracture and its treatment"
"Infected hematoma. Typical septic arthritis signs. "
"Skin changes and raised local temperature plus abnormal acute phase reactants"
"Post Non Operative local Superficial infection due to the application of the device 30 days"
"Clinical history and examination AND resolution with antibiotics"
"Although very uncommon I have seen infection after conservative treatment of fractures"
"if local infection develops due to treatment (non-op) e.g. in axilla due to poor hygiene "
"Skin changes requiring antibiotics."
"Certain inmobilization can cause skin abrasions and these can get infected"
"Infections can be acute or delayed, we currently have a philadelphia consensus as a guide for the diagnosis and management of infections"
"Infection around the area of trauma or fracture"
"For me there is no difference between a local infection and a superficial surgical site infection, so both should be documented"
"1. infection can be classified with superficial and deep infection . In deep infection implant in situ or without implant. 2.with implant - we can define weather implant stable or lose 3.then condition of fracture , weather uniting or non union"
"Discharging wound"
"Infection in the skin in the region of fracture: Tender, swollen, red skin with or without wound (not surgical, but contusion or insite-out)"
"Abrasions or skin lesions which were preexisting before commencement of treatment and then got infected. Lesions caused due to improper or tight application of Sling "
"skin observation aspiration if needed"
"Superficial Incisional Surgical Site Infections: Infections involving only the skin and subcutaneous tissue of the incision - Deep Surgical Site Infections (Incisional AND Organ/Space): Infections involving any part of the anatomy (e.g. fascia, muscle, organs and spaces) other than the skin and subcutaneous tissue of the incision within 3 months after fracture"
"If fracture hematoma infected by any hematogenous contamination "
"Clinical evidence of infection in axilla, fracture hematoma with the need for active management in form of antibiotics/surgical drainage."
"Culture positive or requires operative I and D"
"Infection of the hematoma or of the joint or of the bone in a patient with humerus fracture"
"Any infection requiring antibiotic treatment or surgical intervention"
"Superficial or deep infection or infection associated with implants"
"Infections around the fracture site and infections caused by bandage"

"Conservative management can cause allergies related to material of treatment with conservative means like a sling/ plaster/ cotton/ cloth. This may also cause axillary infections which although will not be SSI but may be important to study the impact and risks of non conservative treatment in the study"
"Only in cases of associated wounds, whose treatment or evolution might impact rehabilitation, immobilization or function"
"Any skin or deep infection, for example, from an abrasion that requires oral or IV antibiotics"

Comments and suggestions
"I'd suggest 4 weeks period for early infection; 4-10 weeks for delayed and more than 10 weeks for late. Trampuz A, Zimmerli W. Diagnosis and treatment of infections associated with fracture-fixation devices. Injury 2006;37(Suppl 2):S59-66. Willenegger H, Roth B. Treatment tactics and late results in early infection following osteosynthesis. Unfallchirurgie 1986;12:241-6."

"Early infection should be less than 6 weeks"

"Complications in conservative treatment should be addressed as external device complications. FRI criteria may be included."

"Usually SSI is defined as infection within 30 days, therefore early can be specified within 30 days late within 30 days to 03 months and low grade 03 to 24 months"

"max 12 months deep infection can only be detected on Revision Operation, that means that this is obligatory. Otherwise it will be superficial with only AB"

"to be discussed: 6 weeks limit for early infection risk of maluse of the definition 'superficial SSI' by surgeons!"

"Time line should be - till implant is in place and local tissue status at time of removal of implant for various reasons"

"There is no way to distinguish superficial and deep infections."

"early infections < 45 days in orthopedics rarely to speak for superficial site infection "

"Time period of deep SSI should be increased, up to 36 months."

"Use Pro Implant Infection definition, much more appropriate or as published in Injury last year as consesus of experts ! Not this one !"

"The issue of SSI is data capture as length of stay is now very short in hospitals. The secondary issue is how would one capture community data, as once discharged the patient would most likely be seen by their local medical officer."

"Since bacterial flora is different in shoulder may involve all non unions too"

"k"

"Local infections should be documented if it is an open proximal humerus fracture "

"I think 12 months is sufficient for infection"

"Differentiation between early and late infections according to specific definitions used for operative fracture treatment (i.e. early = less than 6 weeks after intervention)"

Postoperative local events: Superficial soft tissue events

For which treatment option(s) should superficial soft tissue events be documented?	N	non-OP n	IM n	Plate n	Other n	None n
	173	137	169	170	166	2
		79%	98%	98%	96%	1%

Non-OP = non-operative; IM= Intramedullary nailing; Other = other treatment options

Definition : Events affecting the superficial soft tissues (i.e. skin and subcutaneous tissue) at and around the fracture site/wound that do not affect deep soft tissues (i.e. fascia, muscle, articular capsule) and that require additional treatment

Specifications and observation period (timeline) :

- Early events over 30 days: edema; emphysema; delayed wound healing; hypersensitivity reaction; skin necrosis; skin bulla; other

- Late events over 6 months: hypertrophic scar and keloid

98% (165/168) of respondents agreed about with this terminology, definition, specifications and timeline

Comments and suggestions

"Non-op treatment could have extensive hematoma that leads to some scar formation."

"We must document however any superficial bruising (infected or not), which cause a delay or even no operation."

"Ulcers/pressure sores/skin necrosis caused by immobility from splints/slings etc are consequence of treatment choices by the surgeon"

"Keloid develops sometimes with no infection"

"The timeline for early events should include events appearing right after the trauma: this means we should consider open fractures as a superficial soft tissue event. Unless open fractures are registered in another complication definition."

"Cave superficial vs. deep infection scar quality has to be registered independent from infection"

"Too inclusive. Edema is routine."

AO TRAUMA

Postoperative local events: Deep soft tissue events

For which treatment option(s) should deep soft tissue events be documented?	N	non-OP n	IM n	Plate n	Other n	None n
	172	134	170	171	166	0
		78%	99%	99%	97%	0%

Non-OP = non-operative; IM= Intramedullary nailing; Other = other treatment options

Definition : Events affecting the deep soft tissues (i.e. fascia, muscle, articular capsule), except infections

Specifications :

- affecting the subacromial space (impingement, adhesion, ...)
- affecting the biceps
- affecting the capsule (shoulder stiffness, ..)
- affecting the rotator cuff : events affecting the anatomical and functional integrity of the rotator cuff including one of the following muscles and tendons: subscapularis, supraspinatus, infraspinatus, teres minor
- affecting the deltoid

Observation period (timeline): 12 months

99% (163/164) of respondents agreed about with this definition, specifications and terminology

Suitable timeline for documenting deep soft tissue events	n (%)
**… in joint-preserving fracture fixation?**	
6 months	53 (31%)
12 months	72 (42%)
24 months	17 (10%)
Lifelong until implant removal	22 (13%)
Other time period†	7 (4%)
**… in non-operative treatment?**	
6 months	77 (47%)
12 months	62 (38%)
24 months	15 (9%)
Other time period‡	10 (6%)

† Other time period in joint-preserving fracture fixation:
"1st to 4 weeks "
"6 weeks"
"<3 months, keeping in mind the occurrence of infection as one of the soft tissue events"
"Until healing"
"Within three weeks "
"1 and 3 months after fracture"
"18 months"

‡ Other time period in non-operative treatment:
"3 to 4 weeks"
"3 months"
"None"
"Until bone healing "
"N/a"
"1 and 3 months after fracture"
"5 years"
"Until healing"
"18 months"
"Likely not needed for non-operative"

Comments and suggestions

"Need for ultrasound to evaluate rotator cuff involvement"

"I suggest including and documenting deep soft tissue infections also in non-operative treatment as maybe initial open fractures are treated without fixation."

"Ultrasound imaging or MRI at 6 months postoperatively if persistent active muscle deficiency"

"To be such specifications of deep tissues are artificial - how can we surely know? classification operative vs. conservative: from a clinical point of view of course certain details such as infection after nonoperative treatment can be finally judged much earlier than for operative treatment; on the other hand from a systematic approach, end timepoints should be the same for the comparison of treatments"

"Adhesive capsulitis or post-traumatic stiff shoulder rather 24 months"

"Coding for Involvement of a region with sub coding for the expected specifications with extra addition if due to additional effect of infection"

"C acne is the problem bacteria in the shoulder Clinical signs may not be present"

"With non-operative treatment 3 months"

"Maybe 3 months will be more suitable for that event"
