# Supplementary material for: Core set of unfavorable events of proximal humerus fracture treatment defined by an international Delphi consensus process
Source: BMC Musculoskelet Disord. 2021 Nov 30;22:1002. doi: 10.1186/s12891-021-04887-1 (PMC8630858; doi:10.1186/s12891-021-04887-1)
Supplement: Supplementary file 6 — Additional file 6. PHF Core Event Set v1.0 - Delphi 02 survey results – June 2020. [file 12891_2021_4887_MOESM6_ESM.pdf]

Supplementary file 6

**Article title**	Core set of unfavorable events of proximal humerus fracture treatment defined by an international Delphi consensus process
**Journal name**	BMC Musculoskeletal Disorders
**Author names**	Audigé L, Brorson S, Durchholz H, Lambert S, Moro F, PHF CES Consensus Panel, Joeris A
**Affiliation**	Schulthess Klinik, CH-8008 Zurich, Switzerland
**E-mail address**	laurent.audige@kws.ch

PHF Core Event Set v1.0

Core list of unfavorable events of proximal humerus fracture treatment (PHF)

Delphi 02 survey results

June 2020

Methods and participants

Response to the second survey

143 surgeons (44% of 327) responded to the second survey, of which 128 (90%) completed the survey. Other participants responded only partially.

AO TRAUMA

General comments about the survey and project
"Well done"
"An important work that will help authors to report trials, clinical- and radiographic results in a better way in the future. Thank you to be a part of the survey."
"Thank-you"
"Looks good. When will it come out"
"Great! thank you for the opportunity to participate!"
"All the best!"
"Thank you very much for allowing me to participate in this work. I think it is very useful to be able to establish evaluation and control criteria that allow us to improve the treatment and follow-up of fracture treatment in this bone segment. Since there are many variables involved and very different monitoring criteria."
"Well done !!!"
"No, very good work, Best Regards, FL."
"Thank you for this opportunity to contribute to this challenging topic."
"Thank you for the opportunity to help. Hope my experience can contribute. Good luck."
"Look forward to the collated responses of the survey and the agreed points. Thanks for the opportunity to be a part of the survey, it was educative too."
"Very comprehensive consensus. You have a lot of work. Congratulations. "
"Great work, congratulations"
"Thanks"
"It was a good idea to try to classify this types of problems"
"Great work. "
"Congratulations for your efforts to structure the approach to PHfx treatment. Looking forward to a uniform database. "
"Good job"
"In general very good to get consensus on adverse events/complications. be aware to define too many items. This will turn out to be unusefull in future research if incidence is low of certain small complications. it will be very difficult to do any statistics."
"Evaluation criteria of postreaumatic stiffness."
"Thank you for these information "
"Thanks for carrying out this whole lot amount of work. I am looking forward to the consensus."
"Nice work!"
"MY ONLY WISH IS THAT THE RESULT OF THE CONSENSUS SHOULD BE AS PRECISE AND AS SHORT AS POSSIBLE, SO THAT IT SENDS A CLEAR MESSAGE EVEN TO THE BEGINNERS AND IS UTILISED AND BELIEVED MORE AND MORE. THANKS"
"No, an excellent and well done survey would be extremely helpful in evaluating and managing these difficult injuries especially in our elderly. PHF still remains a unsolved fracture. "
"Appreciations for the hard working from steering committees."

AO TRAUMA

Published Core Event Set in shoulder arthroplasty (SA)

We present below two important publications recently completed regarding shoulder arthroplasty (SA) in the context of a similar international consensus process with shoulder surgeons. It covers the documentation of core sets of adverse events in one, and of radiological monitoring parameters in the other.<br><br>Our steering committee recommends that any PHF treated by SA should be documented on the basis of this consensus work. The present survey therefore focuses only on fracture treatment whether by surgery or non-operative management.<br><br>Note: we invite you to review these papers and make any comments or suggestions you may find appropriate in the context of PHF treatment.<br><br>*Audigé L., Schwyzer H.-K., SA CES Consensus Panel, Durchholz H. Core set of unfavorable events of shoulder arthroplasty: an international Delphi consensus process. Journal of Shoulder and Elbow Surgery (open access)*<br>*doi: 10.1016/j.jse.2019.07.021*<br><br>*Durchholz H., Salomonsson B., Moroder P., Lambert S., Page R., Audigé L. on behalf of the SA Monitoring Steering Group, Core set of radiological parameters for shoulder arthroplasty monitoring: criteria defined by an international Delphi consensus process, Journal of Bone and Joint Surgery Open Access, 2019. 4(4): p. e0025*<br>*doi: 10.2106/JBJS.OA.19.00025*

98% (138/141) agreed about that recommendation

Comments and suggestions from participants who disagreed

"Documented where and what type of documentation - are you referring to the unfavourable events in Audigé paper?"

"I disagree with the definition of infection and the times used to classify in early, low grade or late. This condition should be adequate to the times used for prosthesis salvage, and maybe 4-6 week would be a better control point to separate early and intermediate infections."

Comments and suggestions from participants who agreed

"All parameters were taken in consideration "

"Concerning paper 1 by Audige et al; table IV: Shoulder stiffness. With regard to shoulder motion after fracture surgery the limits for GH motion flexion and abduction with fixed scapula is too narrow to my experience, as less motion (esp. for elderly) is common and do not cause serious problems for ADL."

**Shoulder stiffness**<br>Postoperative restriction in passive shoulder motion diagnosed in ≥ 2 of the motion planes of flexion, abduction, and external fixation in 0° of abduction.<br>Motion restriction is assessed separately for each plane according to the following criteria:<br>**Flexion**: total motion ≤ 90° or glenohumeral motion (fixed scapula) ≤ 80°<br>**Abduction**: total motion ≤ 80° or glenohumeral motion (fixed scapula) ≤ 60°<br>**External rotation in 0° of abduction**: glenohumeral (fixed scapula) motion ≤ 20° (or, for anatomic shoulder arthroplasty only, no more than 50% of the contralateral-side value)<br>For the core set, only shoulder stiffness occurring within 12 months after shoulder arthroplasty is considered

"There is a view that is regions where there is a good primary care set up then patients with solid prostheses do not require any follow-up until, they have an increase in symptoms. Regular follow up is thought to not provide any benefit, as repeated x-rays may not prevent the revision and timely revision may have no better success that revision at failure. Defining the key parameter(s) that is associated with early revision may be useful, Whilst we define the key parameters, then I agree with the recommendations."

AO TRAUMA

"The arthroplasty monitoring in several details might differ from fx treatment undertaken by ORIF; e.g. with regard to duration of monitoring etc."

"It may be possible to make fewer radiographs after arthroplasty. It likely is. More research is needed. "

"Very happy to agree on a consensus, though I do not do shoulder arthroplasty"

"There is need for higher Level of Evidence"

"The suggested recommendations are standard and take into consideration the implant, what it does to the bone and what the bone is doing to it."

"I find those two papers as a useful tool in everyday work and specially science. Especially the first article where the conclusion were accepted after only 2 rounds"

"Excellent"

"As these are recommendations, their true value and impact will be dependent on frequent use, comparability and citation frequency."

"Usefull"

"The plan of treatment for PHF should place SA in the frame too."

"If limited to fractures treated by early SA"

"Wasn't sure about osteochondral events referring to the chondral parts in intra-operative events in the first paper"

Intraoperative events (1)

The intraoperative period is defined as the time interval between skin incision and skin closure. When the fracture is reduced under anaethesia in the context of non-operative management, an equivalent "fracture reduction" period is considered as the time interval between the patient entered the operating room (OR) and the time the patient exited the OR.

91% (128/140) agreed with this added definition

Comments and suggestions from participants who disagreed

"The time from starting the reduction procedure to the end of immobilisation in a shoulder bandage. "

"This will make all events due to anesthetic induction 'intraoperative events' in the case of closed manipulation and at the same time 'non-intraoperative events' in case of any skin cutting."

"How significant would this time however be? Certain instances in conservative treatment we may not go in for a closed reduction per se."

"Fracture reduction period should be considered from the beginning of any closed manipulation and not from the entrance in the OR. Otherwise intraoperative event would also need to be defined as starting with the entrance of the patient in the OR."

"Start anaesthesia until end of procedure"

"I do not agree to the fracture reduction period as that would include a lot of other things and anaesthesia time. The time when manipulation is started to the time the manipulation is accepted as reduction should be taken as the period. This is because the operative period is also skin to skin time and does not include the preparation time."

"Fracture reduction: time between starting reduction and achieving reduction."

"In closed reduction, the intraoperative time is the manipulating time including fluoroscopy and inmobilization."

"Even in case of operative management (not just nonoperative, as mentioned above), it might be, that a reduction maneuver is performed prior to the skin incision (f.e. in case of a dislocation-type fracture), and it might be, that this reduction maneuver disrupts a preoperative still intact medial hinge (what would be a serious event)! This should also be considered as an intraoperative complication/event. Therefore I would change the above written sentence to: 'When the fracture is reduced under anaesthesia, either in the

context of a non-operative management, or prior to skin incision in case of operative treatment, an equivalent ......"

Comments and suggestions from participants who agreed

"Seems a very good definition of the time periods for any data dictionary."

"I think if all agree we can avoid this"

"In fracture dislocations, especially with surgical neck reduction in OR under anaesthesia is mandatory rather than attempts in Emergency. "

"OR is in this case considered as a place where reduction is performed. What if reduction is done elsewhere? I assume that definition of intra-operative events is accepted (97% agreement. However there is still a problem of events origin during the operation and realised in the postoperative period."

"Good"

"Immediate post-operative period to be also added and to be taken as first 24 hrs after surgery as events in this period can also be due to missed intraoperative period."

Intraoperative events (2)

Changes are proposed regarding intraoperative device and soft tissue events as shown in red in the text below:
note that "screw/bolt perforation" is no longer considered because, once removed intraoperatively after the perforation, it has no consequence to the patient (no event) and no surgeon would ever report it if that occurred occasionally.

Device events

Definition : Events affecting any component of the implanted device or material, or the instrumentation used for their implantation.

Specifications :

- Instrument problem (breakage, failure)
- Implant (breakage, malpositioning, separation)
- ~~Screw / bolt joint perforation~~
- Cementation problem (augmentation)

Soft tissue events

Definition : Events involving only the soft tissue at the treated shoulder

Specifications :

- Skin, muscle, tendon, joint capsule, ligament, labrum
- Blood vessels (bleeding) : bleeding at the surgical site that requires additional intervention or leads to a stop of the operation
- Nerves*: recognized damage of a neurological structure ~~which needs additional surgical intervention~~

* a standard list of potentially affected nerves will only be presented for postoperative neurological events

93% (128/138) agreed with these changes

Comments and suggestions from participants who disagreed

"Perforation of screw is not reported, but may affect integrity and failure-resistance of the fixation construct"

"Do not remove screw/joint perforation. The rest is ok"

"Not to the phrase 'screw perforation': Penetrance of the subchondral bone may be unfavourable for an extracapsular fracture due to bleeding into GH joint, and for the intracapsular C-types possibly due to sinking of the humeral head, although different directions of the screws. The rest i ok."

"I agree except for the screw/bolt perforation"

"Agree with the soft tissue event. Joint perforation should be reported."

"Screw bolt perforation is real and needs to be somewhere as adverse event."

"I think the Screw Joint perforation should remain.   The Soft tissue is OK"

"Primary/intraoperative screw perforations should still be listed. These are 12% complications. Certainly, these perforations should be distinguished from the detected and repaired perforations."

"I guess I would disagree with this change for two reasons: 1. the screw may be through the articular surface and not recognized until later, but this is still an intraoperative problem 2. If the screw perforates, and is shortened, it likely predisposes the construct for failure."

"Screw /bolt joint penetration' does happen intraoperatively, although it is recognized after surgery."

Comments and suggestions from participants who agreed

"Intra and immediate post operative ct scan imaging are much important than the x-ray   I recommend to have them specially with the complex fracture patterns"

"When there are vanishing tuberculi or periprosthetic fractures?"

"I agree to include cementing problems but in cases where alternative augmentation techniques are used such as double plate, or structural graft of fibula or iliac crest should be taken into account"

"Intentional biceps tenodesis post fixation must be mentioned in operating notes."

"Regarding device events, screw joint perforation is important if undetected during the operation and discovered later on. I still this consider as intraoperative event. How to solve that?"

"Good"

"Implant (breakage, malpositioning, separation) - Screw / bolt joint perforation Screw perforation is a distinct problem, different from breakage, malpositioning, separation"

"Inadequate purchase of screw in osteoporotic bone also to be added. Status of Rotator cuff to be added if possible as Shoulder function are dependent on it."

"Additional device event: contaminated or missing component "

AO TRAUMA

Radiological monitoring (1)

Required standard radiographic views

	N=136	
	n	%
True anteroposterior (AP) view in 0° abduction in neutral position	109	80.1
True AP view in 0° abduction in both internal and external rotation	48	35.3
Axillary view in 90° abduction	89	65.4
Y-view *	101	74.3
Other radiological views	13	9.6

* (e.g. if axillary view cannot be obtained due to limited abduction capacity)

Other radiological views

"Bloom and oubata viewWich is a sky view that can be obtained evnen if the patient is immobilised and it will give information about the tubeosities position as well as any ap subluxation of the head"

"True AP of gleno humeral joint"

"Lateral"

"Axial view in 30 abduction in the scapular plane"

"Y view does not replace Axillary view. An axial view shoulde be obtained with a Velpeau view if standard axillary is not achievable. "

"CT-scan"

"Axially view with less than 90 degrees abduction when 90 degrees not obtainable"

"Modified axials can be taken if there is limited abduction. A Y view may still not able to detect angular deformities of the head segment as good as the axial views."

"Mostly AP and Y after 6 weeks. After 3 months IR and R and axial view. these views Arte often not yet possible after 6 weeks"

"Neer I, Neer II"

"Lateral"

"We use the Velpeau view (in lieu of axillary) along with a true Ap and an y-view as our standard three radiographs"

AO TRAUMA

Time points of systematic monitoring of radiological parameters for all patients

Within the first three months

We received 92 valid responses suggesting between one and maximum 6 time points for radiographical images (median 2).

The suggested timing is presented in detail below, however summarized in the following table:

Time point	IntraOP	Immediate PostOP	Week 1	2	3	4	5	6	7	8	9	10	11	12
n	3	32	11	28	11	18	0	68	0	13	1	0	0	27
%	3%	35%	12%	30%	12%	20%	-	74%	-	14%	1%	-	-	29%

The majority of respondents agreed on the 6-week time point. Four respondent mentioned a range, i.e. 4-6 months (1) or 6-8 months (3). Few participants suggested that the timing may differ depending on the fracture type or treatment, or that the type of image may differ depending on the time point:

“Not necessary for non-displaced or those treated operatively”

“No monitoring in minimally displaced fractures”

“Week 1, 2 and 3 only non-OP treatment”; “2 weeks only for non-OP”; “3 weeks only for non-OP”;
“Day 7-10 if treated non-operatively”

“High risk patients prone to failure such as displaced four part fractures in patients with poor bone quality and patients with various fixation, absence of medial calcar support, misplacement or no fixation with calcar support screws should be monitored at 6 weeks”

“Axillary view only intraoperatively and 12 weeks”

Detailed responses

" 4 weeks "

"Immediate post-op, weekly until week 3, week 6, week 12"

"after 2 weeks at the 4 weeks then at 6 weeks"

"6 weeks po"

"The day one post-op. usually the patient is operated under regional anesthesia so it is very hard to obtain the good xray the day of the operation. 15 days post-op, 6 weeks post op and 3 months post op."

"For non-operative displaced fractures with or without closed reduction: after immobilisation and before start of exercises: at 2 weeks. Not necesarry for non-displaced or those treated operatively."

"at post-op top check position at 6 weeks when post-operative pain had resolved and standardised x-rays may be taken"

"0,2, 4 weeks ,8 and 12 weeks"

"Next day of surgery and at two weeks"

"6 WEEKS"

"Post-operative day 1 day 15 day 30 day 60"

"2 weeks postop (before ambulatory fhysical th) 6 weeks post"

"6 weeks"

"post-accident, intraoperatively (fluoroscopy), postoperatively, 6 weeks, 12 weeks (axillary view only intraoperatively and 12 weeks)"

"In addition to the first control in the immediate post op, to evaluate reduction quality and identify possible technical errors. At 30 days closely monitor failures in consolidation, loosening, or loss of reduction caused by rehabilitation, which would help us to adapt post op management and 60 y 90 to control the healing process"

"2/52, 6/52, 12/52"

"1 Week 3 Weeks 6 Weeks 12 weeks"

"First week Then first month   Then after 2 months"
"Post-op; post manipulation 3w 6w "
"inmediate postop   6 weeks"
"Two months post-surgery"
"Intra-op, 6 and 12 weeks"
"2 weeks and 6 weeks in unstable PHF treated non-surgically No monitoring in minimally displaced fractures"
"Occasionally two weeks depending on the patient. One month and two months"
"Immediate Postoperative, 6 weeks"
"after 6 weeks"
"nice and important paper: doi: 10.1007/s00402-015-2368-6   note that true ap view OF THE SHOULDER, needs neutral Rotation OF THE ARM for TRUE AP OF THE PROXIMAL HUMERUS. But external and internal Rotation views may be helpful, because FU-X-rays are never perfect, sometimes done withe the patients arm in a sling, and later with the arm in external rotation"
"At or immediately after (within 24 hours) the primary intervention; At 1/52, 2/52, 3/52 if fracture treated non-operatively All fractures at 6/52"
"1 week   6 weeks"
"Six weeks as protocol.   If there is a complaint of sudden pain or reversal of gained function - radiography may be done per case basis."
"immediately post op. 4 weeks 8 weeks"
"2 weeks 6 weeks"
"Imediaty pos op and 1 month"
"6 weeks."
"Immediately post op 6/52"
"1 week 3 weeks 6-8 weeks 3 months"
"- postoperatively (during first 5 days after intervention) - at 6 weeks   "
"6 w 12 w"
"post-Op   6 weeks post-Op"
"direct postoperative   after 4 to 6 weeks"
"6 weeks"
"6 weeks"
"15 days"
"Yws"
"2 weeks, 6 weeks, 12 weeks"
"After 6 weeks"
"1 week, 3 weeks, 6 weeks"
"1 week 3 weeks 6 weeks "
"6 weeks"
"6week "
"immediate post op, then 6-8 weeks"
"Post OP 2 Weeks 6 Weeks 3 Months"
"1 day 3 weeks"
"6 weeks"
"postop - 6 weeks"
"at 2 and 6 weeks after conservative treatment at 6 weeks after operative treatment"
"postoperative and after 6 weeks"
"2 weeks after intervention in the case that we have only intraoperative image transucer.6 weks p.o."
"2 weeks and 6 weeks"

"1 - Start of ROM exercises (with in 1 week) 2 - at 6weeks 3 - At 12 weeks"

"2 weeks, 4 weeks"

"yes the three poryections above"

"1st week 2nd week 4th week 2nd month 3th month"

"2 weeks, 6 weeks"

"High risk patients prone to failure such as displaced four part fractures in patients with poor bone quality and patients with various fixation, absense of medial calcar support, misplacement or no fixation with calcar support screws should be monitored at 6 weeks "

"postop postop week 6   postop week 12 non-operative week 1 non-operative week 3 non-operative week 6 non-operative week 12"

"Two weeks post op"

"2 and 6 wk"

"after 1 month"

"every 6 weeks"

"6-8 weeks"

"remarks to the above mentioned list: postoperative we perform an true ap and lateral (Y-view) with 0° abduction in neutral position, because an axillary view, and internal and external rotation views are not feasable immediate postop (to to pain, limited range of motion etc.). But after 6 weeks, we perform 4 standardised x-rays: true ap, ap in internal and extrernal rotation and an axillary view: At 6 weeks time, it is possible to perform these 4 views, because range of motion should allow to do that, and the pain has disappeared. These 4 views are important to savely detect or exclude secondary screw perforation (which is the most common adverse event, and what has direct implications: f.e. immediate change of screws to prevent chondral damage on the glenoid cavity)."

"2 weeks 1 month 3 months"

"Immediate post - op 4 wk 8 wk 12 wk"

"Six weeks and three months"

"After surgery, 6 weeks,"

"Immediate post op 3wks post op 6wks post op 9wks post op 12wks post op any time in between, if any abnormality suspected or visualised in any x-ray."

"2, 6 and 12 weeks"

"Postoperative before discharge to home and at first follow up usually at 6 weeks"

"every month."

"day 0 (day of injury) post-operative if operated day 7-10 if treated non-operatively initially day 90 regardless "

"2 weeks 6 weeks"

"Six weeks "

"6 weeks"

"time 0, 3 wk, 6 wk, 3 mo"

"2days postop, 6 weeks"

"6 weeks "

"After 6 weeks"

"Every month"

"intra-op (dead easy to get!) 6/52 3/12"

"6 weeks 3 months"

"6 weeks "

"directly postoperative 2 weeks after operation 6 weeks after operation"

"1 month 2 months"

"X-RAY every month, the first 3 months" "

After three months

	N=135	
	n	%
3 months	104	77
6 months	90	66.7
9 months	18	13.3
12 months / 1 year	106	78.5
2 years	32	23.7
At implant removal (if performed)	60	44.4
Other time point(s)	6	4.4

Other time point(s)
"if significant pain"
"Depending on actual problems"
"Every six weeks until healing"
"when pain, stiffness occurs --> AVN"
"After physiotherapy"
"Further X-rays (at 6 weekly intervals) if union is still progressing and not complete. After documented union, only if symptoms warrant a radiological evaluation "

53% (71/135) agreed that monitoring should be stopped as soon as PHF healing is documented

Radiological parameter(s) (2)

Survey responses

Mentioned radiological parameters included: implant positioning, screw perforation, fracture anatomical reduction, tuberosity healing, bone healing, new fracture, avascular necrosis, glenohumeral joint aligment, and degree of post traumatic arthritis.

<u>Comment from the steering group</u> : A number of parameters were proposed at the initial survey. We considered they were mostly not based on evidence, in particular when considering the threshold values over which outcome is likely to be impaired. In addition they reflected negative or inadequate performance with terms such as "malreduction" or "malunion". In any treatment PHFs may be considered "malreduced" to some extent, and in non-operative management, one would expect some degree of "malunion". What is to be tolerated for each patient is not well defined and some guidelines would be extremely useful; it is not the objective of this project however to develop such guidelines at the current stage, but to develop a documentation system allowing to do so in the future.

Proposed parameter set

The radiological parameters to be monitored include issues of bone shape (related to the patient's anatomy) and bone health :

- Healing*
- Bone resorption / Bone formation*
- Slippage of the head in varus or valgus position leading to a secondary cut out of the screws through the head**
- Tuberosity migration : "any" perceived migration, in comparison to initial radiographs
- Head necrosis*
- New peri-implant fracture (surgical fixation only)

* definitions are proposed for review and agreement in following sections of this survey
** terns "Cutout / Cut-through", "Head collapse" and "Loss of reduction" relates to the same event process / concept and should be defined as such

95% (126/132) agreed with these definitions, specifications and terminology

Comments and suggestions

"Loss of reduction could be of tuberosities and so should be clarified and documented as such. This is obviously different to the loss of humeral Head reduction or collapse."

"Although YES, there is an important difference for footnote ** according to my experience: 1) Cutout, loss of reduction is due failure of stability/ osteosynthesis, while 2) Cut-through - penetration along screw-axis or head collapse ('sinking') is due to bone-healing or biology /bone necrosis"

"rather than slippage - is this not loss of humeral head reduction ? slippage of head is not a clean term rather than head necrosis - is this not humeral head avascular necrosis"

"position osteosynthetic material?"

"healing- consolidation of three out of four cortices usually is sufficient for painless movement"

"'cutout' may differ from 'cut through' with regard to its origin, i.e. event process the fact of stated effective migration depends on the accuracy of the underlying measurement technique "

"we cannot Judge on healing directly, no callus head at the head or tuberosity. We Judge on 'FAILURE'!"

"terns 'Cutout / Cut-through', 'Head collapse' and 'Loss of reduction' relates to the same event process / concept and should be defined as such they are not always related do the same event. You can have loss of reduction and head collapse without cut through or out and vice versa"

"Term bone resorption for me is not referred just to humeral head, but also too tuberosities. If not, it should be specially clarified in tuberosity line."

"Excellent"

"I would not use the term 'slippage' secondary cut out is a separate event the term 'migration' seems better than slippage but could also be replaced by displacement more detailed description of osteonecrosis (extent, location) "

"'Slippage' and 'migration' imply a dynamic process of bone position change. The reality is we care about any position of fragments that is not anatomic. How this relates to clinical outcome can be open to later discussion, but you must start with an objective measure of bone position. "

"there can be a slippage in varus without screw perforation, therefore I would separate these two points."

"I would add greater tuberosity resorption"

"all these parameters are subject to subjective assessment and unless defined quite useless for research purposes or analysis of registry data"

"I've always thought that the aim of an operative reduction should not be to get it absolutely anatomical as there may require additional dissection etc but to aim to get it to at least the kind of position that one would accept non-operative treatment if the patient presented with that fracture"

"x-ray: true scapular AP view with humerus neutral rotation plus internal rotation view to measure interval change of 1. Neck shaft angle 2. Humeral head high 3. G tuberosity migration referred to top of plate 4. G tuberosity offset referred to glenoid"

Radiological parameter(s) (3)

Fracture anatomical reduction

Although the term "fracture malunion" is no longer considered, the steering committee suggests that, only for fractures treated by surgical fixation, a series of binary questions could be asked depending on the fracture pattern in order to assess if the fracture was reduced and/or healed in a position that can be described as anatomical.

Proposed questions are:

- are greater tuberosity and head reduced anatomically? (Yes / No / n.a.*)
- are greater tuberosity and shaft reduced anatomically? (Yes / No / n.a.*)
- are greater and lesser tuberosities reduced anatomically? (Yes / No / n.a.*)
- are lesser tuberosity and head reduced anatomically? (Yes / No / n.a.*)
- are lesser tuberosity and shaft reduced anatomically? (Yes / No / n.a.*)

*n.a. = not applicable, i.e. these two bony entities are not separated by a fracture line.

88% (117/133) agreed with this proposal and set of binary questions

Comments and suggestions

"The challenge will be the imaging to determine this - will require a CT to accurately define these parameters as the tuberosities are 3-D strictures with an ability to be mal reduced in almost any of the 3 planes."

"Anatomical reduction leads to perceived perfection which is difficult to achieve. The committee should define the or range by which it can still be considered anatomical"

"What is anatomical? there might be a small dislocation eg of the greater tub, however which is acceptable. how do we score that?"

"Why asking the same (partial) information by (partially) repetitive questions several times?"

"I really do not like old fashion term like 'anatomical' reduction. Typically it does not correlate well with functional outcome or patients satisfaction except acetabulum fracture perhaps. Should have been good or satisfactory... "

"'Anatomic' is difficult to define and measure and is likely unreliable. "

"Head & shaft reduction is not mentioned"

"Is head shaft angle correct in AP and lateral projection?   Restoration of the medial support, calcar Region?   'eye-balling' = visual judgement: does it look anatomical?"

"To answer the questions physician will spend more time to study the image. Good for training purposes too."

"Sometimes acceptable/best achievable is not necessarily anatomical"

"I find this for the moment most useful, although sometimes difficult to assess."

"Except for head splitting fractures I would recommend using the term neck instead of head.   One also needs to add questions for isolated fractures: - is greater tuberosity well reduced - is humeral neck well reduced - is lesser tuberosity well reduced"

"I would suggest the following: GT - Head GT - shaft LT - Head Head - Shaft"

"Is it possible to show the Hertel classification as basis to ask your binary questions? Seems not very logical now.   "

"Reduction may be binary, but the issue is that some may allow for a range of reduction before they hit a threshold whereby they consider it not anatomic. rather than rely on each person's interpretation of what is 'anatomic', it should be defined. eg. '1. G. Tuberosity is anatomically reduced to head...2. greater tuberosity is within 1 cm of anatomic reduced to head...3. greater tuberosity is more than 1 cm displaced from head.' Again, we should not try to anticipate how much matters and make assumptions about what to measure. Just measure it and later we will find out how it relates to outcome."

"When you say 'Anatomically', have you defined the degree of displacement / Angulation? 1mm? more?"

"Maybe add the degree of 'malreduction'...."

"Not sure what anatomical is. If that means replicating exactly what was before the injury, i don't agree with it"

"Can be done like this but in my opinion REALLY Very extensive"

"How you are able to detect malunion of only one fragment, i.e. secondary dislocation of the greater tuberosity, with binary questions? "

"It may have sense to include: Is articular surface well reduce?"

"I suppose, that you mean for each point: Is the reduction between the one and the other mentioned fragment anatomical. F.e. in the first line: Is the reduction between the greater tuberosity and the head fragment anatomically? I would write it that way in all lines, otherwise it is not clear what is meant. If you change all lines as I mentioned, then line 3 is not necessary (reduction between greater and lesser tuberosity)"

"I would add is the medial hinge restored?"

"No too complicated for judgement and routine research or even clinical use"

"We could add apart from anatomical reduction / if non anatomical is it in valgus and acceptable or varus and at risk of failure / symptoms of impingement"

"I think the existence of rotator cuff at the point of surgery is also important, so recommend to add the question about rotator cuff tear."

"I don't know about the definition of anatomical."

"Are greater tuberosity and head reduced anatomically or close to (<3mm displaced)? (Yes / No / n.a.*) need different threshold for individual combinations"

Radiological parameter(s) (4)

Fracture healing

Definition : the presence of mineralised callus circumferentially around the fracture zone visible on at least two orthogonal radiographs* or postoperative CT.

Specification:

- Delayed healing : the absence of bridging callus on at least one of four cortices in the fracture zone on two orthogonal radiographs taken at 3 months after fracture

- Nonunion** : the absence of bridging callus on at least one of four cortices in the fracture zone on two orthogonal radiographs taken at 6 months after fracture

*a clinician might use three orthogonal views (AP, lateral and axial views - the so-called Neer series), however because this series is not universally achieved in practice, any paired combinations may be considered, i.e. AP and lateral, AP and axial, or lateral and axial combinations

**Nonunion (requiring an additional intervention) is considered an unfavorable event to be documented in the Core Event Set

Note: this definition focuses on externally formed new callus bone i.e. subperiosteal new bone, since internal, endosteal callus is difficult to define. It also takes away the requirement for a clinical definition, since a patient can be in no pain yet have displaced metal work, and another patient can have perfect metal work and still be in pain.

89% (115/129) agreed with this definition, specification and terminology

Comments and suggestions

"I think we should talk about delayed healing after 5 months as i saw in some patients the healing can occur around 4/5 months"

"for this to occur, standardized views must be described which will be used to define union at the distinct cortices. I think delayed healing should be at 6 months and nonunion at 9-12 months otherwise we will be at risk of overcalling delayed union. PHF with significant energy often take 3-6 months longer to heal than low velocity fractures."

"this definition might work well for e.g. cortical shaft fractures, but in my opinion is not sufficient as to describe the diagnose of fracture closure in the humeral head region"

"Radiological diagnosis of union is unreliable. Nonunion is breakage or loosening of the implant and movement at the fracture line. Delayed union is not definable. "

"Delayed union at 3 months is too early. I would not expect all fractures to have healed by 3 months. 4 months maybe for 90%+ We should the internationally agreed definition of nonunion: 'failure of progression of healing of a fracture for 3 months beyond the expected healing time'. We therefore need to agree when we would expect most fractures to have healed by - I would suggest 4-6 months: therefore non-union would be 7-12 months."

"how many cortices are there at the proximal humerus? this is stupid nonsense! sorry!"

"I am concerned on setting a specific length of time to diagnose a delayed healing (Why 3 months and not 4 ando not 5....), and sometimes you can affirm it is a nonunion even earlier by the radiographic characteristics. I believe it would be more useful to specify such radiographic aspects to classify it into a delayed healing and nonunion"

"no suggestion regarding fracture healing"

"Delayed healing = absence of bridging callus on at least 3 out of 4 cortices at 3 months Nonunion = absence of bridging callus on at least 3 out of 4 cortices at 6 months"

"Presence of external bridging callus on XRay in an operated PHFx is frequently difficult to document and can take many months , so for me not a prerequisite for a healed fracture. Definition of nonunion : absence of bridging callus on four cortices . Timing is so complex, 6 months seems a good compromise. I always perform a CT to 'prove' it. "

"this will be hard to apply in a situation that is metaphysical, and not diaphyseal. the assumption of '3 of 4 cortices' relates to the amount of the circumference that is healed, which may predict the amount of healing in the diaphysis, but maybe not is much in the metaphysis. Also, there is a big difference between orthogonal views that are AP/axillary vs AP/Y-view. The reason is that if the fracture is in the transverse plane, the first set of views will not both be images in the plane of the fracture, only the AP is. The transcapular Y-view is, on the other hand, in the lane of the fracture, and so the idea that 2 views shows the circumference of the fracture is true for AP/Y-view, but not for the AP/axillary view. Good reason to require all 3 views for the follow up x-rays"

"But bridging callus in not seen many a times especially in fractures that are not comminuted medially. And does the definition apply for assessing tuberosity healing also?"

"If three cortices are bridged I consider it healed. I don't agree calling this a non-union after 6 months or delayed after 3 months if 3 cortices ARE bridged."

"Bridging callus is not always seen even the fracture is healed. There will be a great interobserver variability."

"Nonunion: absence of bridging callus and sclerosis of fracture surfaces after 9 months."

"Why should be the definition of non-union differ from these we apply at shaft fractures of large bones e.g. tibia. 3 of 4 cortices bridged means healed. thus the absence of callus at least two of four cortices despite one of four cortices, will be my choice as a definition of non-union or delayed healing; timepoints are fine."

"I would define healing, with visible bridging callus formation at 3 of 4 cortices on at least two orthogonal radiographs or CT-scan. Why: 1. In case of plate fixation, one cortex (below the plate) is mostly not really visible, even using 2 or even 3 projections. 2. This Definition is used for most other bones (tibia etc.), why should it be different for the proximal humerus?"

"callus formation is not easy to see. I would rather look for the absence of fracture lines."

"Very difficult to comment on fracture healing without doing a CT Scan and would still propose that a CT Scan review at 3 months if any doubt about union be added to follow up"

"As the proximal humerus is metaphyseal bone you do not expect callus formation, only between metaphysis and shaft. Clinical signs should be taken into consideration to define healing problems. The definition of delayed healing and non-union after 3 and 6 months, I agree with."

Radiological parameter(s) (5)

Bone resorption

Definition : the progressive disappearance of bone from the proximal humerus (either medullary or cortical) when compared to the immediate postoperative or initial non-operative condition, in excess to that expected during normal fracture healing.

Focal specifications :

- epiphyseal (e.g. humeral head avascular necrosis)

- metaphyseal : involvement of the calcar region and/or the tuberosities (greater or lesser)

- diaphyseal : involvement of endosteal or periosteal regions

Note : focal, variable, bone loss is expected at or within fracture regions as healing progresses, but the bone is restored (usually but not always completely) at the completion of healing. Bone resorption to a greater extent than that seen in normal fracture healing may be progressive, permanent (that is, not restored as a result of healing), and non-focal.

98% (127/129) agreed with this definition, specification and terminology

Comments and suggestions

"Shall we call this avascular necrosis "

"EXCLUDE postop infections."

"I'm not happy with the wording epiphyseal, but as regional zones this way of definition might be the best possible/ most realistic"

"I wonder if this is reliable. I bet it's not. "

"I do not agree with these definitions. How should we judge on bone resorption if there is the implant (e.g. PHILOS with up to 9 screws or multiloc) in place?   'bone resorption' is useless in my eyes."

"Very good specifications and definition"

"One needs to differentiate this from halo of bone secondary to loosening (such as halo around proximal locking screws in delayed healing scenario).   Is the definition trying to point out a biologic versus a mechanical problem?"

"you should replace the term avascular necrosis by osteonecrosis"

"Agree, but I think the relevance is unclear."

"In my opinion bone resorption at the tuberosities should be more emphasized und categorized as separate focal specification, as it is observed in plate fixation as well as in shoulder arthroplasty."

"I would specifically and individually address the greater tuberosity"

Radiological parameter(s) (6)

Bone formation

Definition: the progressive apposition of bone on or within the humerus when compared to the immediate postoperative or initial non-operative condition, more than that required for fracture healing (that is, more than the expected extent of callus).

Specifications :

- *Orthotopic bone formation* (ossification) is bone formation within the confines of the bone including the periosteum: bone is formed within tissue that is destined to be or become bone under normal healing or loading conditions (e.g. excessive callus formation)

- *Heterotopic bone formation* (ossification) is a subset of excess bone formation within or between tissues that is not destined to be or become bone under normal healing or loading conditions.

Heterotopic bone formation is classified according to a modified Brooker classification*

Grade 1 = islands of bone within the soft tissues around the shoulder

Grade 2 = bone spurs from the proximal humerus or scapula, leaving at least one centimeter between opposing bone surfaces

Grade 3 = bone spurs from the proximal humerus or scapula, reducing the space between opposing bone surfaces to less than one centimeter

Grade 4 = apparent bone ankylosis of the shoulder

**Brooker AF, Bowerman JW, Robinson RA, Riley Jr LH. Ectopic ossification following total hip replacement: incidence and a method of classification. JBJS 1973;55:1629-1632.*

95% (122/128) agreed with this definition, specification and terminology

Comments and suggestions

"I am unclear as to why we require orthotropic bone formation as this is bone really callus. We should limit the assessment to heterotopic ossification."

"I agree with the definition, and with the terminology, which I don't know if in the case of the proximal humerus, the incorporation of heterotopic ossification evaluation is useful. Because it is low in appearance"

"OK, there is only one place where we will see callus: along the periosteum from the greater tub to the shaft. Topic is still proximal humerus fracture. (see Question 4 and callus/4 cortices... this does not make sense) heterotopic ossification - I agree."

"Rare event."

"How to differentiate bone formation and degenerative bone changes?"

"I think this is of no relevance in the humerus."

"Heterotopic ossifications at the shoulder are extremely rare and do not necessarily have to be considered as a sub-point."

"Sometimes difficult do differentiate between HO and excessive callus. A question whether the bone formations interfere with function might be helpful. Particularly Grade 1 according to the modified Brooker classification might be - by some - regarded as a complication even though it has no impact whatsoever on shoulder function."

AO TRAUMA

Radiological parameter(s) (7)

Humeral head necrosis

Definition: Epiphyseal bone resorption compared to immediate postoperative or initial non-operative radiographs.

Classification: Based on radiographic assessment of the extent of osteonecrosis of the humeral head. Defined as maximum involvement in any radiological view and divided into 5 stages (Cruess classification adapted by Hattrup and Cofield, 1999*):

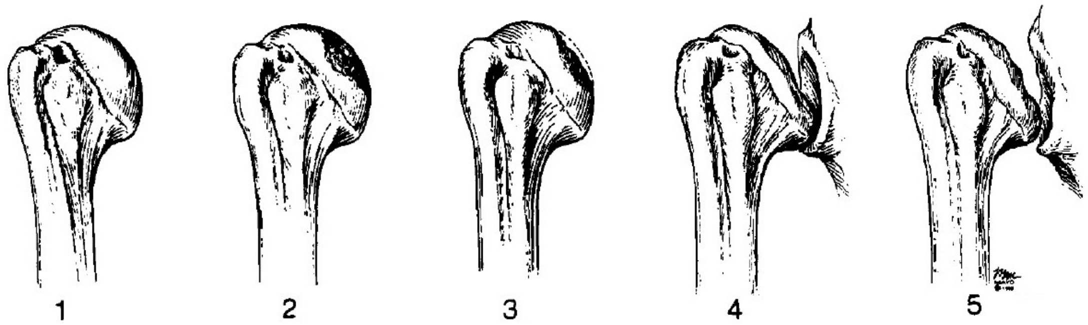

Stage 1: preradiographic

Stage 2: mattled sclerosis

Stage 3: subchondral fracturing

Stage 4: overt collapse of humeral head

Stage 5: presence of glenoid degenerative changes

** Hattrup SJ, Cofield RH. Osteonecrosis of the humeral head: relationship of disease stage, extent, and cause to natural history. Journal of shoulder and elbow surgery. 1999;8(6):559-564*

98% (124/127) agreed with this definition, specification and terminology

Comments and suggestions

"I agree to definition and stage 3 -4 -5. However: Stage 1 This give no meaning if 'diagnosed' by plain radiographs. As MR diagnosis Persistence of bone marrow edema more than 12 months in STIR? Stage 2: Sclerosis may affect more than central area, and possible need to be defined in another way. "

"Shall we not incorporate this into the previous assessment which included bone resorption at the metaphysis, tuberosities and diaphysis."

"I wonder if this is reliable, accurate, or useful. "

"Stage 1: Could be called 'normal bone' or 'no sign of osteonecrosis'. The term 'preradiographic' is misleading: the evaluation is based on radiographic examination. "

"How will you distinguish AVN from early failure (eg in a case with head split)?"

"I would also add location with the HH (quadrant)"

"Head should be divided in zone in AP and Lateral View and Partial AVN should be numbered according to them thus helping in grading the extent of AVN and subsequent collapse "

"nowadays CT or MR may reveal a partial circulatory disturbance of the HH. Would this be disregarded and the diagnosis based on plain radiographs only? What about timing? When is an osteonecrosis manifest?"

"Fitting pts who have had an MR into this can be tricky"

"based on the x-ray at least 3 months after the index surgery."

AO TRAUMA

Postoperative / non-operative local events (1)

Local event groups

The following minor changes shown in red were made by the steering group:

1- Implant (device) events: Events affecting any ~~implanted~~ used device (e.g. nail, plate, prosthesis, external fixator) which are shown on adequate postoperative imaging (e.g. radiographs, ultrasound, CT) or affecting any external device (e.g. sling, orthosis) used to immobilize the fracture, which is associated with clinical symptoms.

99% (128/129) agreed about these minor changes

Comments and suggestions

"include K wires / bone wires in the list of implants."

"I would recommend 'surgically placed device' instead of 'used device'. They way I understand the current phrasing is that events for surgically placed devices are based on imaging, while events for external devices is based on clinical symptoms. I would argue that events should be clinical and radiographic for both, surgically placed implants and external devices. "

"Screws and wires could be added as some fractures are even stabilized by wires and screws without plate or nail"

Postoperative / non-operative local events (2)

Local event groups

The following changes shown in red were made by the steering group:

Surgical implant (device) events

Timeline: the minimum suitable timeline for documenting postoperative implant events should be 12 months.

Non-operative local device events

Definition : Events (e.g. breakage, loosening) involving any external device (e.g. sling, orthesis) used to immobilize the arm to support the fracture, which is associated with local clinical symptoms (e.g. local reactions such as skin lesions).

98% (125/128) agreed about these changes

Comments and suggestions

"I think it should be a minimum of 6 months or until united."

"Stiffness: what is the threshold.   DVT and allergy: how verified? Just surgeon report? Likely inaccurate and unreliable. "

"I am not sure what breakage of sling or orthosis means."

"The local clinical symptom may be the event in itself (i.e. contact dermatitis in an otherwise well placed and functional shoulder immobilizer).   I would leave prior version of nonoperative local device events. "

"I don't understand the term : Breakage or loosening of 'Sling'. it is a mobile on off device, to minimize arm mov, but can be removed for short term, so the term above does not fit"

"Does implant-allergy exist?"

AO TRAUMA

"every question should be regarded in the context of union or not. Fracture union is not included in the list of events above but maybe elsewhere?""

Postoperative / non-operative local events (3)

Osteochondral events (1)

Definition: Events affecting the osteochondral tissue of the proximal humerus, clavicula and/or scapula

Specification :

Surgical treatment only:
- New fracture (around the implant)
- Screw / bolt cutout*

All treatment interventions:
- Bone formation / resorption
- Tuberosity migration / resorption
- Head necrosis
- Delayed union / nonunion
- Loss of fracture reduction
- Other event(s)

Timeline : 24 months

* may be associated with loss of fracture reduction (e.g. head collapse) and/or head necrosis

Note : the event "Fracture malunion" was no longer considered by the steering committee considering that the term reflects negative or inadequate performance. In any treatment one could consider that PHFx are somehow "malreduced", and in non-operative management, one would expect some degree of "malunion". What is to be tolerated for each patient is not well defined and some guidelines (outside the scope of this survey) would be very useful.

97% (122/126) agreed this definition, specifications, timeline and terminology

Comments and suggestions

"see above: difference between cut-out and cut-through (fixation failure vs. head necrosis)"

"I'm concerned that some of these terms are difficult to define and measure and likely unreliable and inaccurate. "

"I agree, but whole consensus should differentiate between screw cutout and intraoperative joint perforation if not diagnosed immediately and recognized later on"

"I think you are still partially missing the point on 'malreduction'. If the surgical indication was to improve the alignment (to improve the reduction), and the fracture is operatively fixed, then it is fair to look at the post op Xray and conclude that: 1. If the fracture is not reduced after the surgery then the surgeon failed, at least in part, in achieving the goals, and 2. If the fracture is reduced in the OR and then changes, and reduction is lost, then we can conclude that this is a negative change."

"for Head necrosis i would recommend a longer timeline : 48 months"

"I would have preferred the 'Malunion' remains and a more accurate definition is described, as it will affect the function and outcome, and will show the difference between Op and Non Op management in outcome."

Postoperative / non-operative local events (4)

Shoulder instability

The following changes shown in red were made by the steering group:

Definition of terms: symptomatic shoulder associated with loss of alignment of the articulating surface of the humeral ~~component~~ head with the ~~articulating~~ glenoid surface ~~of its joint partner~~

Specifications :

- Subluxation : non arm position-dependent eccentric misalignment with residual contact.

- Dislocation : non arm position-dependent complete loss of contact of the articulating surfaces.

- Dynamic instability : arm position-dependent loss of contact of the articulating surfaces apparent on physical examination and/or visible on functional radiographs (horizontal flexion/extension view in 90° of abduction and true AP view in 60° of abduction).

Timeline : 12 months

96% (121/126) agreed with these changes and the proposed timeline

Comments and suggestions

"What about reverse prostheses?"
"Have not seen shoulder instability after PHF!!! Have seen them and treated many."
"How is psuedo-subluxation distinguished? "
"'Subluxation' is poorly defined and difficult to apply clinically. A significant part of patients present with some degree of inferior 'subluxation' especially in the first weeks after trauma."
"I would be happy if the time frame was shortened to 6 months, since instability (in the absence of secondary surgical intervention) is extremely unlikely to occur after 6 months."
"I think this should be at 6 months."
"Does this statement also include subluxations by affected N. Axillares? "
"remember that malalignement can occur independent of time"
"12 months its ok"

Postoperative / non-operative local events (5)

Vascular events

The following changes shown in red were made by the steering group:

Definition: Events involving laceration, avulsion, contusion, puncture or crush injury to an artery or vein ~~or microvascularity~~ at the ~~surgical site~~ injured arm

Specifications :

- Hematoma which requires evacuation by needle or surgery

- Superficial and deep thrombosis at the involved extremity

- Ischemia of the involved extremity which requires additional intervention

Observation period (timeline) : 30 days

98% (124/127) agreed with these changes

Comments and suggestions

"Hematoma?"

"Hematoma arguably never REQUIRES needle or surgery.   DVT are uncommon and difficult to diagnose reliably and accurately. "

"Why 30 days? If we include DVT we should extend the time up-to 6 weeks"

"why just 30 days? if a misplaced screw injures a vessel in long-term...should be also evaluated...although very very low incidence "

"And trombosis after 30 days? "

Postoperative / non-operative local events (6)

Surgical Site Infections (SSI)

Despite a high level of consensus agreement at the first Delphi survey, the steering group suggested however that the recently published consensus on "fracture-related infection" should be adopted*.

"For the purposes of a definition (and data collection), it is important that surgeons define the presence of infection, not its extent, localization or classification."

**Metsemakers WJ, Morgenstern M, McNally MA, et al. Fracture-related infection: A consensus on definition from an international expert group. Injury. 2018;49(3):505-510. PMID: 28867644. doi: 10.1016/j.injury.2017.08.040*

Fracture-related Infections (FRI)

Definition of terms and specifications adopted from the 2018 FRI consensus definition (Metsemakers et al. 2018)

Period of observation: 24 months

98% (124/127) agreed with this recommendation

Comments and suggestions

"I think 12 months will be sufficient."

"this consensus is up till now not validated"

"but describe exactly which confirmatory and/ or suggestive criteria"

"I have a concern about distinguishing colonization (e.g. C. Acnes) from infection. "

"it is reasonable for me"

"12 months. otherwise you are not in schedule with the other copmplications meaning that you always need 24 months FU"

"PERIOD OF OBSERVATION SHOULD BE 3YEARS FROM DATE OF SURGERY. TUBERCULAR INFECTION SURFACES AFTER 2YR USUALLY."

"strongly agree"

Postoperative / non-operative local events (7)

Deep soft tissue events

The following changes shown in red were made by the steering group for a more logical sequence of events, described from an anatomic organization from the articular surface and synovial outwards:

Definition : Events affecting the deep soft tissues (i.e. fascia, muscle, articular capsule), except infections

Specifications :

- External muscular envelope: deltoid-pectoralis major
- Subacromio-deltoid-coracoid bursa (space)
- Rotator cuff muscle-tendon and biceps tendon
- Capsule-synovium

Observation period (timeline): 12 months

97% (122/126) agreed with these changes

There were no further comments or suggestions

Postoperative / non-operative local events (6)

Peripheral neurological events

Superficial soft tissue events

No change was made by the steering group for these two event groups.

Comments and suggestions

"Agree"

"Good"

"OK"

"No change was required"

"OK""
