# Supplementary material for: Core set of unfavorable events of proximal humerus fracture treatment defined by an international Delphi consensus process
Source: BMC Musculoskelet Disord. 2021 Nov 30;22:1002. doi: 10.1186/s12891-021-04887-1 (PMC8630858; doi:10.1186/s12891-021-04887-1)
Supplement: Supplementary file 7 — Additional file 7. PHF Core Event Set v1.0 – Paper-based Form. [file 12891_2021_4887_MOESM7_ESM.pdf]

AO TRAUMA

Supplementary file 7

**Article title**	Core set of unfavorable events of proximal humerus fracture treatment defined by an international Delphi consensus process
**Journal name**	BMC Musculoskeletal Disorders
**Author names**	Audigé L, Brorson S, Durchholz H, Lambert S, Moro F, PHF CES Consensus Panel, Joeris A
**Affiliation**	Schulthess Klinik, CH-8008 Zurich, Switzerland
**E-mail address**	laurent.audige@kws.ch

PHF Core Event Set v1.0

Core list of unfavorable events of proximal humerus fracture (PHF)

Proximal humerus fracture (PHF) unfavorable event form

PHF Core Event Set v1

Proximal humerus fracture (PHF) unfavorable event form

This form has been developed on the basis of a core set of local unfavorable events defined by consensus among an international panel of experienced trauma shoulder specialists. It is recommended for documentation of adverse events / complications in registry settings and clinical studies of proximal humerus fractures. Non-local (rest of the body / systemic) events may be registered by organ system.

Please complete a separate form for each event

Definitions of terms marked by supercript numbers are presented on the next page

1- Intraoperative / “fracture reduction“ event[1]

Local (operated shoulder) >> please record non-local events in section 3 below

- ○ **Device event**[2]
  - ○ Instrument problem *(e.g. breakage, failure, ...)*
  - ○ Implant
    - ○ Breakage
    - ○ Malpositioning
    - ○ Separation
    - ○ Screw/bolt joint surface perforation requiring immediate postoperative surgical revision
    - ○ Other event involving an implant
  - ○ Cementation problems (augmentation)
  - ○ Other device event
- ○ **Osteochondral event**[3]
  - ○ Articular cartilage damage
  - ○ iatrogenic Fracture *(including hairline fracture)*
    - Select all applicable >>> ☐ Humerus metaphyseal[3a] ☐ Humerus diaphyseal ☐ Scapula
  - ○ Other osteochondral event
- ○ **Soft tissue event**[4]
  - ○ Skin, muscle, tendon, joint capsule, ligament, labrum
  - ○ Nerves[4b]
  - ○ Blood vessels (bleeding)[4a]
  - ○ Other soft tissue event
- ○ **Other local intraoperative event**

2- Postoperative / non-operative event[5]

Is there a known date of event occurrence / onset ? ○ No ○ Yes Date : __________________ DD-MM-YYYY

Period of occurrence ○ Postoperatively up to 24h ○ >24h-30d ○ >30d-3mo ○ >3-6mo ○ >6-12mo ○ >12-24mo ○ >24mo

(h = hour; d = day; mo = month)

Local (affected shoulder) >> please record non-local events in section

- ○ **Device event**[6]
  - ○ Malpositioning[6a]
  - ○ Radiolucency around the implant / loosening
  - ○ Screw or bolt backout
  - ○ Breakage
  - ○ Migration[6b]
  - ○ Other device event
- ○ **Osteochondral event**[7]
  - ○ New fracture (around the implant)
  - ○ Screw / bolt cutout
  - ○ Bone formation / resorption[7a]
  - ○ Tuberosity migration / resorption
  - ○ Head necrosis
  - ○ Delayed union / nonunion
  - ○ Loss of fracture reduction[7b]
  - ○ Other osteochondral event
- ○ **Shoulder instability**[9]
  - ○ Subluxation[9a]
  - ○ Dislocation[9b]
  - ○ Dynamic instability[9c]
- ○ **Peripheral neurological event**[10]
  - ○ Sensory and/or motor disturbance
  - ○ Autonomic disturbance: Complex regional pain syndrome
    - ○ Cervical or brachial plexus
    - ○ Branch neuropathy : *Affected nerve(s)* Circle all applicable

Radial Ulnar Median Axillary Suprascapular
Long thoracic Dorsal scapular Thoracodorsal Musculocutaneous
Cervical plexus Spinal accessory Cutaneous nerves

- ○ **Vascular event**[11]
  - ○ Hematoma[11a]
  - ○ Thrombosis at the involved extremity[11b]
  - ○ Ischemia of the involved extremity[11c]
  - ○ Other vascular event
- ○ **Fracture-related infection** (FRI)[12]
- ○ **Superficial soft tissue event**[13]
  - ○ Edema
  - ○ Emphysema
  - ○ Burn
  - ○ Delayed wound healing
  - ○ Hypersensitivity reaction
  - ○ Skin necrosis
  - ○ Skin bulla
  - ○ Hypertrophic scar and keloid[13a]
  - ○ Other superficial soft tissue event
- ○ **Deep soft tissue event**[14]
  - ○ External muscular envelope: deltoid-pectoralis major
  - ○ Subacromio-deltoid-coracoid bursa (space)
  - ○ Rotator cuff muscle-tendon and biceps tendon
  - ○ Capsule-synovium
  - ○ Other deep soft tissue event
- ○ **Other local postoperative event**

3- Non-local (rest of the body / systemic)

- ○ **Intraoperative**
- ○ **Postoperative/nonoperative**

- ○ Perioperative anesthetic event
- ○ Neuro-psychiatric event *(e.g. nausea, delirium, stroke, ...)*
- ○ Pulmonary event *(e.g. respiratory arrest, embolism, ...)*
- ○ Gastrointestinal event
- ○ Other non-local event
- ○ Anaphylactic / allergic reaction
- ○ Cardiovascular event *(e.g. cardiac arrest, DVT, ...)*
- ○ Urinary tract event *(e.g. urinary retention, ...)*
- ○ Musculoskeletal system

Description of the event (only with few keywords) : ____

Detailed description :

Definitions and specifications

Intraoperative / “fracture reduction“ events

1- Intraoperative event = for the core set, the intraoperative period is defined by the operative procedure extending from skin incision to skin closure. An intraoperative event may be treated during the intervention or remain unrecognized until after surgery and, although non symptomatic, trigger a health-related intervention to prevent further negative consequences to the patient
When the fracture is reduced under anaethesia in the context of non-operative management, an equivalent "fracture reduction" period is considered as the time interval between the patient entered the operating room (OR) and the time the patient exited the OR.
2- Events affecting any component of the implanted device or material, or the instrumentation used for their implantation.
3- Events affecting the osteochondral tissue of the proximal humerus, clavicula and/or scapula.
3a- proximal to “surgical neck”
4- Events involving only the soft tissue at the treated shoulder.
4a- Bleeding at the surgical site that requires additional intervention or leads to a stop of the operation
4b- Recognized damage of a neurological structure which needs additional surgical intervention

Postoperative / non-operative events

5- Postoperative / non-operative event = event that occurs or is recognized during the time interval between the date and time that the patient exited the OR and the end of the observation period. The end of the observation period is defined for each event or group of events in the core set. A postoperative event may be a direct consequence of an event that occurred intraoperatively
6- Event affecting the implanted device(s) which is(are) shown on adequate postoperative imaging (e.g. radiographs, ultrasound, MRI) or identified by direct new intraoperative visualization and associated with clinical symptoms.
Non-operative device event = Events (e.g. breakage, loosening) involving any external device (e.g. sling, orthesis) used to immobilize the arm to support the fracture, which is associated with local clinical symptoms (e.g. local reactions such as skin lesions).
6a- Damage, erosion or loss of the articular surface material over time, which is identified by reduction of joint space observed on serial plain radiographs.
6b- Noticeable change of the position of an implant component, relative to the bone to which it is supposedly fixed.
7- Event affecting the osteochondral tissue of the proximal humerus, clavicula and/or scapula.
7a- Excluding tuberosities (see own specification)
7b- Can be determined after assessing the persistence of anatomic reduction of fracture lines between the following anatomic bone structures: greater tuberosity, lesser tuberosity, humeral shaft, and humeral head.
8- Shoulder pain reported by the patient that is not associated with another identified local event (idiopathic) and is either persisting (compared to preoperative status) beyond 6 months postoperatively or worsening anytime postoperatively
8a- Shoulder pain that awakens the patient at night or interferes with sleep
9- Symptomatic shoulder associated with loss of alignment of the articulating surface of the humeral head with the glenoid surface.
9a- Non arm position-dependent eccentric misalignment with residual contact
9b- Non arm position-dependent complete loss of contact of the articulating surfaces
9c- Arm position-dependent loss of contact of the articulating surfaces apparent on physical examination and/or visible on functional radiographs (horizontal flexion/extension view in 90° of abduction and true AP view in 60° of abduction)
10- Events resulting from peripheral neurological injury at the fracture site, which was not present prior to surgery (including worsening of preoperatively known neurological lesion) and which is associated with sensory and/or motor and/or autonomic disturbance.
11- Events involving laceration, avulsion, contusion, puncture or crush injury to an artery, or vein at the injured arm.
11a- Which requires evacuation by needle puncture or surgery
11b- Superficial and deep
11c- Which requires additional intervention
12– as defined by Metsemakers, W.J., et al., Fracture-related infection: A consensus on definition from an international expert group. Injury, 2018. 49(3): p. 505-510.
13- Events affecting the superficial soft tissues (i.e. skin and subcutaneous tissue) at and around the surgical site/wound that do not affect deep soft tissues (i.e. fascia, muscle, articular capsule) and that require additional treatment.
13a- Except if known history of previous development
14- Events affecting the deep soft tissues (i.e. fascia, muscle, articular capsule), except infections.
